# Dinaphthotetrathienoacenes: Synthesis, Characterization, and Applications in Organic Field‐Effect Transistors

**DOI:** 10.1002/advs.202105674

**Published:** 2022-03-16

**Authors:** Rémy Jouclas, Jie Liu, Martina Volpi, Lygia Silva de Moraes, Guillaume Garbay, Nemo McIntosh, Marco Bardini, Vincent Lemaur, Alexandre Vercouter, Christos Gatsios, Federico Modesti, Nicholas Turetta, David Beljonne, Jérôme Cornil, Alan R. Kennedy, Norbert Koch, Peter Erk, Paolo Samorì, Guillaume Schweicher, Yves H. Geerts

**Affiliations:** ^1^ Laboratoire de Chimie des Polymères Faculté des Sciences Université Libre de Bruxelles (ULB) Boulevard du Triomphe, CP 206/01 Bruxelles 1050 Belgium; ^2^ Laboratory for Chemistry of Novel Materials Center for Research in Molecular Electronics and Photonics University of Mons Place du Parc 23 Mons B‐7000 Belgium; ^3^ Helmholtz‐Zentrum Berlin für Materialien und Energie GmbH 12489 Berlin Germany; ^4^ Institut für Physik and IRIS Adlershof Humboldt‐Universitat zu Berlin 12489 Berlin Germany; ^5^ BASF SE RCS – J542S 67056 Ludwigshafen am Rhein Germany; ^6^ University of Strasbourg CNRS ISIS UMR 7006 8 Alleé Gaspard Monge Strasbourg F‐67000 France; ^7^ Dept. of Pure and Applied Chemistry University of Strathclyde Cathedral Street 295 Glasgow G1 1XL UK; ^8^ International Solvay Institutes for Physics and Chemistry Université Libre de Bruxelles (ULB) Boulevard du Triomphe, CP 231 Bruxelles 1050 Belgium

**Keywords:** dinaphthotetrathienoacenes, dynamic disorder, organic field‐effect transistors, organic semiconductors, thienoacenes

## Abstract

The charge transport of crystalline organic semiconductors is limited by dynamic disorder that tends to localize charges. It is the main hurdle to overcome in order to significantly increase charge carrier mobility. An innovative design that combines a chemical structure based on sulfur‐rich thienoacene with a solid‐state herringbone (HB) packing is proposed and the synthesis, physicochemical characterization, and charge transport properties of two new thienoacenes bearing a central tetrathienyl core fused with two external naphthyl rings: naphtho[2,3‐b]thieno‐[2′′′,3′′′:4′′,5′′]thieno[2″,3″:4′,5′]thieno[3′,2′‐b]naphtho[2,3‐b]thiophene (DN4T) and naphtho[1,2‐b]thieno‐[2′′′,3′′′:4′′,5′′]thieno[2′′,3′′:4′,5′]thieno[3′,2′‐b]naphtho[1,2‐b]thiophene are presented. Both compounds crystallize with a HB pattern structure and present transfer integrals ranging from 33 to 99 meV (for the former) within the HB plane of charge transport. Molecular dynamics simulations point toward an efficient resilience of the transfer integrals to the intermolecular sliding motion commonly responsible for strong variations of the electronic coupling in the crystal. Best device performances are reached with DN4T with hole mobility up to *μ* = 2.1 cm^2^ V^−1^s^−1^ in polycrystalline organic field effect transistors, showing the effectiveness of the electronic coupling enabled by the new aromatic core. These promising results pave the way to the design of high‐performing materials based on this new thienoacene, notably through the introduction of alkyl side‐chains.

## Graphical abstract

The synthesis, physicochemical characterization, and charge transport of two new herringbone packing thienoacenes bearing a central tetrathienyl core fused with two external naphthyl rings are presented. Naphtho[2,3‐b]thieno‐[2′′′,3′′′:4′′,5′′]thieno[2″,3″:4′,5′]thieno[3′,2′‐b]naphtho[2,3‐b]thiophene (DN4T) qualifies as one of the best conjugated core for organic field effect transistor applications, thanks to its resilience to dynamic disorder, affording a hole mobility of 2.1 cm^2^ V^−1^s^−1^ in polycrystalline films.

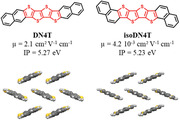

## 1 Introduction

Organic semiconductors for logic operations have generated considerable interest over the last four decades. The first organic field effect transistor (OFET) has been reported by Tsumura, in 1986.^[^ ^1^ ^]^ Performances were rather modest with a charge carrier mobility *μ* = 10^−5^ cm^2^ V^−1^s^−1^ and *I*_on_/*I*_off_ ratio = 10^2^–10^3^. Currently, such figures of merit are considerably higher being on the order of *μ* = 20 cm^2^ V^−1^s^−1^ and *I*_on_/*I*_off_ ratio = 10^6^–10^7^.^[^ ^2^ ^]^ However, mobility levels off and no significant improvements have been seen recently, raising the question of the ultimate performances of organic semiconductors, at room temperature.^[^ ^3^ ^]^ Charge transport is hampered by dynamic disorder that tends to localize charges, as evidenced by *μ* > 100 cm^2^ V^−1^s^−1^ at low temperature or at short time‐ and length‐scales.^[^ ^4^, ^5^, ^6^ ^]^ These encouraging results demonstrate that mobilities as high as *μ* ≈ 100 cm^2^ V^−1^s^−1^ could be reachable at room temperature, if one can design organic semiconductors that are resilient to dynamic disorder. The latter takes roots in thermal agitation, which perturbs the equilibrium position of atoms and molecules, causing a fluctuation of transfer integrals and site energies. Ultimately, a charge localization over a characteristic length occurs, as described by the transient localization scenario.^[^ ^7^, ^8^ ^]^ Each degree of freedom bearing an energy of ½ *k*_B_ *T*, there is no way to prevent molecules to vibrate around their equilibrium position, but strategies exist to prevent positional disorder from turning into dynamic disorder. First, rigid structures reduce large amplitude motions. Second, a weak dependency of transfer integrals on positional disorder is desirable.^[^ ^9^ ^]^ Third, 2D‐isotropic transfer integrals increase the number of *π*‐systems on which charges are delocalized.^[^ ^2^, ^10^ ^]^ Not all intermolecular phonons are equal, low‐frequency, and large amplitude modes contribute more to dynamic disorder.^[^ ^9^ ^]^ Therefore, the design of materials requires both molecular and supramolecular engineering taking into account many requirements. In terms of molecular structures, for instance, large thienoacenes packing in herringbone (HB) motive leads to the highest charge carrier mobility values.^[^ ^11^, ^12^, ^13^ ^]^

**Figure 1:**
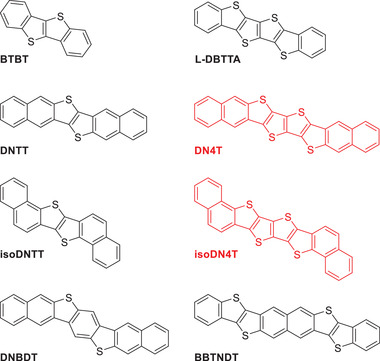
Molecular structures of the target compounds dinaphthotetrathienoacene (DN4T) and isodinaphthotetrathienoacene (isoDN4T) colored in red, and of structurally analogous organic semiconductors previously reported. DN4T, isoDN4T, and BBTNDT are isomers.

In this paper, we contribute to the general understanding of the links between molecular structure, crystal packing, and charge transport with the synthesis and characterization of two new large thienoacene cores: naphtho[2,3‐b]thieno‐[2′′′,3′′′:4′′,5′′]thieno[2″,3″:4′,5′]thieno[3′,2′‐b]naphtho[2,3‐b]thiophene (DN4T) and naphtho[1,2‐b]thieno‐[2′′′,3′′′:4′′,5′′]thieno[2′′,3′′:4′,5′]thieno[3′,2′‐b]naphtho[1,2‐b]thiophene (isoDN4T), displayed in **Figure** **1**. Our goal is to see whether the integration of a central motive comprising four fused thienyl rings, between two naphthyl moieties, leads to high charge carrier mobility in crystal structures preserving HB packing. Previously, linear dibenzoannelated tetrathienoacene (L‐DBTTA) has been reported, but exhibited only *μ* = 0.15 cm^2^ V^−1^s^−1^, and no crystal structure was given.^[^ ^14^, ^15^ ^]^ The shape of thienoacenes is a key design element because it affects translational motions of molecules in crystal structures. In particular, motions along the longest molecular axis are suspected of causing important variations of transfer integrals in the case of HB packing materials.^[^ ^9^, ^16^ ^]^ N‐shape molecular semiconductors, which deviate from linearity, even slightly such as dinaphtho[2,3‐b:2,3‐f]thieno[3,2‐b]‐thiophene (DNTT)^[^ ^17^ ^]^ and bis[1]benzothieno[2,3‐d;2,3‐d]‐naphtho[2,3‐b;6,7‐b]dithiophene (BBTNDT)^[^ ^18^, ^19^ ^]^ decrease such detrimental motions.^[^ ^13^ ^]^ A slight deviation from planarity also seems to favor high charge carrier mobility as evidenced in 3,11‐dioctyldinaphtho[2,3‐d:2,3‐d]benzo[1,2‐b:4,5‐b]dithiophene (C8‐DNBDT‐NW) and 3,11‐didecyldinaphtho[2,3‐d:2,3‐d]benzo[1,2‐b:4,5‐b]dithiophene (C10‐DNBDT‐NW) which are currently among the very best performing molecular semiconductors, with *μ* = 13 and 16 cm^2^ V^−1^s^−1^, respectively.^[^ ^20^, ^21^, ^22^ ^]^ DN4T differs only slightly from DNBDT with the central six‐membered ring being replaced by two fused thienyl rings. Based on this structural analogy, DN4T is also anticipated to lead to high charge carrier mobility in OFETs. A last but important design element comes from the fact that DN4T, isoDN4T, and BBTNDT (*μ* = 5.1 cm^2^ V^−1^s^−1^)^[^ ^18^ ^]^ are isomers, counting as much as 34 *π*‐electrons and differing from the position of fused phenyl and thienyl cycles. IsoDN4T accentuates the N‐shape whereas BBTNDT has a similar shape to DN4T. The comparison of their electronic performances, along with the crystal structure of L‐DBTTA that we also solved for the sake of comparison, will help to establish firm structure‐property relationships to further guide the design of high performing thienoacene‐based organic semiconductors.

## 2 Results and Discussion

### 2.1 Synthesis

**Scheme 1:**
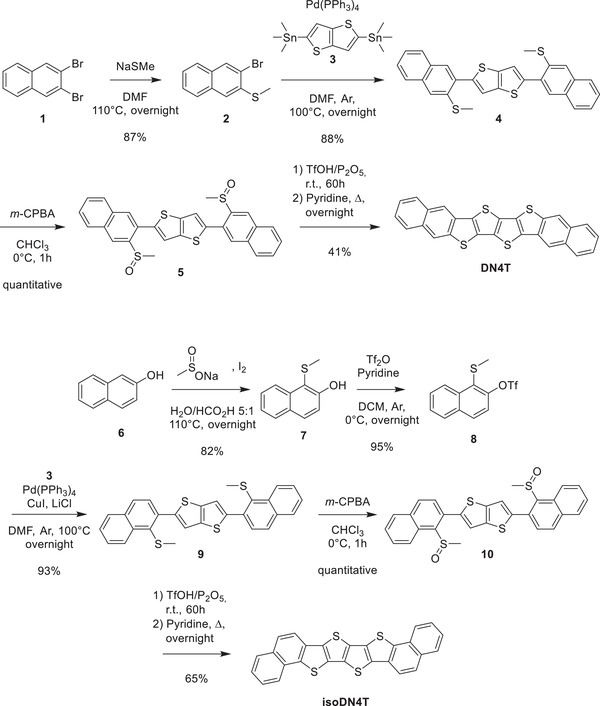
Synthesis of DN4T and isoDN4T.

The target compounds DN4T and isoDN4T were synthesized according to the strategy introduced by Hang et al.^[^ ^14^ ^]^ for the tetrathienoacene core. This synthetic pathway consists in naphthalene functionalization with thiomethyl and halide groups in view of a cross‐coupling with 2,5‐difunctionnalized thieno[3,2‐b]thiophene, followed by subsequent oxidation of the thiomethyl groups into sulfoxides and cyclization into dinaphthotetrathienoacene. The synthesis of DN4T, depicted in **Scheme** **1**, starts with the monomethylthionation of commercial 2,3‐dibromonaphthalene 1, which was performed by using sodium thiomethoxide in refluxed dimethylformamide to afford 2‐bromo‐3‐thiomethylnaphthalene 2 with a good yield. Then the central thieno[3,2‐b]thienyl core of DN4T was introduced by a Stille coupling involving 2 equivalents of 2 with one equivalent of commercial 2,5‐bis‐trimethylstannylthieno[3,2‐b]thiophene 3 to afford 4. The formation of the two last thienyl rings was performed by oxidation of the two thiomethyl groups into sulfoxides using *m*‐CPBA in a quantitative yield followed by subsequent cyclization in triflic acid containing 0.5 equivalent of phosphorus pentoxide and demethylation of the resulting triflate salt in refluxed pyridine according to the procedure of Dong et al.^[^ ^23^ ^]^ to give DN4T with an overall yield of 32%. The synthesis of isoDN4T takes a somewhat similar pathway. Regioselective C‐H methylthionation at the *α*‐ position of commercial *β*‐naphthol 6 was performed according to the procedure of Xiao et al.^[^ ^24^ ^]^ in the presence of sodium methylsulfinate and diiodine to obtain 1‐methylthio‐2‐naphthol 7 by electrophilic substitution through probable in situ Me‐S‐I formation. The end of the synthesis follows the strategy commonly used by Takimiya et al.^[^ ^12^, ^25^, ^26^ ^]^ with triflation of *β*‐hydroxy group to give 8. Stille cross‐coupling with 2,5‐bis‐trimethylstannylthieno[3,2‐b]thiphene was performed in the presence of CuI as a ligand scavenger for Pd(PPh_3_)_4_^[^ ^27^, ^28^ ^]^ and LiCl to favor triflate insertion on the catalyst^[^ ^28^, ^29^ ^]^ to obtain 9 in good yields. Like in DN4T, 2‐steps cyclization of disulfoxide 10 in TfOH/P_2_O_5_ mixture followed by demethylation in refluxed pyridine gave isoDN4T with an overall yield of 44%. Thanks to optimized reactions conditions, DN4T and isoDN4T were obtained with 31% and 47% yield, respectively, and in a quantity sufficient for further studies.

### 2.2 Assessment of Molecular Structure and Purity

The intermediate compounds depicted in Scheme 1 were easily purified and characterized by usual methods (see Section S1, Supporting Information). However, the low solubility of DN4T and isoDN4T led to low resolution NMR signals in solution. Their proton NMR analysis were performed in deuterated 1,1,2,2‐tetrachloroethane at 120 °C and are consistent with the reported spectra of DNTT^[^ ^17^ ^]^ and isoDNTT.^[^ ^30^ ^]^ The higher solubility of isoDN4T enabled us to perform a TOCSY 1D ^1^H NMR directly on this compound to assign its groups of protons to the corresponding ^1^H NMR signals (Figure S1, Supporting Information). As the ^1^H NMR spectra of DNTT and DN4T are almost identical, we performed a COSY ^1^H NMR experiment on DNTT (Figure S2, Supporting Information) to attribute the groups of protons of DN4T by comparison (Figure S3, Supporting Information). Importantly, ^1H^ NMR spectra reveals the absence of soluble organic impurities in saturated solutions. High‐resolution matrix‐assisted laser desorption ionization mass spectrometry has been performed to assess the structure of the target compounds. DN4T and isoDN4T exhibit *m*/*z* ratio of 451.9821 and 451.9814 uma, well matching the calculated value of 451.9822. UV–vis spectra confirm the *π*‐delocalized electronic structures of DN4T and isoDN4T with maximum absorption peaks at 448 and 348 nm, respectively (measurements on thin films) (Figure S4, Supporting Information). The decisive results that prove the identity of the target compounds come from crystal structures (vide infra). The purity of sublimed DN4T and isoDN4T is corroborated by thermogravimetric analysis (Figure S10, Supporting Information). In fact, no weight loss was observed before 400 °C, which could result from the sublimation, evaporation, and/or degradation of low molecular weight impurities, has been observed. In addition, XPS measurements on DN4T and isoDN4T deposited on gold substrates allowed us to evaluate the C/S ratio and to check the absence of other elements excluding gold, giving further evidence of the materials’ purities (see Section S6.2, Supporting Information). Experimental C/S values calculated from the XPS spectra are 6.4 ± 0.8 for DN4T and 6.9 ± 1.0 for isoDN4T, lying within the interval of confidence respect to the expected value of 6.5 for both isomers. Last, no other elements except gold were found indicating that semiconductors are devoid of side products, catalyst residue, or solvent traces.

### 2.3 Crystal Structures

**Figure 2:**
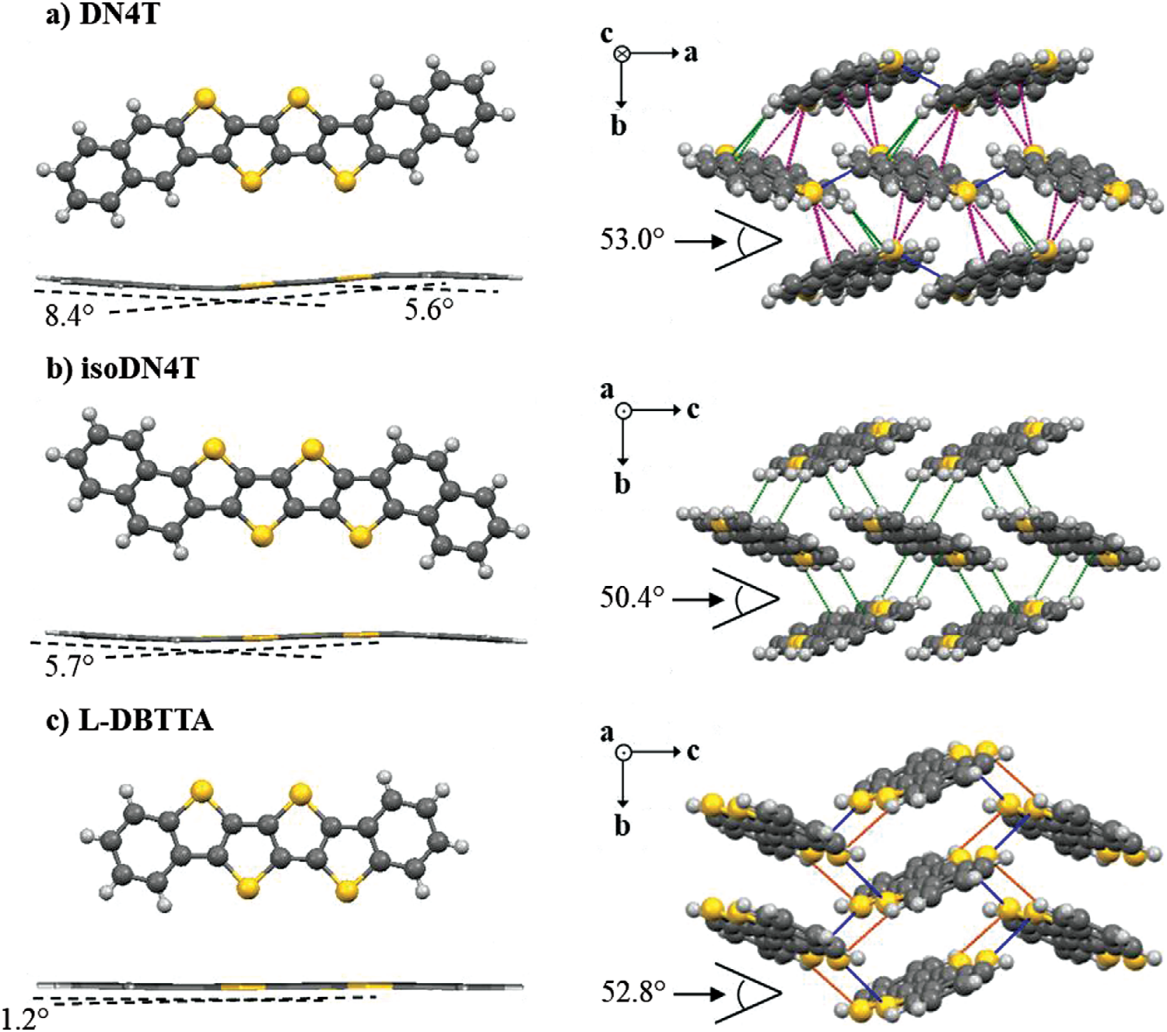
Crystal packing diagrams of a) DN4T, b) isoDN4T, and c) L‐DBTTA. Short contacts (S···S in blue, C···S in purple, C···H in green, and S···H in orange) are displayed as distances less or equal to the sum of van der Waals radii. Intramolecular angles are calculated as the angles between the mean planes of the external phenyl rings and the tetrathienyl core.

Single crystals of DN4T and isoDN4T were obtained by physical vapor transport method through sublimation of the materials under vacuum. DN4T belongs to monoclinic space group *P* 2_1_ (Table S1, Supporting Information) with one unique molecule (*Z*′ = 1) in the asymmetric unit (Figure S5, Supporting Information). Further information regarding the symmetry of the crystal structure of DN4T can be found in Section S3.1, Supporting Information. In contrast, isoDN4T crystallizes in space group *P* 2_1_/*c* with one molecule lying on an inversion center, giving *Z*′ = 0.5. Both molecules deviate from planarity with a pair of bend angles of 5.6°/8.4° for DN4T and 5.7° for isoDN4T (**Figure** **2**) which may favor lower amplitude molecular motions and stronger intermolecular interactions as suggested by previous reports on C_10_‐DNBDT‐NW^[^ ^20^ ^]^ and C_10_‐DNT‐VW.^[^ ^31^ ^]^ DN4T and isoDN4T assume a layer‐by‐layer HB packing structure with respective HB angles between two adjacent molecules of 53.0° and 50.4° in the typical range of common thienoacenes assuming the same crystal pattern.^[^ ^18^, ^32^, ^33^ ^]^

Single crystals of L‐DBTTA obtained by recrystallization in chlorobenzene enabled us to solve its still unknown crystal structure, which is worth comparing to the structurally related DN4T and isoDN4T, that keep their tetrathienyl core and two adjacent phenyl rings unchanged. This molecule crystallizes into space group *P* 2_1_/*c* assuming a stacked packing (Figure 2), but still with an angle between two non‐parallel adjacent molecules of 52.8° in the range of the HB angles of DN4T (53.0°) and isoDN4T (50.4°) and one molecule lying on an inversion center giving *Z*′ = 0.5 (Figure S5, Supporting Information).

**Figure 3:**
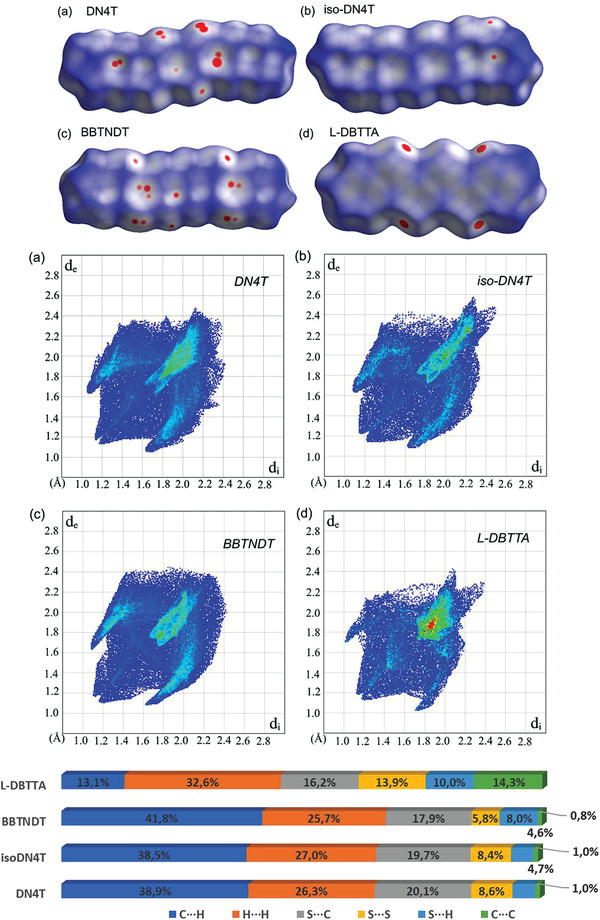
Hirshfeld surfaces and fingerprint plots of a) DN4T, b) isoDN4T, c) BBTNDT, and d) L‐DBTTA, and their corresponding relative contributions to the HS areas.

As displayed in Figure 2, DN4T connects with six adjacent molecules through ten different C—H···*π*, S···C and S···S short contacts, a configuration that is very similar to BBTNDT^[^ ^18^ ^]^ which connects with as many neighbors through six more short contacts incorporating one more S···S than DN4T. In comparison, the BTBT molecule which is comprised of exactly half the number of phenyl and thienyl rings connects through four close contacts with the nearest molecules,^[^ ^32^ ^]^ which is less than half of the number of DN4T and BBTNDT ones. Considering isoDN4T, only two C—H···*π* short contacts enable the connection with four adjacent molecules, likely playing for fewer and less efficient pathways for electron transfer. These disparities in the intermolecular contacts are further depicted by the corresponding Hirshfeld surfaces (HS, **Figure** **3**), showing on a single molecule its weighted interactions with the adjacent ones.^[^ ^34^, ^35^ ^]^ DN4T and BBTNDT^[^ ^35^ ^]^ show numerous bright red spots on the front and back sides of their HS, particularly involving face‐to‐edge interactions through their thienyl rings, contrarily to isoDN4T. Calculation of the volume enclosed in the HS also gives evidence of a slightly more compact packing for DN4T and BBTNDT than for isoDN4T (respectively 459.7 and 461.2 Å^3^ vs 467.9 Å^3^), supporting the abovementioned considerations. Finally, a look at the fingerprint plots and relative contributions to the HS of DN4T, isoDN4T, and their first published isomer BBTNDT^[^ ^18^, ^35^ ^]^ reveals an almost identical packing environment for the three molecules, with interestingly less S···S contributions to the HS in BBTNDT despite strong differences in terms of short contacts. Comparison with L‐DBTTA confirms the importance of the *π*···*π*‐stacking and edge‐to‐edge interactions resulting in its stacked packing structure, depicted by the much higher H···H, C···C, and S···S contributions than CH···*π* with respect to DN4T, isoDN4T, and BBTNDT (Figure 3).

These results suggest that a smart balance between thienyl and phenyl rings, and particularly between face‐to‐face and face‐to‐edge interactions has to be reached when designing OSCs in view of obtaining a HB packing.^[^ ^36^ ^]^ The global molecular shape also plays an important role as suggested by the comparison of isoDNTT (sandwich HB) with isoDN4T (HB), even though all these considerations are still not enough to explain the preferred crystal packing of DNBDT (slip‐stacked) bearing a central phenyl ring instead of the central thieno[3,2‐b]thienyl of DN4T.

As the crystal structures obtained by single crystal X‐ray diffraction (SCXRD) were collected at low temperature (DN4T at 173 K and isoDN4T at 100 K), X‐ray powder diffraction patterns were collected at different temperatures from 93 to 303 K for DN4T and isoDN4T, indexed and refined to map the thermal expansion and possible phase transitions between low and room temperatures of DN4T and isoDN4T.^[^ ^37^ ^]^ When plotting the values of each unit cell parameter (*a*, *b*, *c*, and *β*) for each temperature point (Figure S9, Supporting Information), it is possible to observe that the changes of lattice parameters of both compounds are insignificant (<1.5% of difference). Importantly, there are no phase transitions or great variations of the unit cell between low and room temperatures for either DN4T or isoDN4T. No phase transitions have also been detected by differential scanning calorimetry up to 300 °C (Figure S11, Supporting Information). Thus, DN4T and isoDN4T qualify as ideal systems for studies over a temperature range as large as 500 K, which are necessary for charge transport elucidation. In this endeavor, DN4T and isoDN4T robustness against polymorphism is definitively an asset.

### 2.4 Quantum Calculations

**Figure 4:**
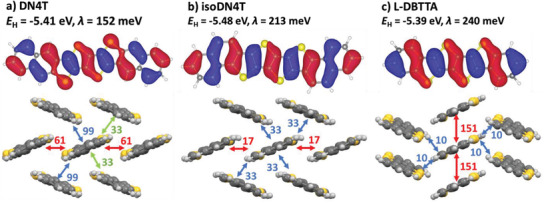
Energy (*E*_H_) and shape of the HOMO orbital of a) DN4T, b) isoDN4T, and c) L‐DBTTA calculated by DFT after geometry optimization at the B3LYP/ 6–311G* level and intermolecular transfer integrals (in meV) calculated from the crystal structure. Calculation of the reorganization energy *λ* of DN4T and isoDN4T can be found in Section S5.2, Supporting Information.

HOMO orbitals have been calculated by density functional theory (DFT), after geometry optimization in gas phase at the B3LYP 6–311G* level,^[^ ^38^ ^]^ and transfer integrals were calculated from the experimental SCXRD results at the B3LYP functional and DZ basis set.^[^ ^39^ ^]^ The bonding–antibonding patterns of the HOMO orbitals of DN4T and isoDN4T look very similar to the corresponding data in DNTT and isoDNTT^[^ ^40^ ^]^ (**Figure** **4**). Most importantly, the two isomers show very different electron densities on the sulfur atoms, large in DN4T but null in isoDN4T, which can be attributed to the “phene‐like” structure of isoDN4T compared to the straight “acene‐like” structure of DN4T. As a matter of fact, the hole reorganization energies *λ* were found to be 213 meV for isoDN4T and 152 meV for DN4T, which is consistent with the ≈60 meV difference found between DNTT and isoDNTT^[^ ^40^ ^]^ and should thus favor hole transport in DN4T within a pure hopping regime. Conversely, L‐DBTTA shows a very similar HOMO shape as DN4T, but with a much higher *λ* ^[^ ^14^ ^]^ compared to DN4T and DNTT^[^ ^40^ ^]^ (240 vs 152 and 130 meV, respectively) which is consistent with the fact that phenyl rings can reduce *λ* more than thienyl ones.^[^ ^41^ ^]^ Electronic couplings in the crystal structure are overall larger in DN4T compared to isoDN4T resulting from the abundance of short contacts involving sulfur atoms (Figures 2, 3) and the larger electronic density over them in the HOMO of DN4T. Further comparison of the orbital overlap between adjacent parallel dimers in DN4T and isoDN4T crystal structures, as well as between the two other dimers of DN4T counting for its better transfer integral values can be found in Figures S13,S14, Supporting Information. Regarding L‐DBTTA, its transfer integrals distribution clearly suggests a 1D electron pathway, thus explaining the low charge carrier mobility previously reported on polycrystalline OFETs.^[^ ^14^ ^]^

**Figure 5:**
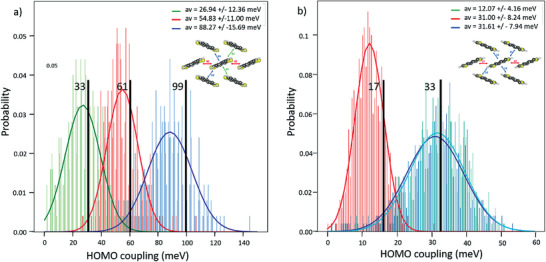
Electronic coupling distribution of a) DN4T dimers and b) isoDN4T dimers in the herringbone plane (500 snapshots). The electronic coupling values of each dimer in the experimental structure are shown by a black vertical bar.

Molecular dynamics simulations revealed that the standard deviations of transfer integral variations in DN4T are in the 10–16 meV range (**Table** **1**), which represents 45%, 20%, and 17% (for the green, red, and blue dimers, respectively, **Figure** **5**) of the mean electronic coupling. This is, overall, low compared to state‐of‐the‐art materials (pentacene: 50–100%, anthracene: 40–60% at 300 K,^[^ ^42^ ^]^ rubrene: 34–43%,^[^ ^43^ ^]^ and DNTT: 30–70%^[^ ^44^ ^]^). In isoDN4T, they represent around 25% of the mean value for the couplings in the blue dimers and 34% of the mean value for the coupling in the red dimer. As expected, the two dimers forming a HB angle along a given diagonal behave much alike since there is an inversion center in the unit cell of isoDN4T.

**Table 1:** Electronic couplings in DN4T and isoDN4T in the crystal structure and averaged over a MD simulation run (of 3 ns) together with the corresponding standard deviation

Dimer	Crystal structure transfer integral [meV]	MD mean transfer integral [meV]	MD standard deviation [meV]
DN4T green	33	27	12
DN4T red	61	55	11
DN4T blue	99	88	16
isoDN4T red	17	12	4
isoDN4T light blue	33	31	8
isoDN4T dark blue	33	34	8

**Figure 6:**
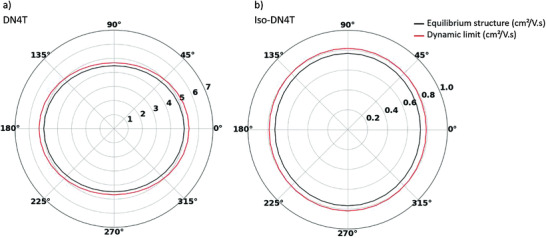
Mobility anisotropy plots of a) DN4T and b) isoDN4T in the equilibrium geometry (black) and dynamic limit (red) along a molecular dynamics run in the NVT ensemble at 300 K obtained from the kMC simulations.

In a first attempt to assess how the different connectivity in DN4T versus isoDN4T affects charge transport, we have performed kinetic Monte Carlo (kMC) simulations of hole transport assuming a purely hopping regime in the dynamic limit, in the framework of the Marcus–Levich–Jortner formalism.^[^ ^45^ ^]^ The detailed procedure as well as approximations and parameters used for this simulation can be found in Section S5.5, Supporting Information. The maps of the mobility anisotropy within the HB plane of DN4T and isoDN4T at the dynamic limit (defined as the maximum mobility the charge can reach in this framework) and at the equilibrium structure are depicted in **Figure** **6**.

As expected from the relative magnitude of the transfer integrals, the single‐crystal hole mobility is higher in DN4T (≈5 cm^2^ V^−1^s^−1^) than in isoDN4T (≈0.8 cm^2^ V^−1^s^−1^). The highest mobility for DN4T is obtained at 180° and 0° (along the *a* axis) which corresponds to the 60 meV electronic coupling (in red, Figure 5a). The minimal values of mobility at 90° and 270° (*b* axis) are associated with the direction along the 99 and 33 meV dimers (in blue and green, Figure 5a). In the case of isoDN4T, the maximum mobility corresponds to the direction along the two 33 meV dimers (*a* axis), and the minimum, to the 17 meV, *b* axis (in blue and red, respectively, Figure 5b). The anisotropy of the mobility within the HB layer is relatively mild as both parameters drop around 10% of their maximum value for their respective minimal mobility direction. Hence, crystal island orientation with respect to the source–drain electric field will have very little impact on the mobility of the device. Overall, the mobility that arises from the mean couplings of the NVT run and the dynamic limit provide a rough estimate of the charge transport performances in the DN4T and isoDN4T crystals although the charge carrier mobility in DN4T single crystals would be better described by the transient localization model.

### 2.5 Charge Transport Measurements

Ionization energy (IE) values of DN4T and isoDN4T were measured by photoelectron yield spectroscopy (PYS) on powder samples under ambient conditions and by ultraviolet photoelectron spectroscopy (UPS) as well, at 10^−10^ bar on thin films, grown on Au and PEDOT:PSS substrates, composed of lying and standing semiconductor molecules, respectively (see Section S6, Supporting Information). PYS provided average values for the IEs, with DN4T (5.27 ± 0.02 eV) and isoDN4T (5.23 ± 0.01 eV), lying between the IEs of flat‐lying and standing molecular orientations as extracted from the UPS experiments (see Table S3, Supporting Information), which is attributed to the random molecular orientation of the molecules in powders. Moreover, the reported IE values are in good agreement with the theoretical HOMO orbital energies calculated in vacuum by DFT (Figure 4). A low injection barrier for transistor operation with commonly used gold electrodes can be expected from these very similar values, which are consistent with the reported IE values of fully aromatic organic semiconductors, slightly lower than DNTT (5.4 eV)^[^ ^12^ ^]^ and DNBDT (5.45 eV)^[^ ^20^ ^]^ likely due to a higher *π*‐extension but still higher than their closest isomer BBTNDT (5.15 eV).^[^ ^18^ ^]^

To investigate the electrical performances of the DN4T isomers, thin‐film transistors (TFTs) with bottom‐gate top‐contact (hereinafter shortened as TC) and top‐gate bottom‐contact (hereinafter shortened as BC) configurations were fabricated through thermal evaporation in high vacuum on highly doped silicon wafer substrates at different temperatures (see Section S8, Supporting Information).

**Figure 7:**
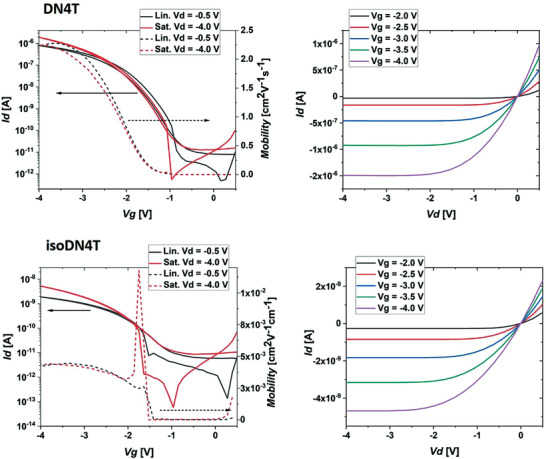
Transfer (left) and output (right) characteristics of DN4T (top) and isoDN4T (bottom) of TFTs fabricated with TC structure on substrates at a temperature of 100 °C. In the transfer curves the solid lines represent the drain current and the dashed lines represents the mobility. The TFTs have *W*/*L* = 480/215 µm.

Best performing devices were obtained with TC architecture with mobility values consistent with the BC ones (see Section S7, Supporting Information). The main performance parameters of the TC devices in the linear regime are reported in **Table** **2** as well as transfer and outputs characteristics in **Figure** **7**.

**Table 2:** Performances of TFTs based on DN4T and isoDN4T with bottom‐gate top‐contact (TC) architecture. All the values are referred to the linear regime (*V*_d_ = −0.5 V)

Compound	Sub. temperature [°C]	Mobility [cm^2^ V^−1^s^−1^]	*V*_th_ [V]	*I*_on_/*I*_off_
DN4T	40	0.9 ± 0.03	−2.3 ± 0.01	≈5 × 10^5^
	60	1.7 ± 0.03	−2.0 ± 0.05	≈2 × 10^6^
	80	1.7 ± 0.08	−2.4 ± 0.07	≈7 × 10^5^
	100	2.1 ± 0.03	−1.9 ± 0.04	≈9 × 10^5^
	120	1.9 ± 0.22	−1.9 ± 0.10	≈10^6^
	140	1.9 ± 0.05	−2.1 ± 0.13	≈9 × 10^5^
isoDN4T	40	(3.6 ± 0.06) × 10^−3^	−2.0 ± 0.05	≈6 × 10^3^
	60	(3.6 ± 0.08) × 10^−3^	−1.9 ± 0.07	≈7 × 10^3^
	80	(3.7 ± 0.01) × 10^−3^	−1.8 ± 0.07	≈4 × 10^3^
	100	(4.2 ± 0.01) × 10^−3^	−1.7 ± 0.01	≈4 × 10^3^
	120	(4.1 ± 0.05) × 10^−3^	−1.8 ± 0.03	≈4 × 10^3^
	140	(3.5 ± 0.04) × 10^−3^	−2.2 ± 0.04	≈3 × 10^3^

Charge carrier mobilities ranging from 0.9 to 2.1 cm^2^ V^−1^s^−1^ and on/off current ratios (*I*_on_/*I*_off_) of ≈10^5^–10^6^ could be extracted for DN4T, which is in the same order of magnitude as the reported mobility of BBTNDT.^[^ ^18^ ^]^ IsoDN4T devices reached mobilities of 3.0–4.2 × 10^−3^ cm^2^ V^−1^s^−1^ and *I*_on_/*I*_off_ between ≈10^2^ and 10^3^ with surprisingly the same lowering by three orders of magnitude in charge carrier mobility as reported from DNTT to isoDNTT devices, along with similar mobility values,^[^ ^17^, ^40^ ^]^ despite the different molecular packing of the latter. The highest values were achieved for films produced using a substrate temperature of 100 °C during the deposition for both DN4T and isoDN4T, due to larger grain size. All thin films also exhibited the same single‐crystal phase as the solved one and no thin‐film polymorphism was observed through XRD characterization (see Section S8.3, Supporting Information).

## 3 Discussion

The synthesis of DN4T and isoDN4T is as simple as that of DNTT, DNBDT, and BBTNDT due to their similar chemical structures. Both DN4T and isoDN4T can be synthesized in a few steps and in good overall yields, which enables their preparation in ≈0.5 g‐scale. These compounds are thermally stable up to 400 °C and can be sublimed under vacuum. They crystallize easily on a substrate upon deposition from vapor phase and show no thin‐film polymorphism. For ease of comparison, most of the properties having an influence on the charge carrier mobility of the thienoacene semiconductors of this work—except the sensitivity to dynamic disorder that will be discussed later—are presented in **Table** **3**. As one of the main issues when comparing the performances of semiconductors comes from the variability of the values resulting from the variability of the methods used to measure or calculate them, all IE values presented in this table have been measured either by PYS or UPS (both techniques giving consistent results), except for isoDNTT (cyclic voltammetry). Moreover, even though a qualitative comparison of the transfer integral distributions (high vs low, balanced vs clearly 1D) can be done regardless of the calculation method, one look at the different set of values obtained for BBTNDT depending on the functional and basis set used for calculation highlights the necessity of using the same method for quantitative analysis. Last, as the measurement of the charge carrier mobility is often subjected to bias due to contact resistance and thin‐film morphology,^[^ ^2^ ^]^ a fair comparison should thus be limited to their orders of magnitude.

**Table 3:** Structural and electronic properties of DN4T and isoDN4T benchmarked with those of structurally related thienoacenes. All transfer integrals mentioned with (ADF) have been calculated using the Amsterdam Density Functional package with a PW91 functional and a TZP basis set. Data with a (*) correspond to measurements/calculations performed in this work: transfer integrals were calculated using the Amsterdam Density Functional package with a B3LYP functional and DZ basis set, and the reorganization energy of DNBDT was calculated with the Gaussian package, MP2 functional, and 6–31(d,p) basis set. Mobility values mentioned with (SC) have been measured on single crystals

Compound	Packing	IE [eV]	*λ* [meV]	*J* [meV]	*μ* [cm^2^ V^−1^s^−1^]
BTBT	Herringbone	5.64^[^ ^19^ ^]^	230^[^ ^46^ ^]^	60, 23, 23^[^ ^12^ ^]^ (ADF)	0.032^[^ ^47^ ^]^ (SC)
DNTT	Herringbone	5.4^[^ ^12^ ^]^	130^[^ ^40^, ^44^ ^]^	91, 71, 14^[^ ^12^, ^40^ ^]^ (ADF)	2.0–2.1^[^ ^48^, ^49^ ^]^ 7–8.3^[^ ^33^, ^50^ ^]^ (SC)
isoDNTT	Sandwich herringbone	5.58^[^ ^40^ ^]^	192^[^ ^40^ ^]^	172, 2, 0.1, 6^[^ ^40^ ^]^ (ADF)	(10^−3^–10^−2^)^[^ ^40^ ^]^
DNBDT	Slip‐stacked	5.45^[^ ^20^ ^]^	519 (*)	3, 3, 3, 4, 24^[^ ^20^ ^]^ (ADF)	0.05^[^ ^20^ ^]^
L‐DBTTA	Stacked	5.16 (*)	240^[^ ^14^ ^]^	151, 10, 10 (*)	0.15^[^ ^14^ ^]^
DN4T	Herringbone	5.27 (*)	152 (*)	61, 99, 33 (*)	2.1 (*)
isoDN4T	Herringbone	5.23 (*)	213 (*)	17, 33, 33 (*)	4.2 × 10^−3^ (*)
BBTNDT	Herringbone	5.15^[^ ^12^, ^19^ ^]^	123^[^ ^35^ ^]^	75, 32, 41 (ADF) 98, 66, 52 (*)	5.1^[^ ^18^ ^]^

First of all, the data presented in Table 3 support the commonly accepted conclusion that large and balanced transfer integral distribution—which is highly dependent on the packing—is necessary to reach mobility values in the order of 1 cm^2^ V^−1^s^−1^. Regarding this aspect, the crystal structure comparison of L‐DBTTA with DN4T and isoDN4T shows that the lack of face‐to‐edge interactions like CH···*π* (in DN4T and isoDN4T) with respect to *π*···*π* stacking and edge‐to‐edge interactions (in L‐DBTTA) tends to pack the molecules into columns, thus leading to a strongly 1D transfer integrals distribution, detrimental to charge transport (as confirmed by its rather poor performances). This is nevertheless compensated in DN4T and isoDN4T through the replacement of the external phenyl rings of L‐DBTTA by naphthyl moieties that enables a favorable HB packing while improving the homogeneity of the transfer integrals distribution. In other words, the use of a central tetrathienyl core can lead to effective electronic coupling in the crystal, provided a HB crystal packing with several fused phenyl rings bringing enough CH···*π* interactions, and a central straight linear shape keeping high electron density on the sulfur atoms and lower reorganization energy. Further comparison of BTBT with DNTT, DN4T, and BBTNDT from Table 3 also confirms that low reorganization energy is an important aspect to increase the charge carrier mobility, which can be obtained through linear *π*‐conjugation extension and preference of “acene” structures to “phene” ones. In addition, a smart balance between thienyl and phenyl rings as well as a careful linear arrangement must be applied to decrease *λ*.^[^ ^41^ ^]^ Moreover, the IE of these materials clearly scales with their ability to stabilize charges through delocalization (at least for HB compounds), which is directly related to the availability of a large and balanced transfer integral distribution. As the access to a low injection barrier is a requisite to high mobility, it is of course logical to observe materials with the lowest IE values providing the highest charge carrier mobilities.

**Figure 8:**
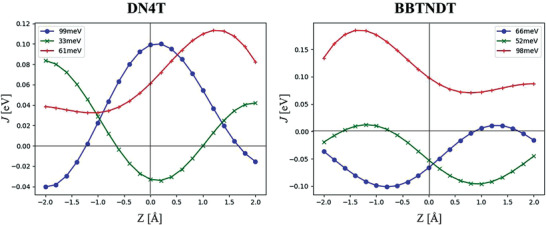
Variation of the transfer integrals of DN4T (left) and BBTNDT (right) as a function of the displacement along the long axis of the molecules.

At this stage, the electron–phonon coupling has to be taken into account to further compare the remaining best performing thienoacene cores (from Table 3) discussed in this study, namely DNTT, DN4T, and BBTNDT. Although proper comparison of their sensitivity to dynamic disorder should be carried out through a comprehensive analysis of the influence of all the vibration modes to which the molecules are subjected in the crystals and assess their influence on the transfer integrals, we chose to reduce the large scope of this study to the sliding motion along the long axis of the molecules that is often the most detrimental to charge transport in HB packing materials and particularly in non‐alkylated aromatic cores.^[^ ^9^ ^]^ We thus computed the variation of the electronic coupling between the three inequivalent dimers in the HB plane of DN4T and BBTNDT as a function of the displacement along the long axis of the molecules (**Figure** **8**), excluding DNTT for its less balanced transfer integral distribution and to limit this study to structural isomers for more accurate interpretations. DN4T has two transfer integrals close to a “sweet spot” contrary to BBTNDT whose transfer integrals are all rather close to an inflection spot, which favors a better resilience of the former to dynamic disorder. Two major aspects that can explain these differences in terms of electron–phonon coupling can first come from the difference in the HOMO shape of these two molecules : the HOMO shape of DN4T is more homogeneously spread on the molecule than in BBTNDT whose HOMO shape is more concentrated on its center.^[^ ^18^ ^]^ On the other hand, the assembly of the thienyl rings that participate more than phenyl rings to the electron transfer in DN4T can also favor softer variations of the electronic couplings as a function of the vibration.

Best charge transport properties in polycrystalline thin‐films are achieved for materials exhibiting large and balanced transfer integrals (in agreement with the transient localized mechanism), namely DNTT, DN4T, and BBTNDT. These materials exhibit charge carrier mobility values of the same order of magnitude with slight fluctuations resulting from the delicate balance of crucial parameters (grain size, balance, and value of *J*, IE, *λ*, and dynamic disorder). It is also worth mentioning that the experimental value obtained for DN4T and isoDN4T is in good agreement with theoretical calculations. While the modeling correctly reproduces the trend going from DN4T to isoDN4T, the mobility values measured in isoDN4T are two orders of magnitude smaller than the theoretical results, a discrepancy likely reflecting strong scattering at grain boundaries, larger contact resistance or dynamic disorder, or a combination of these effects (see Section S8.2, Supporting Information).

## 4 Conclusion

From this body of work, it sounds thus logical that to climb the mobility ladder, one has to design a material exhibiting a HB packing with large and balanced transfer integrals. This will also further allow the material to stabilize charges hrough delocalization and hence access lower IE values, mandatory for efficient charge injection at the electrodes. Linear *π*‐extension of the aromatic core, preferring “acene” structures to “phene” ones, will also provide lower reorganization energies for the system. Finally, a quick evaluation of the impact of the long axis sliding motion suggests that a proper engineering of the HOMO shape aiming for a homogeneous spread over the full molecule through “acene” and “phene” moieties tends to favor a better resilience to dynamic disorder. Considering this aspect and the achievement of mobility values of the same order of magnitude for DN4T and BBTNDT within a thin‐film configuration, we conclude that DN4T qualifies as one of the best conjugated cores for OFET applications. Another distinct feature of DN4T is the central fused tetrathienyl moiety that was already present in L‐DBTTA, affording similar HOMO shapes, for both aromatic cores. However, the former forms a favorable HB packing, whereas the latter adopts a stacked motive that leads to unbalanced transfer integrals. Central fused tetrathienyl moiety appears thus an interesting molecular design element, provided that HB packing is maintained. Furthermore, the evaluation of the influence of the sliding modes of DN4T and BBTNDT on their transfer integrals tends to show that the central tetrathienyl core of DN4T helps reducing the impact of dynamic disorder on charge transport. On‐going modification with alkyl‐side chains will allow to tailor crystal structures for improved charge transport properties.

## Conflict of Interest

The authors declare no conflict of interest.

## Supporting information

Supporting Information

## Data Availability

The data that support the findings of this study are available in the supplementary material of this article.
