# Supplementary material for: Dinaphthotetrathienoacenes: Synthesis, Characterization, and Applications in Organic Field‐Effect Transistors
Source: Adv Sci (Weinh). 2022 Mar 16;9(19):2105674. doi: 10.1002/advs.202105674 (PMC9259716; doi:10.1002/advs.202105674)
Supplement: Supplementary file 1 — Supporting Information [file ADVS-9-2105674-s001.pdf]

Supporting Information

Dinaphthotetrathienoacenes: Synthesis, Characterization, and Applications in Organic Field-Effect Transistors

Rémy Jouclas, Jie Liu, Martina Volpi, Lygia Silva de Moraes, Guillaume Garbay, Nemo McIntosh, Marco Bardini, Vincent Lemaur, Alexandre Vercouter, Christos Gatsios, Federico Modesti, Nicholas Turetta, David Beljonne, Jérôme Cornil, Alan R. Kennedy, Norbert Koch, Peter Erk, Paolo Samorì, Guillaume Schweicher, and Yves H. Geerts**

Table of Content

1. Synthesis 3
1.1. Experimental procedures 3
1.2. ^{1}H and ^{13}C NMR spectra 8
1.3. MS spectra 15
1.4. Partial assignation of ^{1}H NMR signals of DN4T and isoDN4T 20
2. Experimental UV-vis spectra 23
3. Crystal structures and Hirshfeld Surface Analysis 24
3.1. Single-crystal X-ray diffraction (SCXRD) 24
3.2. Hirshfeld Surface Analysis 25
3.3. Variable Temperature Powder X-Ray Diffraction 27
4. Physicochemical properties 31
4.1. Thermogravimetric analysis (TGA) 31
4.2. Differential Scanning Calorimetry (DSC) 32
5. Quantum calculations 33
5.1. Methods 33
5.2. Calculation of the reorganization energy 33
5.3. Electronic couplings between dimers 34
5.4. Molecular dynamics simulations 35
5.5. Kinetic Monte Carlo simulation for hole mobility assessment on a pure hopping regime 36
6. UPS and XPS measurements 38
6.1. Determination of the ionization energy by UPS 38
6.2. Evaluation of the purity of DN4T and isoDN4T by XPS 40
7. IP measurements by PYS 43
8. Field effect transistors 43
8.1. Device fabrication and performances 43
8.2. Contact Resistance 46
8.3. Thin film morphologies 48
References 51

1. Synthesis

1.1. Experimental procedures

General

All reagents were purchased Sigma-Aldrich (now Merck), VWR, Acros, Alfa Aesar, TCI and Fluorochem and were used without further purification. Technical grade solvents were purchased from Chem-Lab and used as supplied. Anhydrous solvents as chloroform, dichloromethane, N,N-dimethylformamide and tetrahydrofurane were distilled using common methods. Air- and/or moisture-sensitive liquids and solutions were transferred via a syringe or a Teflon cannula. Analytical thin-layer chromatography (TLC) was performed on aluminum plates with 10-12 μm silica gel containing a fluorescent indicator (Merck silica gel 60 F254). TLC plates were visualized by exposure to ultraviolet light (254 nm and 365 nm). Flash column chromatography was performed on Grace Davisil LC60A (70-200μm) silica. All NMR spectra were recorded on Jeol 400 MHz spectrometer. Chemical shifts are reported in parts per million (ppm, δ scale) from tetramethylsilane for ^{1}H NMR (δ 0 ppm in chloroform and 1,1,2,2-tetrachoroethane) and from the solvent carbon for ^{13}C NMR (e.g., δ 77.16 ppm for chloroform). The data are presented in the following format: chemical shift, multiplicity (s = singlet, d = doublet, t = triplet, m = multiplet), coupling constant in hertz (Hz), signal area integration in natural numbers, assignment (italic). The Mass Spectrometry analyses have been performed in the Organic Synthesis & Mass Spectrometry Laboratory at the University of Mons (Prof Pascal Gerbaux) using MALDI-MS on a Q-TOF Premier mass spectrometer (Waters, Manchester, UK) in the positive ion mode.

2-bromo-3-methylthionaphthalene 2

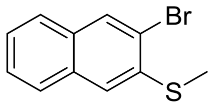

Chemical Formula: $C_{11}H_9BrS$
Exact Mass: 251,9608
Molecular Weight: 253,1570

To a solution of 2,3-dibromonaphthalene **1** (1.00 g, 3.50 mmol) in DMF (35 mL) was added sodium thiomethoxide (355 mg, 1.45 eq) and the mixture was heated at 110°C overnight. 0.65 eq of sodium thiomethoxide were added to reach completion of the reaction and the mixture was heated at the same temperature for 24h more. Then the solvent was evaporated under reduced pressure, the residue was suspended in water and the mixture was extracted 2 times with EtOAc. The combined organic layers were dried with $MgSO_4$ and evaporated under reduced pressure to give 1.229 g of crude compound. Column chromatography (pure heptane) afforded 764 g of pure **2** as a white solid with 87% yield.

^{1}H NMR (400 MHz, $CDCl_3$, δ): 8.03 (s, 1H), 7.70 (dd, *J* = 13.8, 8.1 Hz, 1H), 7.49-7.39 (m, 2H) 7.45 (s, 1H), 2.56 (s, 3H).

^{13}C NMR (101 MHz, $CDCl_3$, δ): 137.58, 132.80, 131.80, 131.23, 126.85, 126.80, 126.71, 125.86, 123.15, 120.42, 16.16.

HRMS (EI-GCMS) *m/z*: $[M]^+$ calcd for $C_{11}H_9BrS$, 251.9608; found, 251.9616.

TLC (Heptane) : Rf = 0,19.

2,5-bis-(3-methylthionaphthalen-2-yl)-thieno[3,2-b]thiophene 4

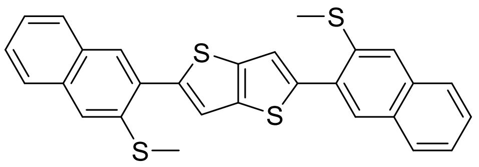

Chemical Formula: $C_{28}H_{20}S_4$
Exact Mass: 484,0448
Molecular Weight: 484,7080

2-bromo-3-methylthionaphthalene **2** (1.49 g, 5.90 mmol, 2.0 eq), 2,5-bis-trimethylstannylthieno[3,2-b]thiophene **3** (1.37 g, 2.95 mmol, 1.0 eq) and $Pd(PPh_3)_4$ (187 mg, 177 µmol, 6 mol%) were dissolved in anhydrous DMF (30 mL) under argon atmosphere and the mixture was placed at 100°C in the dark and stirred overnight. The mixture was cooled to room temperature, diluted with 30 mL of water, filtered and washed with water and methanol to give 1.35 g of pure **4** as a yellow solid with 95% yield.
^{1}H NMR (400 MHz, $CDCl_3$, δ): 7.92 (s, 2H), 7.80 (dd, *J* = 10.7, 8.0 Hz, 3H), 7.63 (s, 2H), 7.55 (s, 2H), 7.80 (dd, *J* = 10.7, 8.2 Hz, 1H), 7.45 (t, *J* = 6.9 Hz, 1H), 2.56 (s, 6H).
^{13}C NMR (101 MHz, $CDCl_3$, δ): 142.46, 139.65, 136.62, 133.58, 131.82, 130.93, 130.33, 128.04, 127.22, 126.65, 125.87, 123.28, 120.37, 16.31.
HRMS (MALDI) *m/z*: $[M]^+$ calcd for $C_{28}H_{20}S_4$, 484.0448; found, 484.0455.
TLC (Heptane / $CHCl_3$ 1:1) : Rf = 0,50.

2,5-bis-(3-methylsulfinylnaphthalen-2-yl)-thieno[3,2-b]thiophene 5

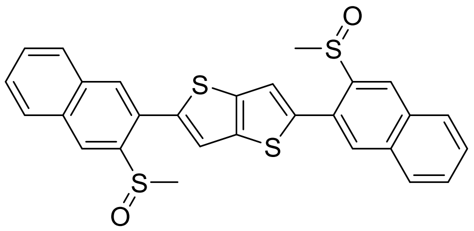

Chemical Formula: $C_{28}H_{20}O_2S_4$
Exact Mass: 516,0346
Molecular Weight: 516,7060

To a solution of 2,5-bis-(3-methylthionaphthalen-2-yl)-thieno[3,2-b]thiophene **4** (1.09 g, 2.26 mmol) in chloroform (225 mL) at 0°C was added m-CPBA (1.04 g, 75% w/w in water, 4.51 mmol, 2.0 eq) and the mixture was stirred at 0°C for 1h. Then the mixture was washed 3 times with an aqueous solution of Na_2CO_3 (10% w/w), dried with $MgSO_4$ and evaporated under reduced pressure to afford 1.33 g of crude compound that was used in the next step without further purification.
^{1}H NMR (400 MHz, $CDCl_3$, δ): 8.64 (s, 2H), 8.08-8.03 (m, 2H), 8.02 (d, *J*=3.3 Hz, 2H), 7.97-7.91 (m, 2H), 7.67-7.62 (m, 4H), 7.52 (d, *J*=1.2 Hz, 2H), 2.61 (d, *J*=4.7 Hz, 6H).
^{13}C NMR (101 MHz, $CDCl_3$, δ): 142.13 and 142.11 (2 enantiomer couples), 141.26 and 141.17 (2 enantiomer couples), 140.39 and 140.37 (2 enantiomer couples), 134.09, 133.02, 131.07 and 131.04 (2 enantiomer couples), 128.71, 128.56, 128.16 and 128.15 (2 enantiomer couples), 128.08 and 128.04 (2 enantiomer couples), 128.04 and 128.00 (2 enantiomer couples), 125.05, 120.02 and 120.01 (2 enantiomer couples), 42.49 and 42.46 (2 enantiomer couples).
HRMS (MALDI) *m/z*: $[M^{\cdot}-CH_3^{\cdot}]^+$ calcd for $C_{27}H_{17}O_2S_4$, 501.0111; found, 501.0120.
TLC (Toluene / Ethanol 95:5) : Rf = 0,17.

DN4T (naphtho[2,3-b]thieno-[2''',3''':4'',5'']thieno[2'',3'':4',5']thieno[3',2'-b]naphtho[2,3-b]thiophene)

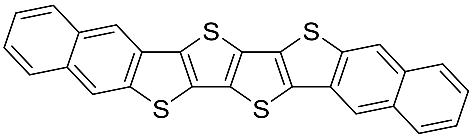

Chemical Formula: $C_{26}H_{12}S_4$
Exact Mass: 451,9822
Molecular Weight: 452,6220

To a solution of phosphorus pentoxide (167 mg, 1.25 mmol, 0.5 eq) in trifluoromethanesulfonic acid (5 mL) at 0°C was added 2,5-bis-(3-methylsulfinylnaphthalen-2-yl)-thieno[3,2-b]thiophene **5** (1.29 g, 2.5 mmol, 1 eq) and the mixture was stirred under argon atmosphere at room temperature for 60h. Then the mixture was added dropwise to an ice/water mixture under strong stirring, the resulting precipitate was filtered, washed with water and refluxed in pyridine (40 mL) overnight. The mixture was cooled to room temperature and diluted with water (40 mL), the precipitate was filtered, washed with water and methanol and dried under vacuum. The resulting residue was purified by thermal sublimation at 380°C and 10^{-10} Pa to afford 468 mg of pure compound as a strong yellow solid with 41% yield.
^{1}H NMR (600 MHz, $C_2D_2Cl_4$, δ): 8.42 (s, 2H), 8.38 (s, 2H), 8.08 (s, 2H), 7.98 (s, 2H), 7.59 (s, 4H).
HRMS (MALDI) *m/z*: $[M]^+$ calcd for $C_{26}H_{12}S_4$, 451.9822; found, 451.9821.

1-methylthio-2-naphthol 7[1]

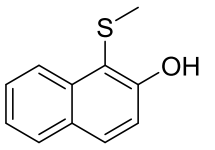

Chemical Formula: $C_{11}H_{10}OS$
Exact Mass: 190,0452
Molecular Weight: 190,2600

To a solution of β-naphthol **6** (3.00 g, 20.8 mmol) and sodium methylsulfinate (5.31 g, 52.0 mmol, 2.5 eq) in water (10 mL) were successively added formic acid (10 mL) and diiodine (11.1 g, 43.7 mmol, 2.1 eq) and the mixture was heated at 110°C overnight. The reaction was quenched by the addition of an aqueous solution of $Na_2S_2O_3$ (50 mL, 10% w/w), the mixture was extracted 2 times with chloroform. The combined organic layers were dried over $MgSO_4$ and evaporated under reduced pressure to give 4.15 g of crude compound that was purified by column chromatography (heptane/chloroform 9:1) to afford 3.53 g of pure **7** as a yellow oil with 89% yield.
^{1}H NMR (400 MHz, $CDCl_3$, δ): 8.35 (d, J = 8.5 Hz, 1H), 7.80 (d, J = 8.9 Hz, 2H), 7.59 (t, J = 7.8 Hz, 1H), 7.42 – 7.35 (m, 2H), 7.28 (dd, *J* = 8.9, 0.9 Hz, 1H), 2.29 (s, 3H).

1-methylthio-2-trifluoromethanesulfonylnaphthalene 8

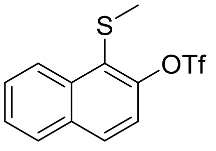

Chemical Formula: $C_{12}H_9F_3O_3S_2$
Exact Mass: 321,9945
Molecular Weight: 322,3162

To a solution of 1-methylthio-2-naphthol **7** (3.53 g, 18.6 mmol) in DCM (90 mL) at 0°C were successively added pyridine (4.73 mL, 58.4 mmol, 3.15 eq) and trifluoromethanesulfonic anhydride (3.60 mL, 21.3 mmol, 1.15 eq) under argon atmosphere and the mixture was stirred at room temperature overnight. The reaction was quenched by the addition of an aqueous solution of HCl (90 mL, 1M), the phases were separated, and the aqueous layer was extracted with DCM. The combined organic layers were dried with $MgSO_4$ and evaporated under reduced pressure to afford 6.51 g of crude product that was purified by column chromatography (heptane/DCM 9:1) to afford 5.64 g of pure **8** as a white solid with 95% yield.
^{1}H NMR (400 MHz, $CDCl_3$, δ): 8.65 (d, J = 8.5 Hz, 1H), 7.92 (d, J = 9.2 Hz, 2H), 7.71 (ddd, J = 8.4, 6.8, 1.3 Hz, 1H), 7.61 (ddd, J = 8.1, 6.9, 1.2 Hz, 1H), 7.39 (d, J = 9.0 Hz, 1H), 2.45 (s, 3H).

^{13}C NMR (101 MHz, $CDCl_3$, δ): 149.60, 135.09, 133.09, 131.46, 128.88, 128.45, 128.19, 127.41, 126.98, 119.61, 118.92 (q, *J* = 320.3 Hz), 19.34.
HRMS (EI-GCMS) *m/z*: $[M]^+$ calcd for $C_{12}H_9F_3O_3S_2$, 321.9945; found, 321.9944.
TLC (Petroleum Ether / DCM 9:1) : Rf = 0,29.

2,5-bis-(1-methylthionaphthalen-2-yl)-thieno[3,2-b]thiophene 9

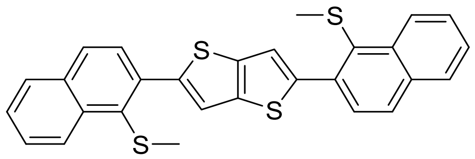

Chemical Formula: $C_{28}H_{20}S_4$
Exact Mass: 484,0448
Molecular Weight: 484,7080

Lithium chloride (455 mg, 10.7 mmol, 10 eq) was flame-dried under vacuum and dissolved in dry DMF (23 mL) under argon atmosphere. The resulting solution was added to a mixture of 1-methylthio-2-trifluoromethanesulfonylnaphthalene **8** (726 mg, 2.25 mmol, 2.1 eq), $Pd(PPh_3)_4$ (113 mg, 107 mmol, 10 mol%), copper iodide (41 mg, 217 µmol, 20 mol%) and 2,5-bis-trimethylstannylthieno[3,2-b]thiophene **3** (500 mg, 10.7 mmol, 1 eq) and was placed at 110°C under argon atmosphere and stirred overnight. The mixture was cooled to room temperature, the reaction was quenched by the addition of an aqueous solution of KF (23 mL, 10% w/w), and the precipitate was filtered and washed with water. The crude product was purified by Soxhlet with methanol to afford 485 mg of pure compound as a strong yellow solid with 93% yield.
^1H NMR (400 MHz, $CDCl_3$, δ): 8.81 (d, *J* = 8.8 Hz, 2H), 7.88 (dd, *J* = 8.3, 4.9 Hz, 4H), 7.72 (d, *J* = 8.5 Hz, 2H), 7.66 (ddd, *J* = 8.4, 6.8, 1.4 Hz, 2H), 7.60 (s, 2H), 7.55 (ddd, *J* = 8.0, 6.8, 1.0 Hz, 2H), 2.34 (s, 6H).

^{13}C NMR (101 MHz, $CDCl_3$, δ): 145.06, 140.33, 137.59, 135.22, 133.70, 132.60, 129.21, 128.75, 128.65, 127.65, 127.36, 126.67, 120.47, 29.86.
HRMS (MALDI) *m/z*: $[M]^+$ calcd for $C_{28}H_{20}S_4$, 484.0448; found, 484.0448.
TLC (Heptane / $CHCl_3$ 1:1) : Rf = 0,68

2,5-bis-(1-methylsulfinylnaphthalen-2-yl)-thieno[3,2-b]thiophene 10

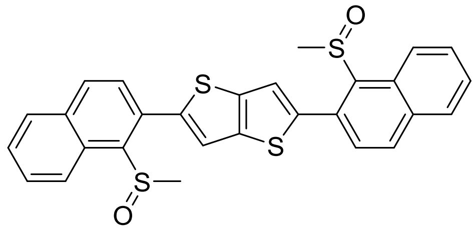

Chemical Formula: $C_{28}H_{20}O_2S_4$
Exact Mass: 516,0346
Molecular Weight: 516,7060

To a solution of 2,5-bis-(1-methylthionaphthalen-2-yl)-thieno[3,2-b]thiophene **9** (1.93 g, 3.98 mmol) in chloroform (400 mL) was added m-CPBA (1.83 mg, 75% w/w in water, 2.0 eq) at 0°C and the mixture was stirred at this temperature for 1h30. Then the mixture was washed 3 times with an aqueous solution of Na_2CO_3 (10% w/w), dried with $MgSO_4$ and evaporated under reduced pressure to afford 2.25 g of crude compound that was used in the next step without further purification.
^1H NMR (400 MHz, $CDCl_3$, δ): 9.45 (d, *J* = 8.6 Hz, 2H), 7.99 (d, *J* = 8.5 Hz, 2H), 7.98 (d, *J* = 8.1 Hz, 2H), 7.79 – 7.62 (m, 4H), 7.56 (d, *J* = 8.4 Hz, 2H), 7.31 (d, *J* = 3.8 Hz, 2H), 3.18 (d, *J* = 1.1 Hz, 6H).
^{13}C NMR (101 MHz, $CDCl_3$, δ): 141.59, 140.24 and 140.22 (2 enantiomer couples), 138.21 and 138.12 (2 enantiomer couples), 134.68 and 134.65 (2 enantiomer couples), 133.20, 132.17 and 132.15

(2 enantiomer couples), 130.53 and 130.51 (2 enantiomer couples), 129.53, 128.40 and 128.36 (2 enantiomer couples), 128.20, 127.65, 124.56, 120.88 and 120.84 (2 enantiomer couples), 40.38.
HRMS (MALDI) *m/z*: $[M^{\cdot}-CH_3^{\cdot}]^+$ calcd for $C_{27}H_{17}O_2S_4$, 501.0111; found, 501.0141.
TLC (Toluene / Ethanol 95:5) : Rf = 0.25.

isoDN4T (naphtho[1,2-b]thieno-[2''',3''':4'',5'']thieno[2'',3'':4',5']thieno[3',2'-b]naphtho[1,2-b]thiophene)

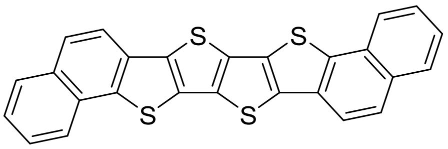

Chemical Formula: $C_{26}H_{12}S_4$
Exact Mass: 451,9822
Molecular Weight: 452,6220

To a solution of phosphorus pentoxide (192 mg, 1.31 mmol, 0.5 eq) in trifluoromethanesulfonic acid (5.4 mL) at 0°C was added 2,5-bis-(1-methylsulfinylnaphthalen-2-yl)-thieno[3,2-b]thiophene **10** (1.35 g, 2.62 mmol, 1 eq) and the mixture was stirred under argon atmosphere at room temperature for 60h. Then the mixture was added dropwise to an ice/water mixture under strong stirring, the resulting precipitate was filtered, washed with water and refluxed in pyridine (40 mL) overnight. The mixture was cooled to room temperature and diluted with water (40 mL), the precipitate was filtered, washed with water and methanol and dried under vacuum. The resulting residue was purified by thermal sublimation at 380°C and 10^{-10} Pa to afford 770 mg of pure compound as a strong yellow solid with 65% yield.
^{1}H NMR (400 MHz, $C_2D_2Cl_4$, δ): 8.18 (d, *J* = 8.2 Hz, 2H), 8.03 (d, *J* = 8.0 Hz, 2H), 7.93 (d, *J* = 2.7 Hz, 4H), 7.69 (t, *J* = 7.6 Hz, 2H), 7.61 (t, *J* = 7.5 Hz, 2H).
HRMS (MALDI) *m/z*: $[M]^+$ calcd for $C_{26}H_{12}S_4$, 451.9822; found, 451.9814.

1.2. ^{1}H and ^{13}C NMR spectra

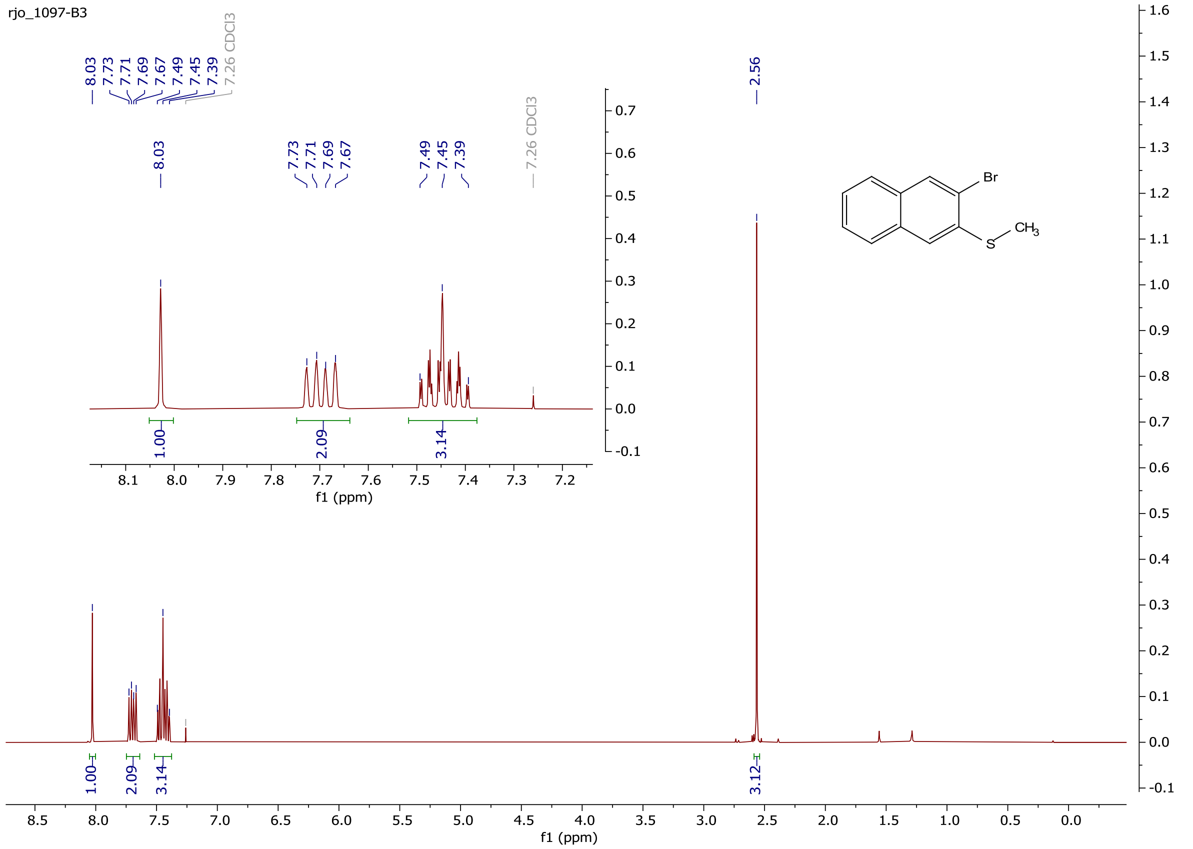

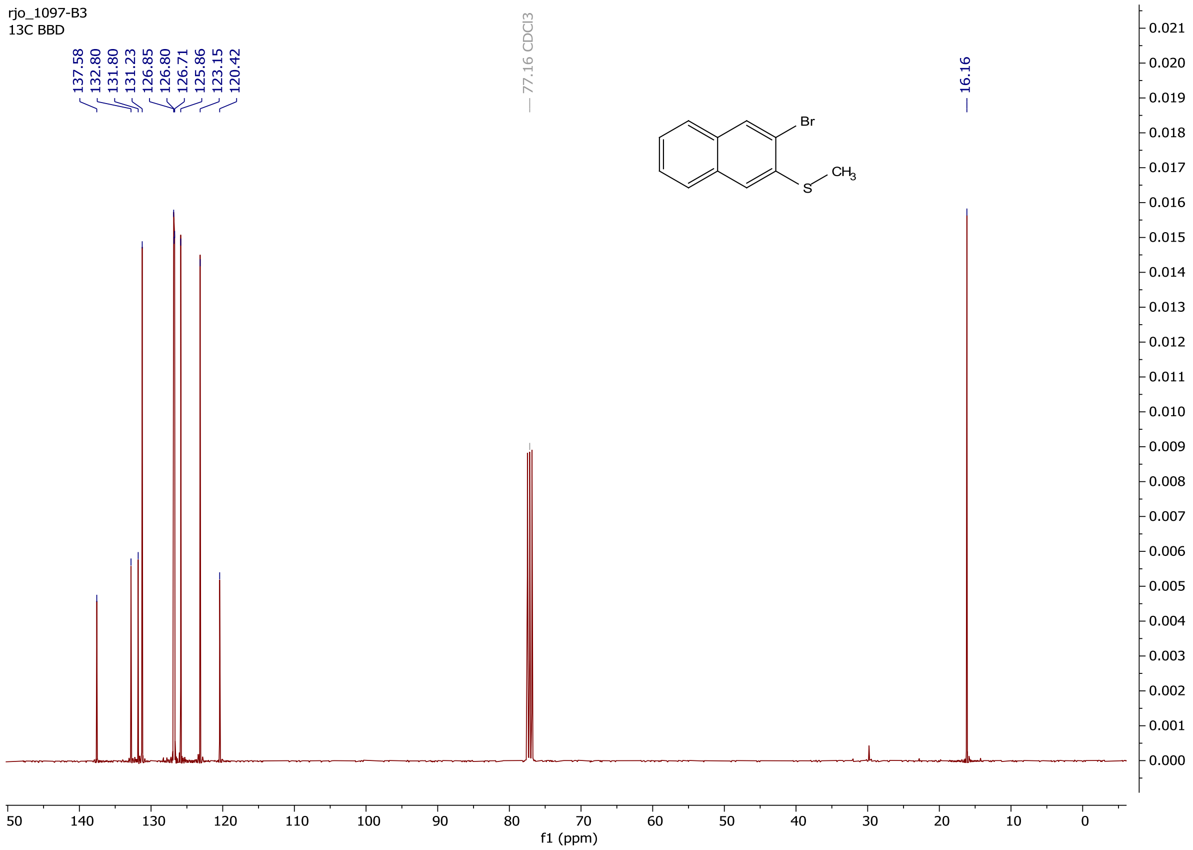

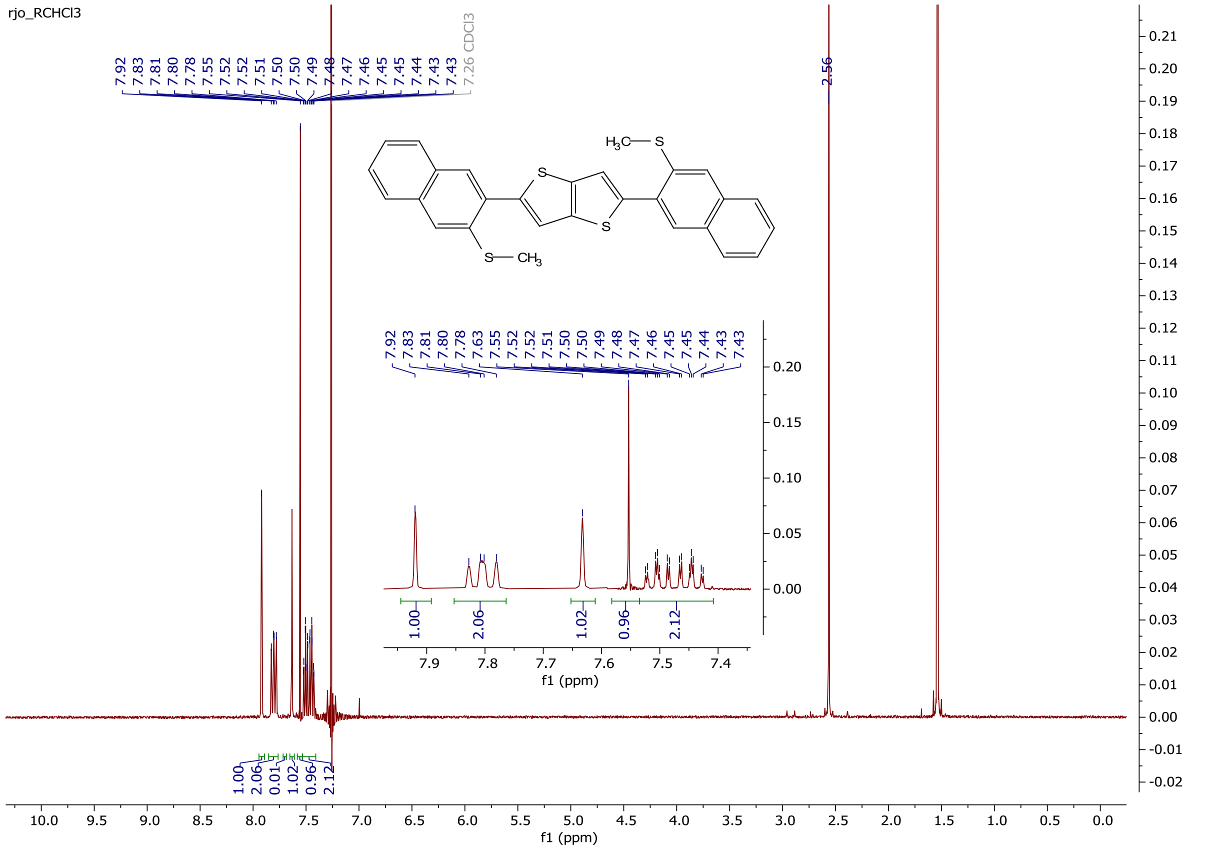
rjo_RCHCl3
7.92
7.83
7.81
7.80
7.78
7.55
7.52
7.52
7.51
7.50
7.50
7.49
7.48
7.47
7.46
7.45
7.45
7.44
7.43
7.43
7.26 CDCl3
2.56
H_3C—S
S
S
S— CH_3
7.92
7.83
7.81
7.80
7.78
7.63
7.55
7.52
7.52
7.51
7.50
7.50
7.49
7.48
7.47
7.46
7.45
7.45
7.44
7.43
7.43
1.00
2.06
1.02
0.96
2.12
f1 (ppm)
1.00
2.06
0.01
1.02
0.96
2.12
f1 (ppm)

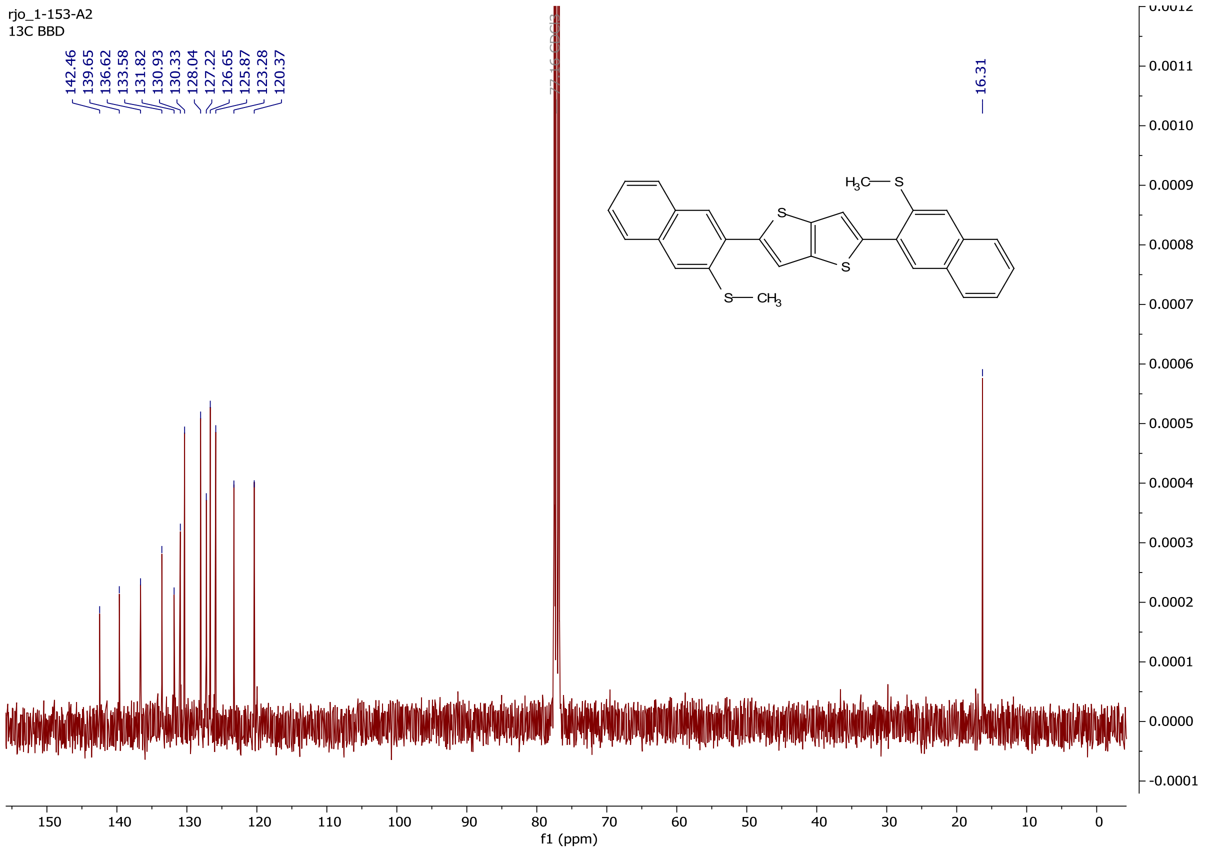
rjo_1-153-A2
13C BBD
142.46
139.65
136.62
133.58
131.82
130.93
130.33
128.04
127.22
126.65
125.87
123.28
120.37
16.31
H_3C—S
S
S
S— CH_3
f1 (ppm)

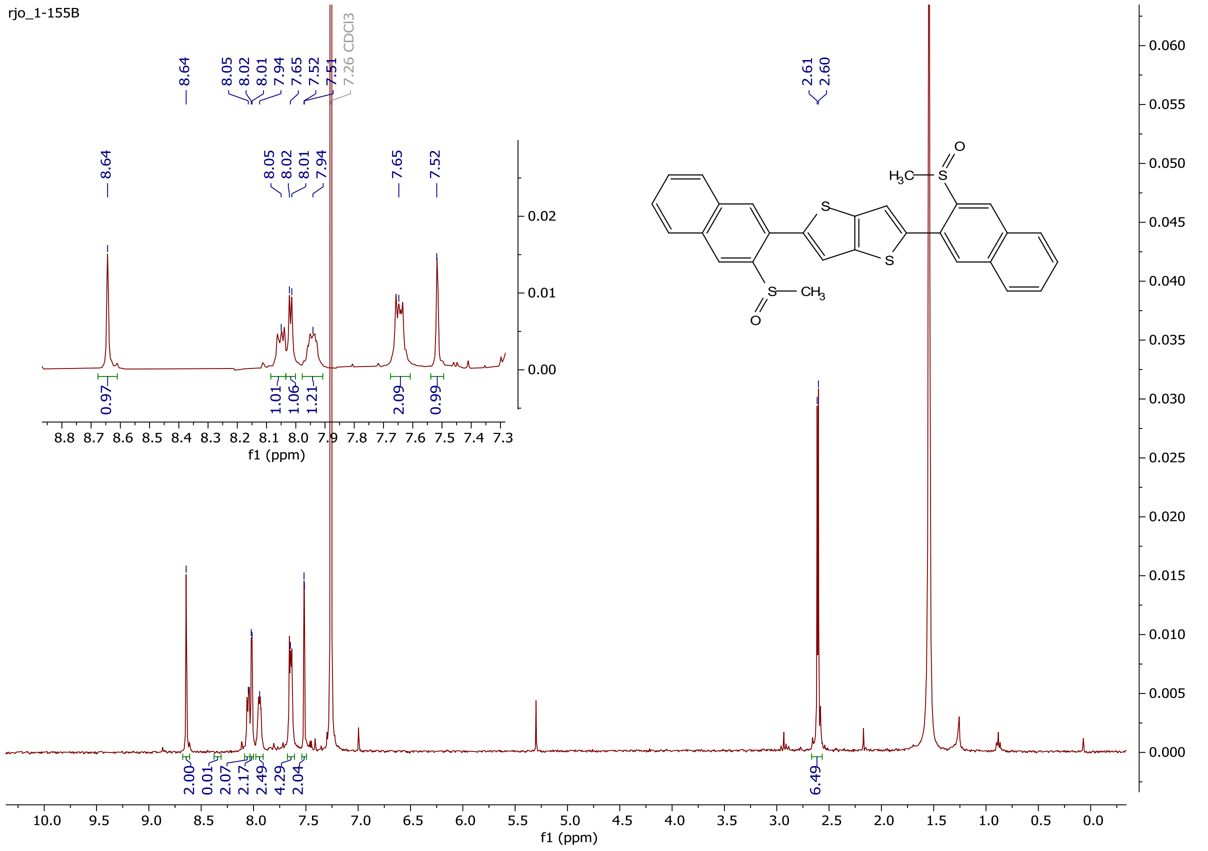
rjo_1-155B
8.64
8.05
8.02
8.01
7.94
7.65
7.52
7.51
7.26 CDCl3
2.61
2.60
0.97
1.01
1.06
1.21
2.09
0.99
2.00
0.01
2.07
2.17
2.49
4.29
2.04
6.49
S
S
H_3C
O
CH_3
f1 (ppm)

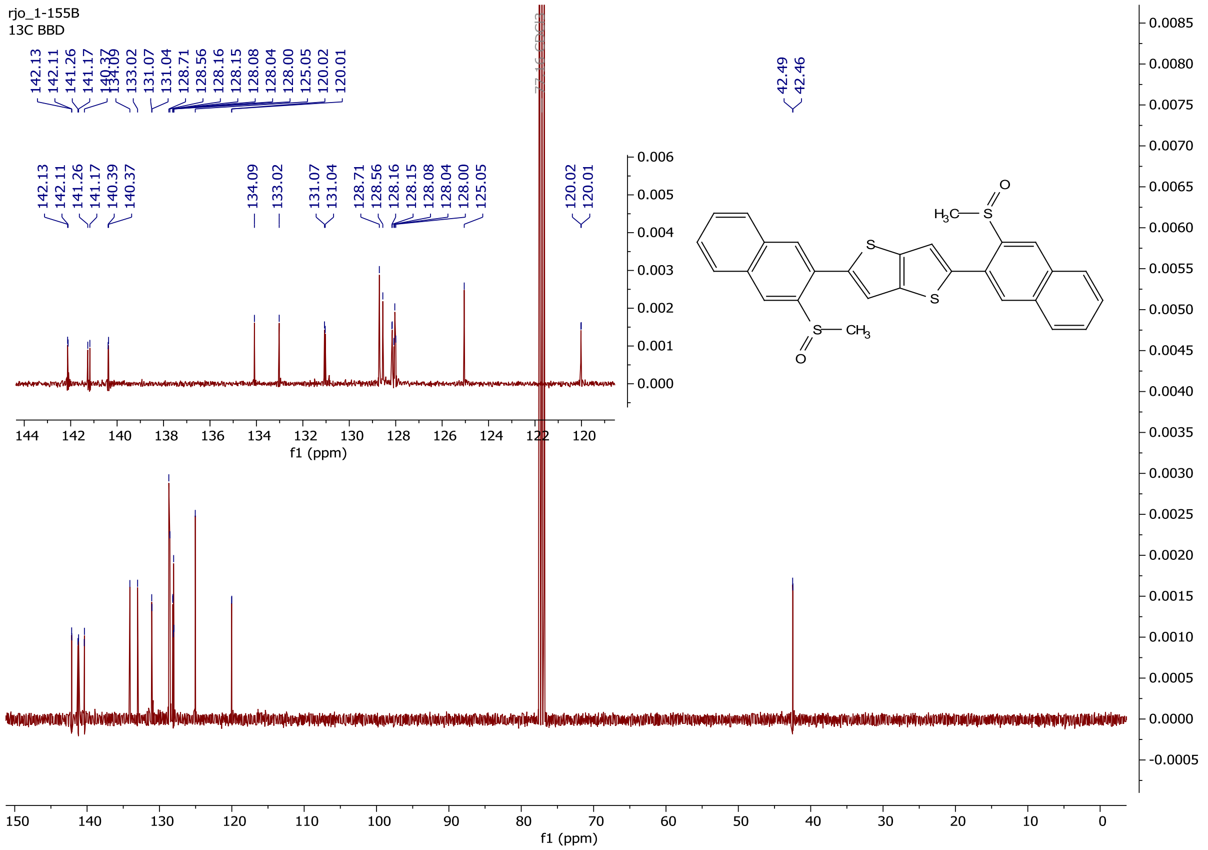
rjo_1-155B
13C BBD
142.13
142.11
141.26
141.17
140.39
140.37
134.09
133.02
131.07
131.04
128.71
128.56
128.16
128.15
128.08
128.04
128.00
125.05
120.02
120.01
42.49
42.46
S
S
H_3C
O
CH_3
f1 (ppm)

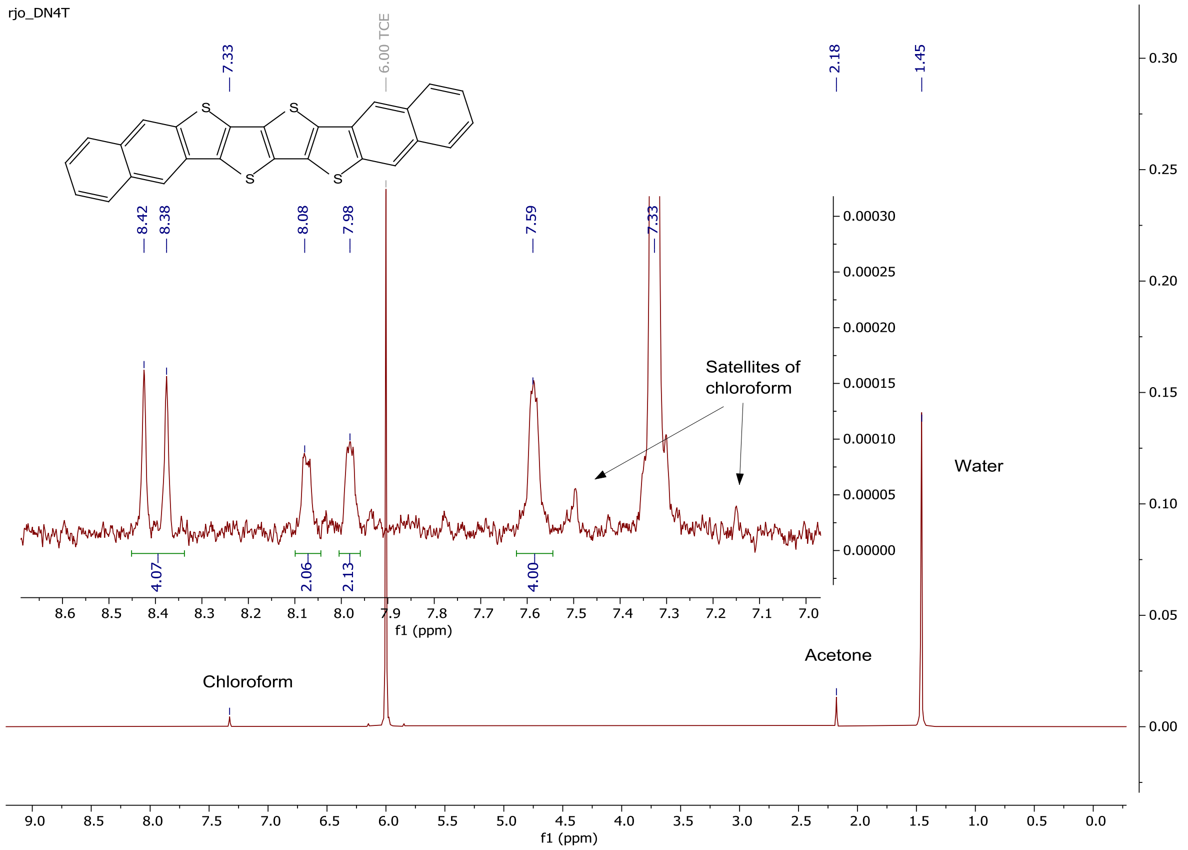
rjo_DN4T
7.33
6.00 TCE
2.18
1.45
8.42
8.38
8.08
7.98
7.59
7.33
Satellites of chloroform
Water
4.07
2.06
2.13
4.00
f1 (ppm)
Acetone
Chloroform
f1 (ppm)

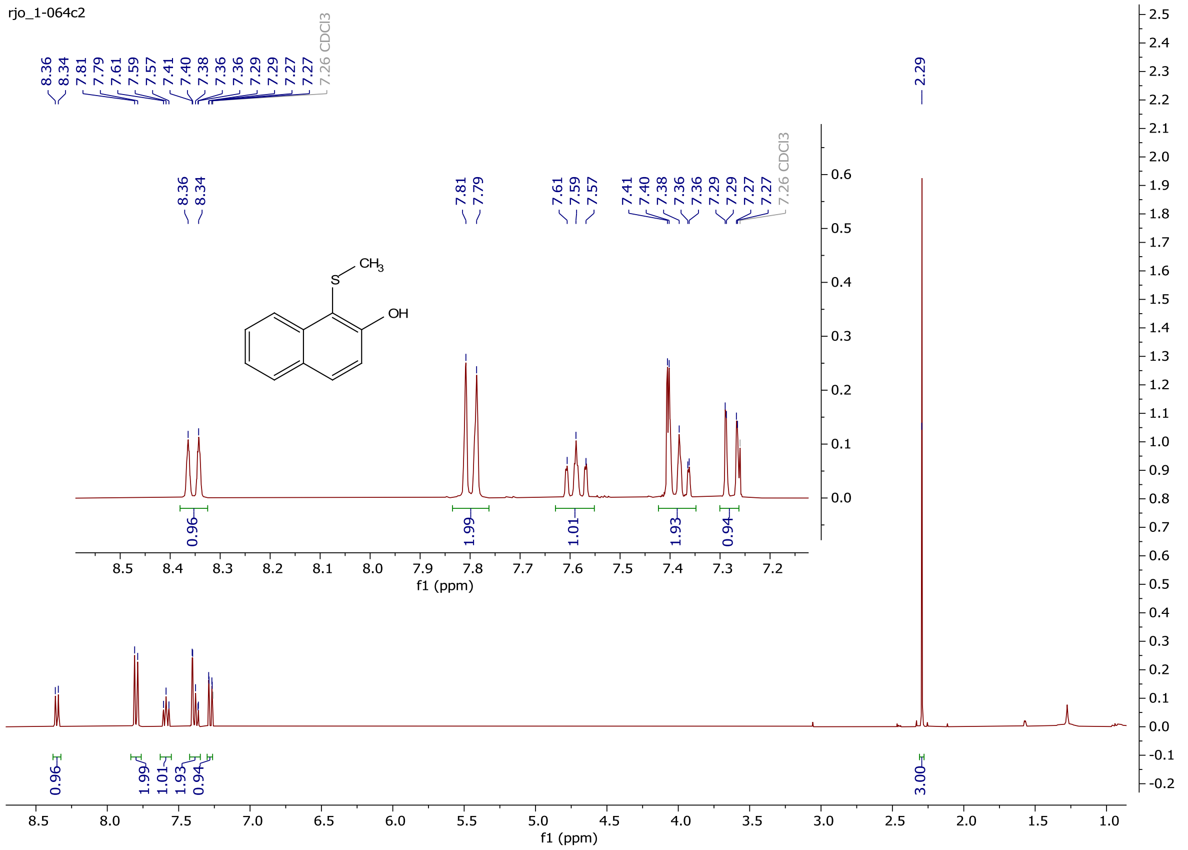
rjo_1-064c2
8.36
8.34
7.81
7.79
7.61
7.59
7.57
7.41
7.40
7.38
7.36
7.36
7.29
7.29
7.27
7.27
7.26 CDCl3
2.29
CH3
OH
0.96
1.99
1.01
1.93
0.94
3.00
f1 (ppm)
f1 (ppm)

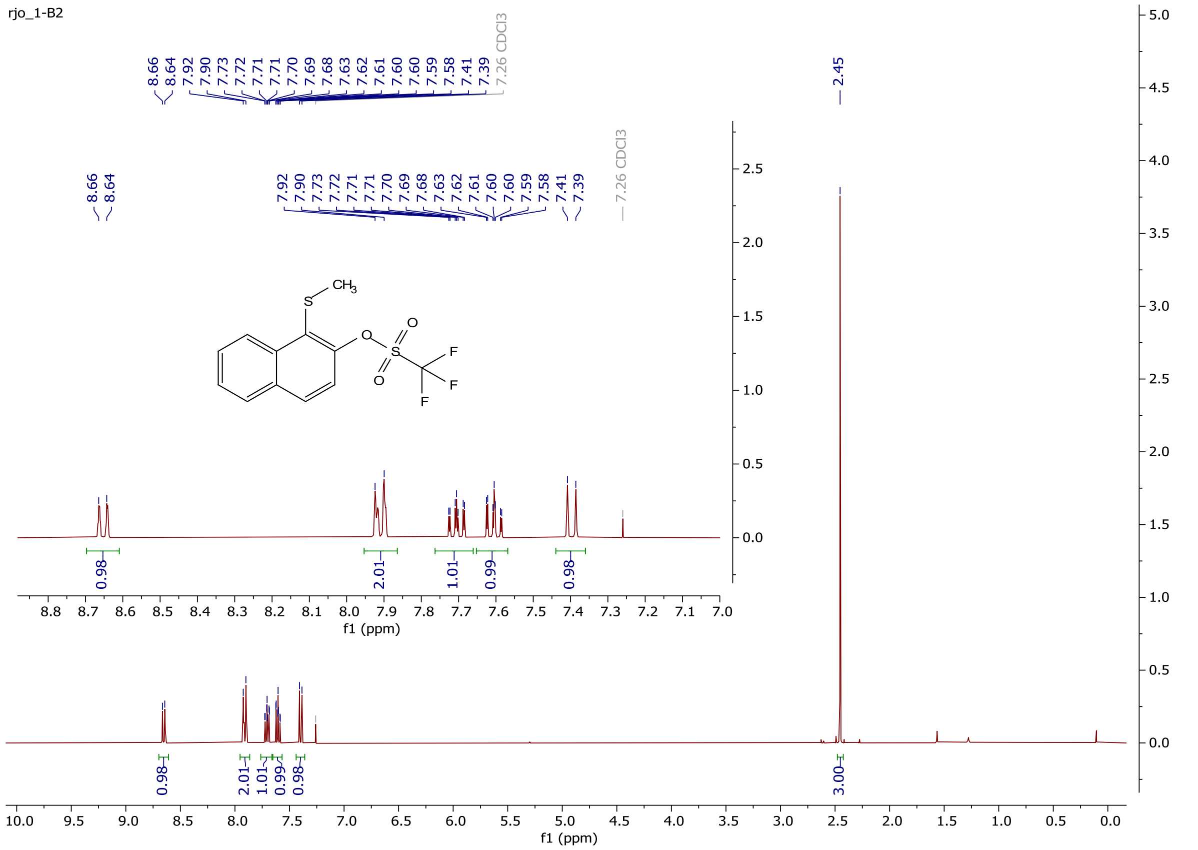
rjo_1-B2
8.66 8.64 7.92 7.90 7.73 7.72 7.71 7.71 7.70 7.69 7.68 7.63 7.62 7.61 7.60 7.60 7.59 7.58 7.41 7.39
7.26 CDCl3
2.45
0.98 2.01 1.01 0.99 0.98 3.00
f1 (ppm)

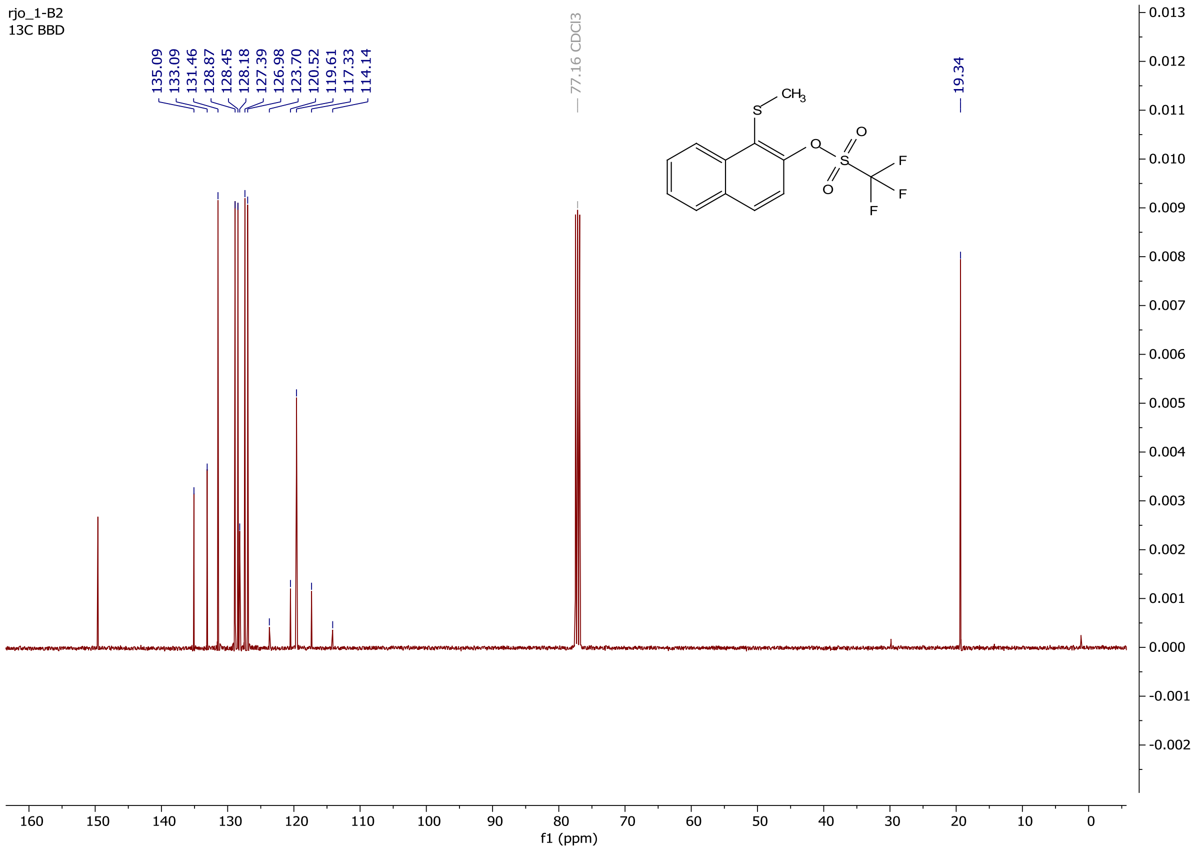
rjo_1-B2
13C BBD
135.09 133.09 131.46 128.87 128.45 128.18 127.39 126.98 123.70 120.52 119.61 117.33 114.14
77.16 CDCl3
19.34
f1 (ppm)

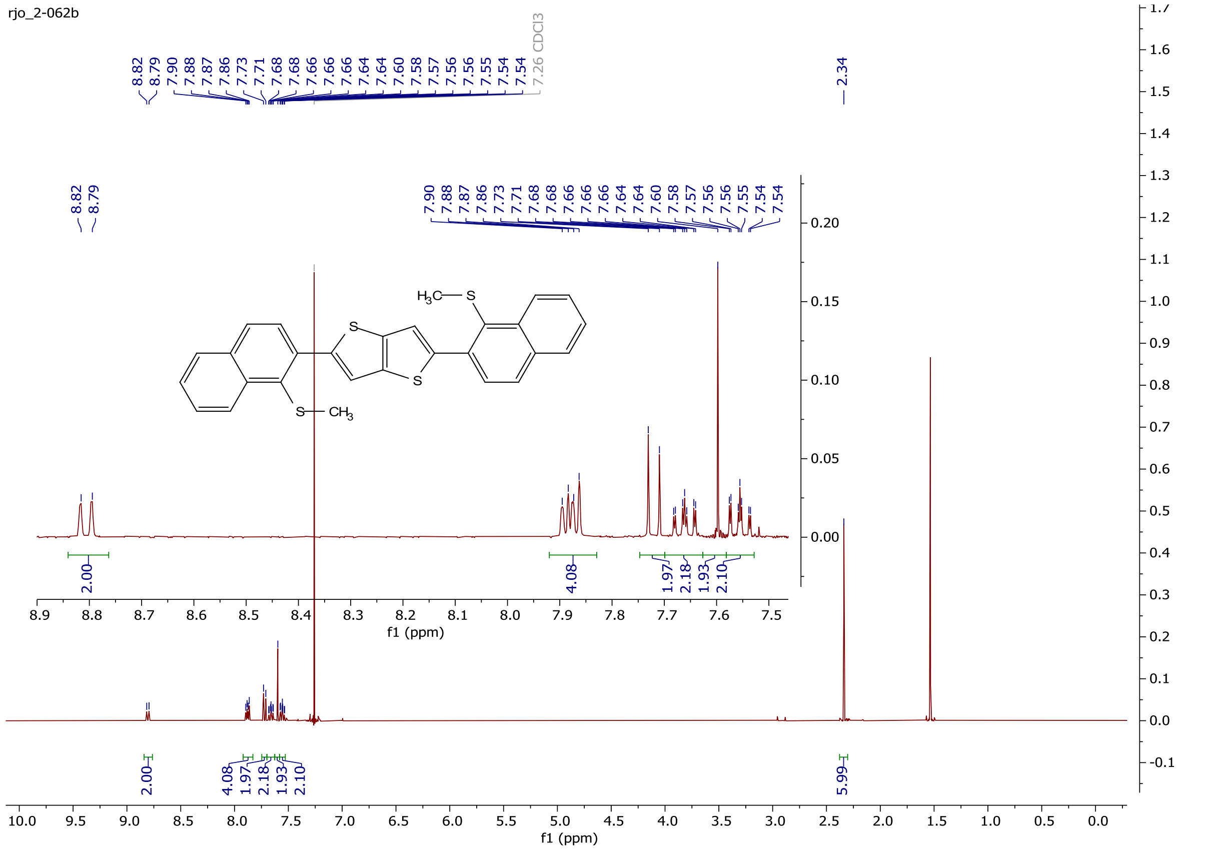
rjo_2-062b
8.82
8.79
7.90
7.88
7.87
7.86
7.73
7.71
7.68
7.68
7.66
7.66
7.66
7.64
7.64
7.60
7.58
7.57
7.56
7.56
7.55
7.54
7.54
7.26 CDCl3
2.34
H3C—S
S
S
S—CH3
2.00
4.08
1.97
2.18
1.93
2.10
5.99
f1 (ppm)

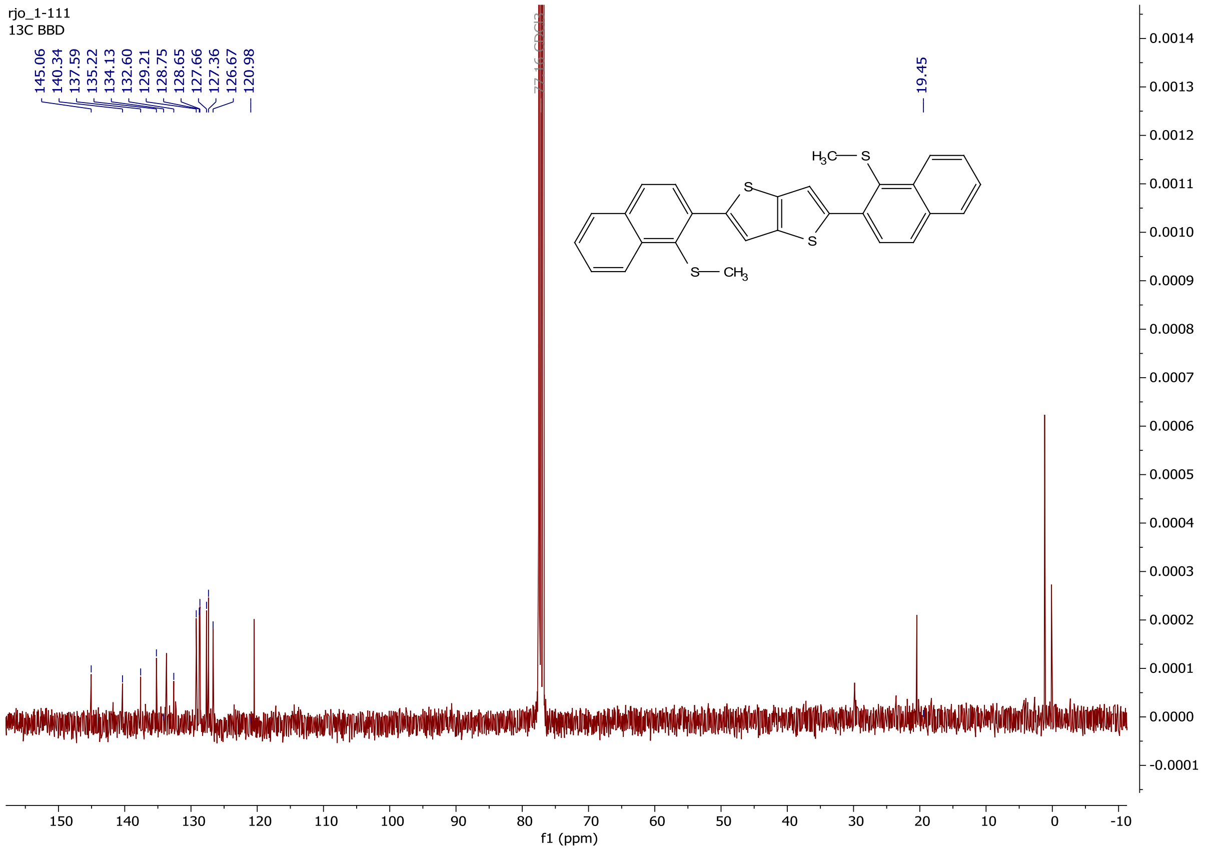
rjo_1-111
13C BBD
145.06
140.34
137.59
135.22
134.13
132.60
129.21
128.75
128.65
127.66
127.36
126.67
120.98
19.45
H3C—S
S
S
S—CH3
f1 (ppm)

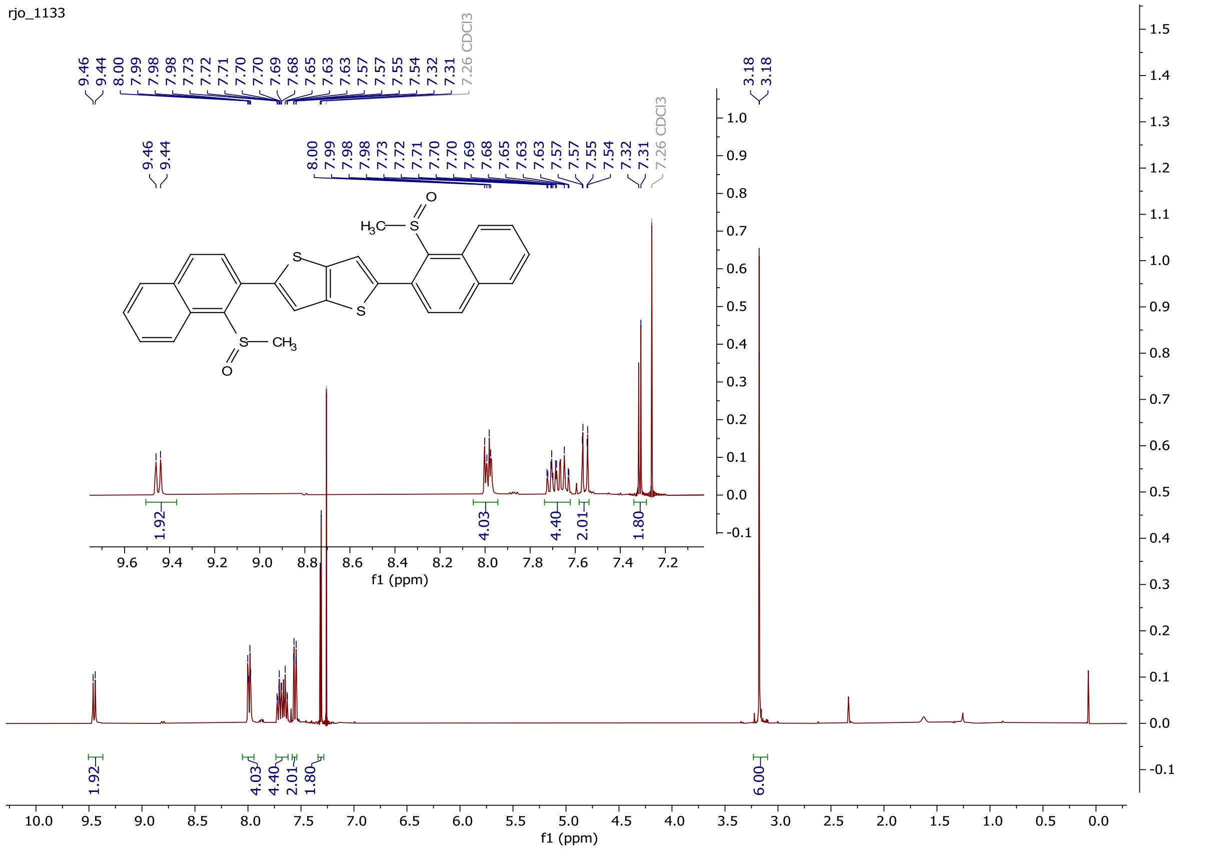
rjo_1133
9.46
9.44
8.00
7.99
7.98
7.98
7.73
7.72
7.71
7.70
7.70
7.69
7.68
7.65
7.63
7.63
7.57
7.57
7.55
7.54
7.32
7.31
7.26 CDCl3
3.18
3.18
1.92
4.03
4.40
2.01
1.80
6.00
f1 (ppm)

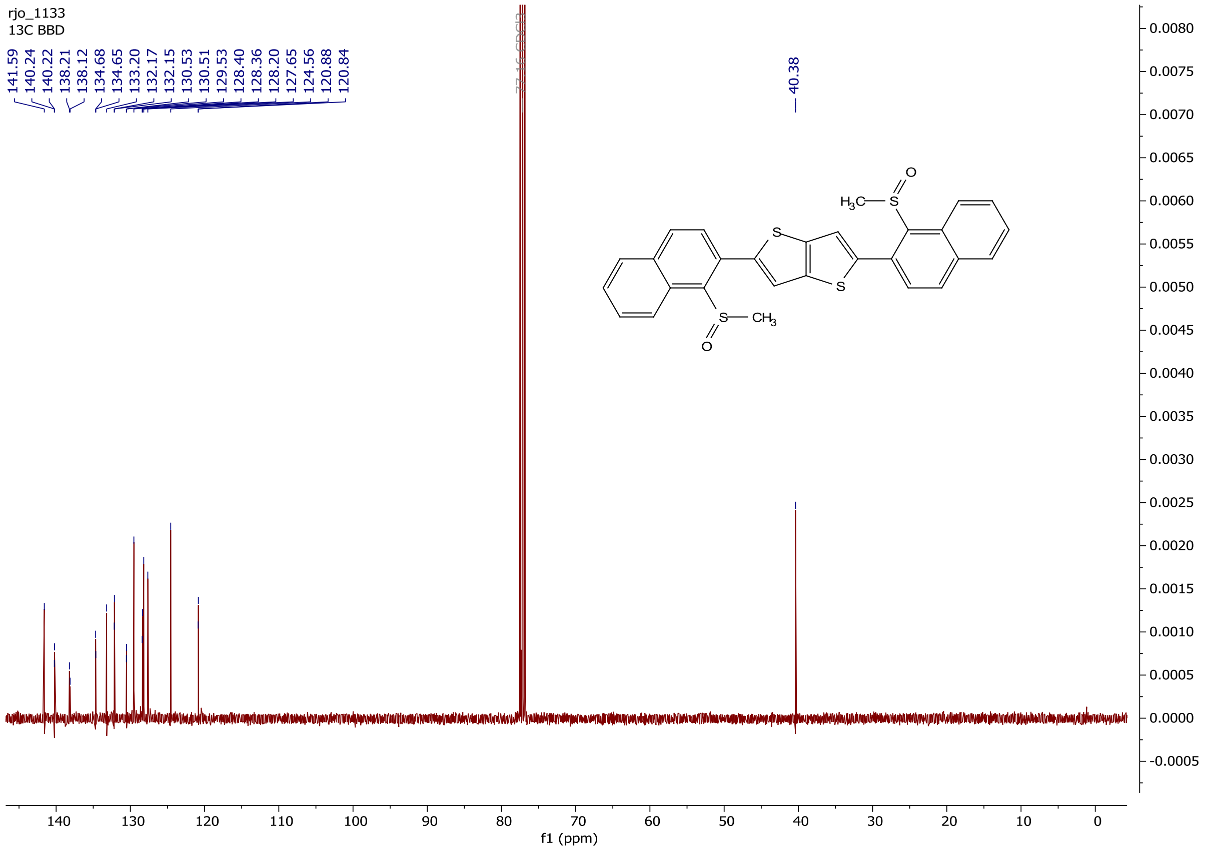
rjo_1133
13C BBD
141.59
140.24
140.22
138.21
138.12
134.68
134.65
133.20
132.17
132.15
130.53
130.51
129.53
128.40
128.36
128.20
127.65
124.56
120.88
120.84
40.38
f1 (ppm)

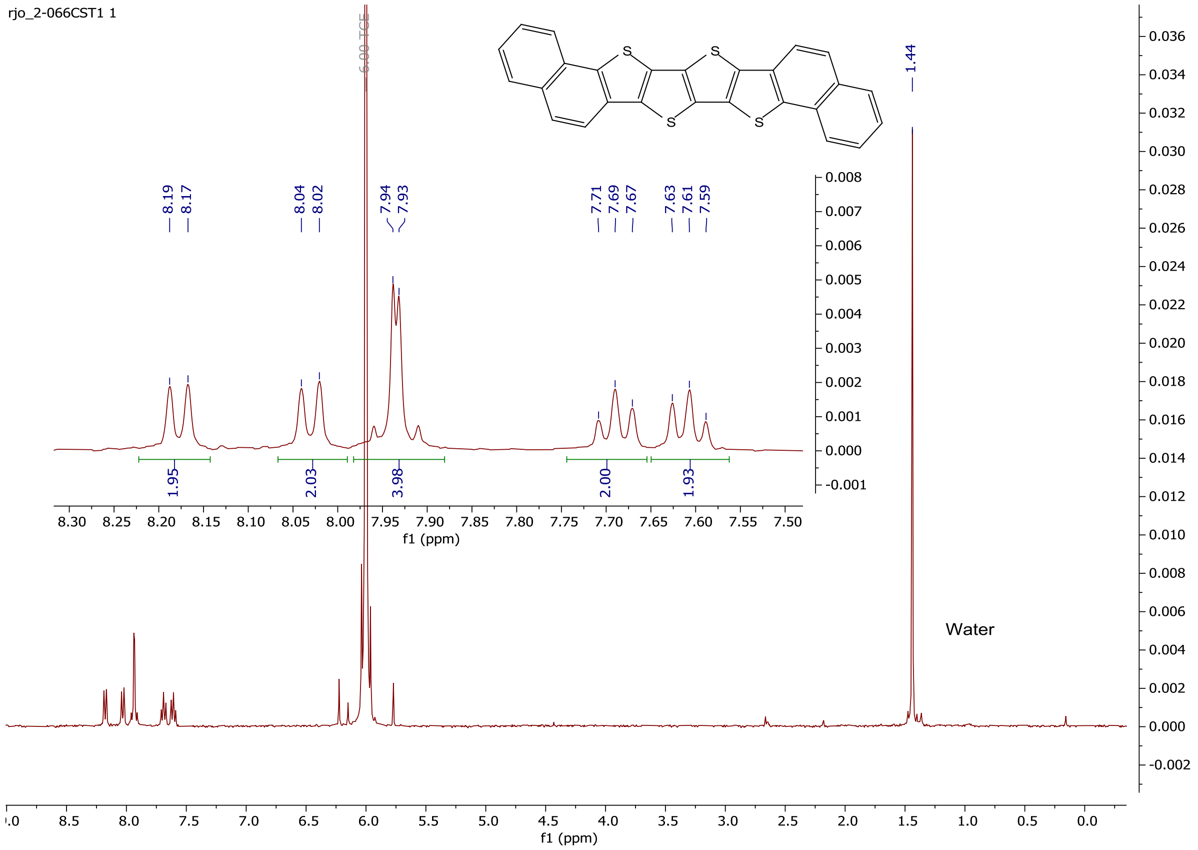

1.3. MS spectra

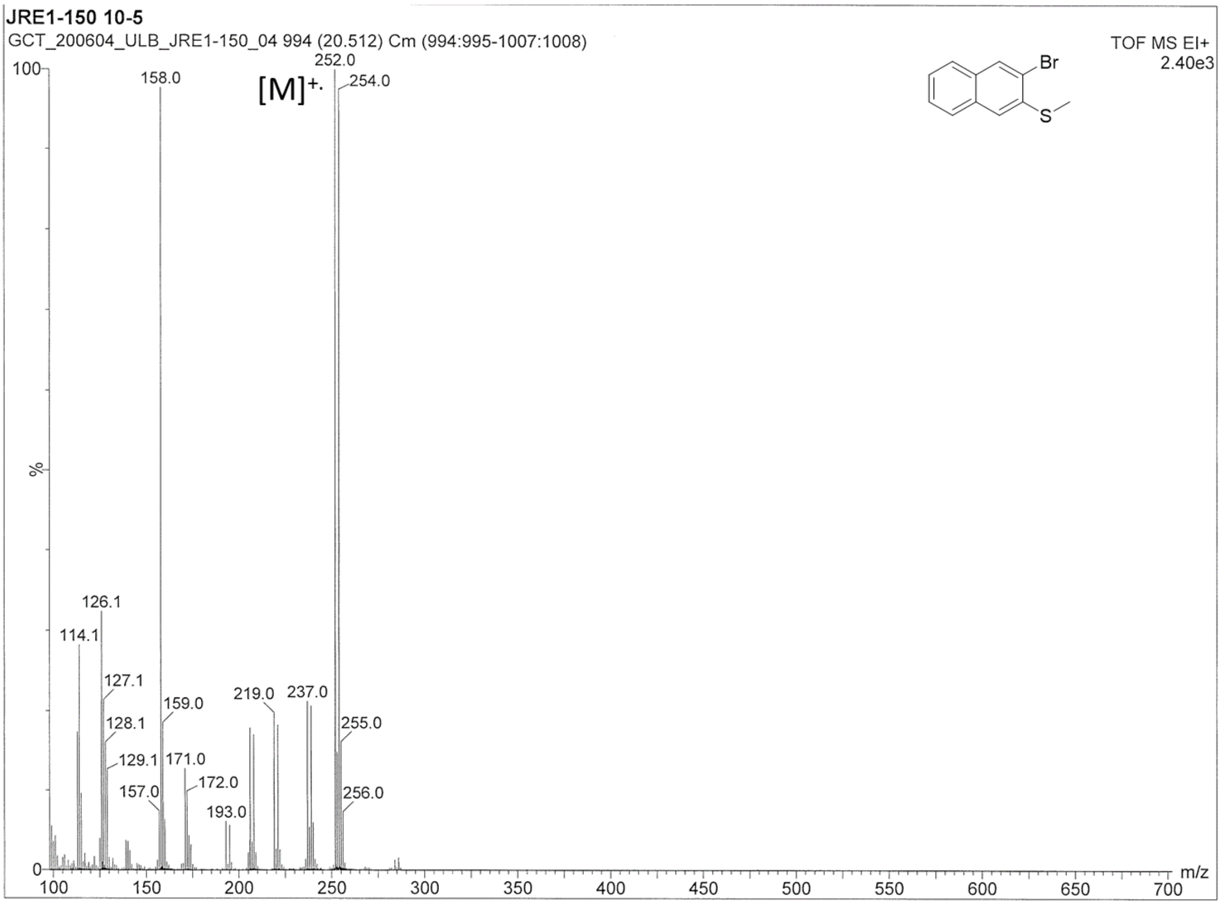

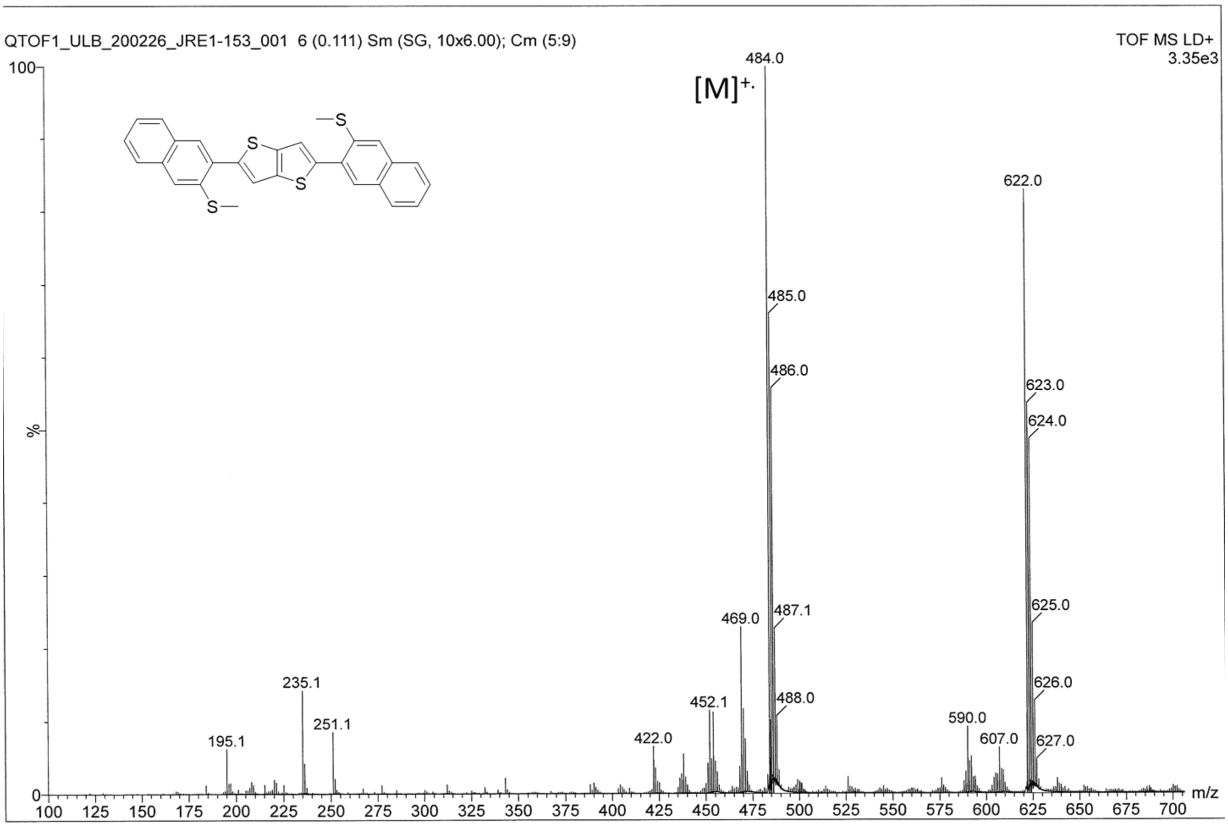
QTOF1_ULB_200226_JRE1-153_001 6 (0.111) Sm (SG, 10x6.00); Cm (5:9)
TOF MS LD+
3.35e3
[M]+·
484.0
485.0
486.0
622.0
623.0
624.0
625.0
626.0
627.0
469.0
487.1
488.0
452.1
422.0
235.1
251.1
195.1
590.0
607.0
%
m/z

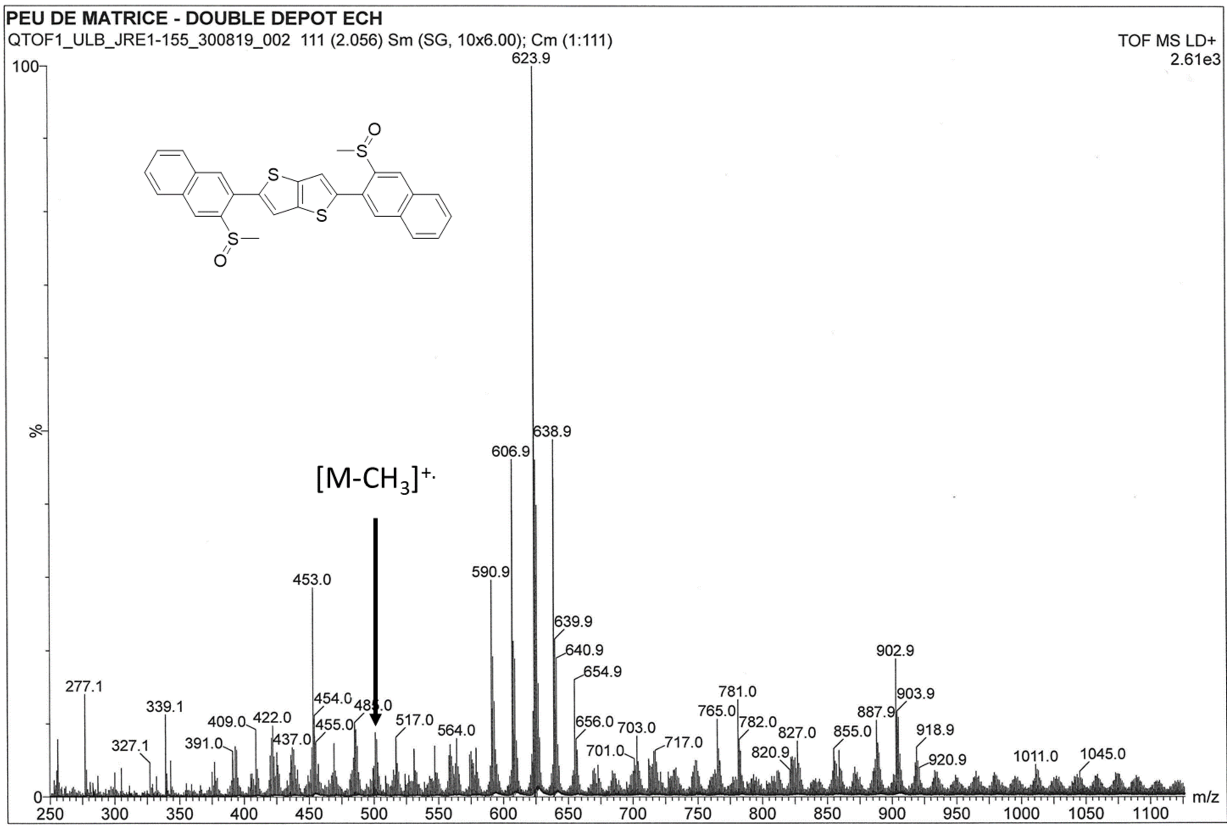
PEU DE MATRICE - DOUBLE DEPOT ECH
QTOF1_ULB_JRE1-155_300819_002 111 (2.056) Sm (SG, 10x6.00); Cm (1:111)
TOF MS LD+
2.61e3
623.9
638.9
606.9
[M-CH3]+·
590.9
453.0
639.9
640.9
654.9
902.9
903.9
277.1
339.1
409.0
422.0
454.0
455.0
437.0
391.0
327.1
517.0
564.0
656.0
703.0
701.0
717.0
765.0
781.0
782.0
827.0
820.9
855.0
887.9
918.9
920.9
1011.0
1045.0
%
m/z

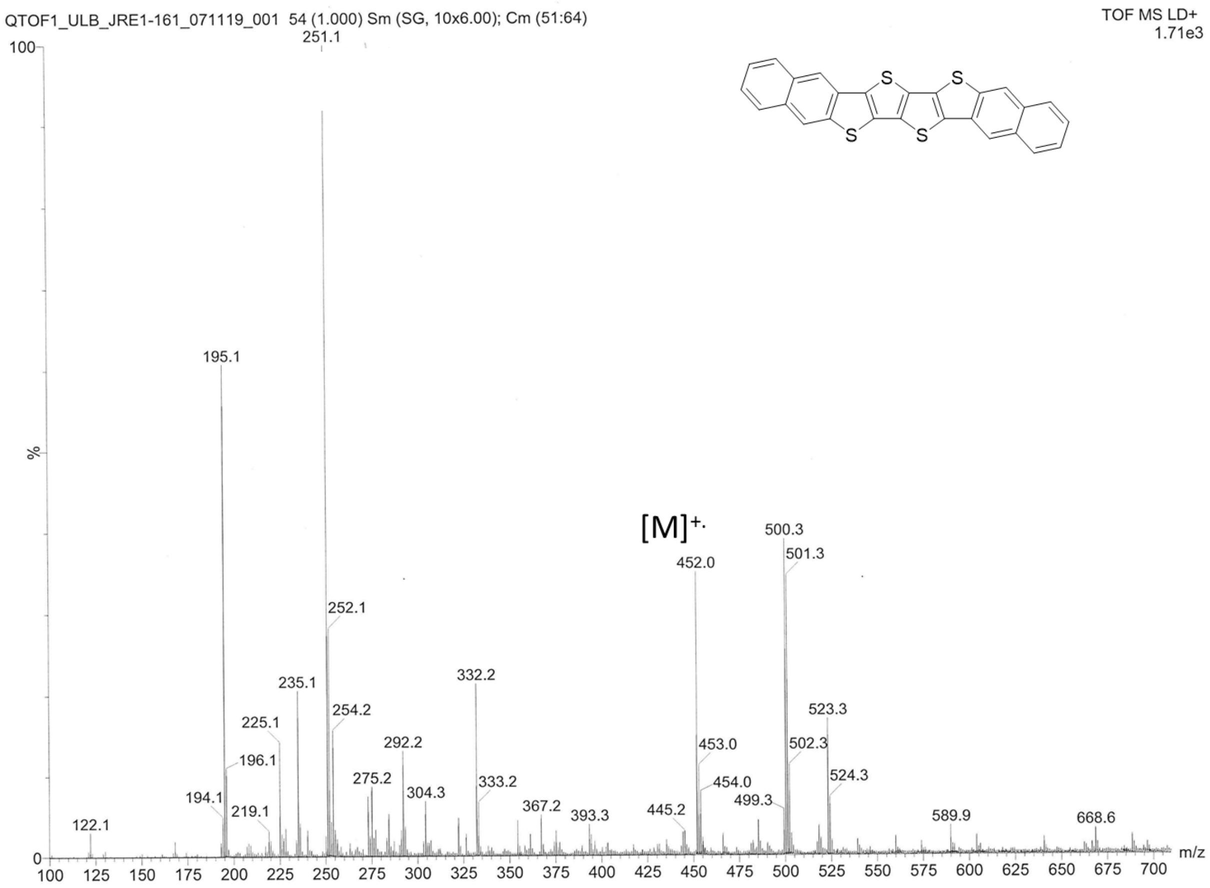
QTOF1_ULB_JRE1-161_071119_001 54 (1.000) Sm (SG, 10x6.00); Cm (51:64)
TOF MS LD+
1.71e3
[M]+·
452.0
500.3
501.3
251.1
195.1
252.1
235.1
332.2
m/z

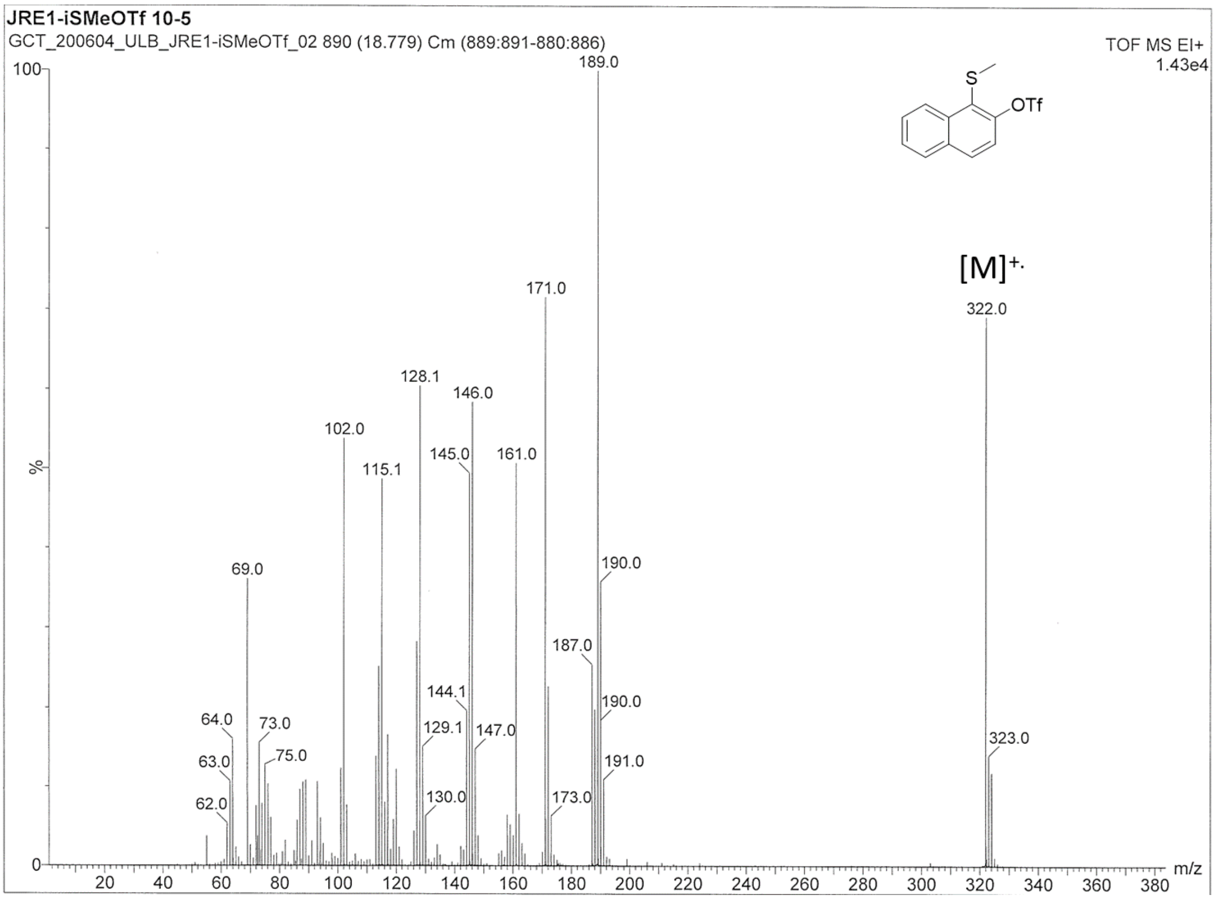
JRE1-iSMeOTf 10-5
GCT_200604_ULB_JRE1-iSMeOTf_02 890 (18.779) Cm (889:891-880:886)
TOF MS EI+
1.43e4
OTf
[M]+·
322.0
189.0
171.0
m/z

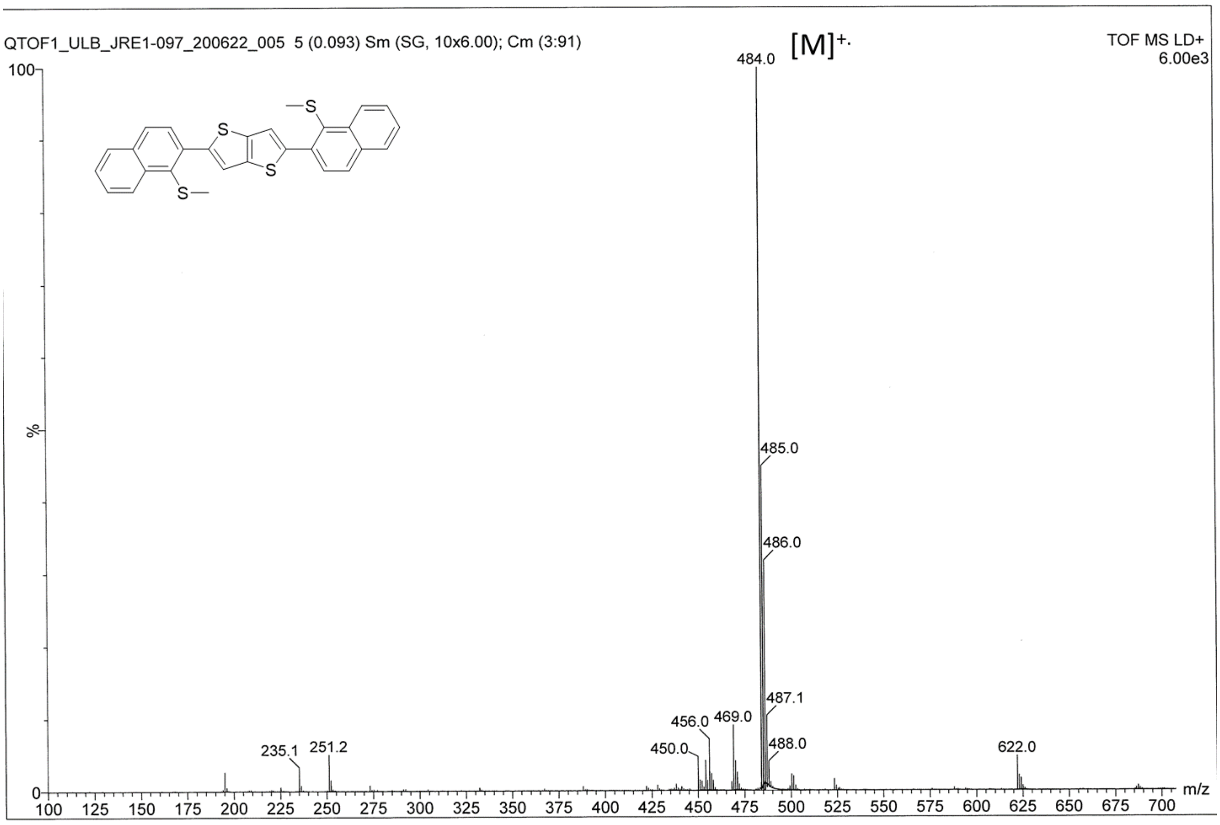
QTOF1_ULB_JRE1-097_200622_005 5 (0.093) Sm (SG, 10x6.00); Cm (3:91)
TOF MS LD+
6.00e3
[M]+·
484.0
485.0
486.0
487.1
488.0
456.0
469.0
450.0
235.1
251.2
622.0
%
m/z

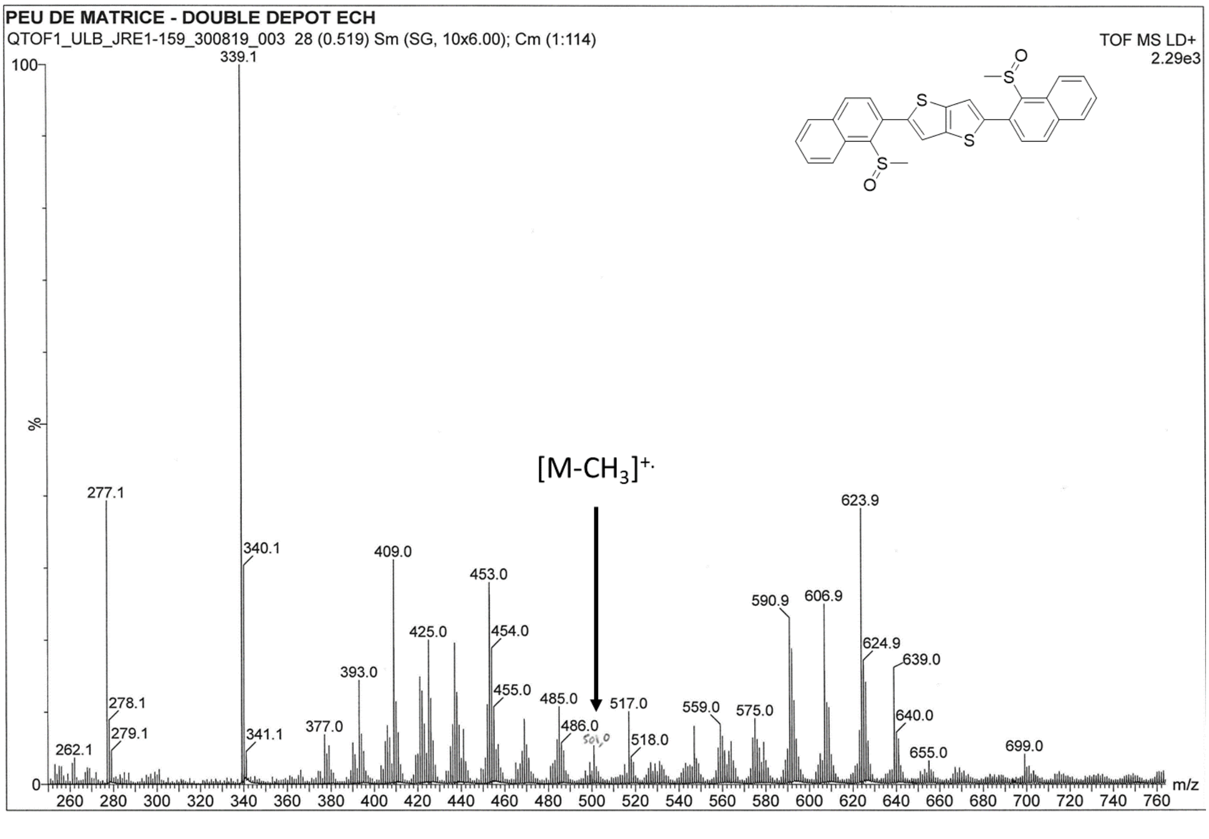
PEU DE MATRICE - DOUBLE DEPOT ECH
QTOF1_ULB_JRE1-159_300819_003 28 (0.519) Sm (SG, 10x6.00); Cm (1:114)
TOF MS LD+
2.29e3
339.1
277.1
340.1
409.0
453.0
[M-CH3]+·
623.9
590.9
606.9
425.0
454.0
624.9
639.0
393.0
455.0
485.0
517.0
559.0
575.0
640.0
278.1
279.1
341.1
377.0
486.0
518.0
655.0
699.0
262.1
%
m/z

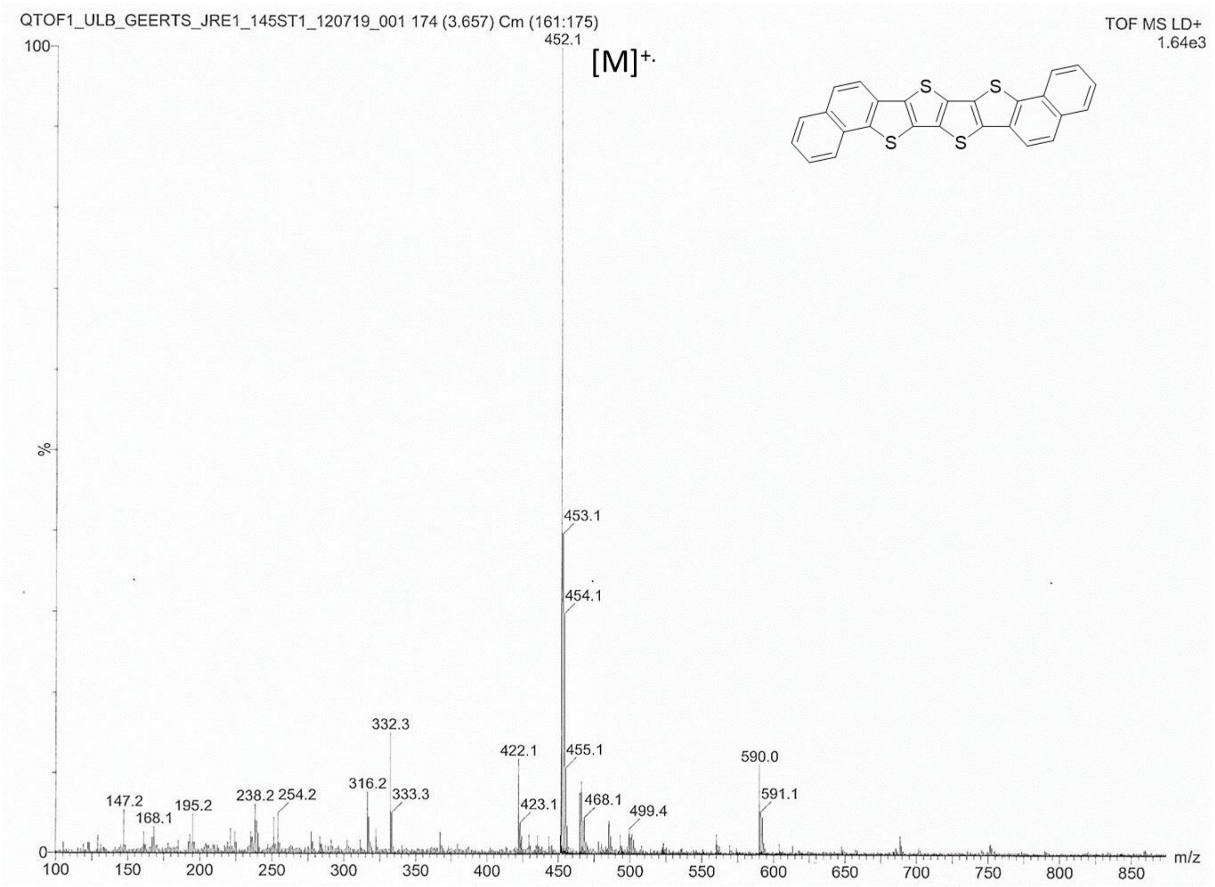
QTOF1_ULB_GEERTS_JRE1_145ST1_120719_001 174 (3.657) Cm (161:175)
TOF MS LD+
1.64e3
452.1
[M]+·
453.1
454.1
332.3
422.1
455.1
590.0
316.2
333.3
147.2
168.1
195.2
238.2
254.2
423.1
468.1
499.4
591.1
%
m/z

1.4. Partial assignation of ^{1}H NMR signals of DN4T **and** isoDN4T

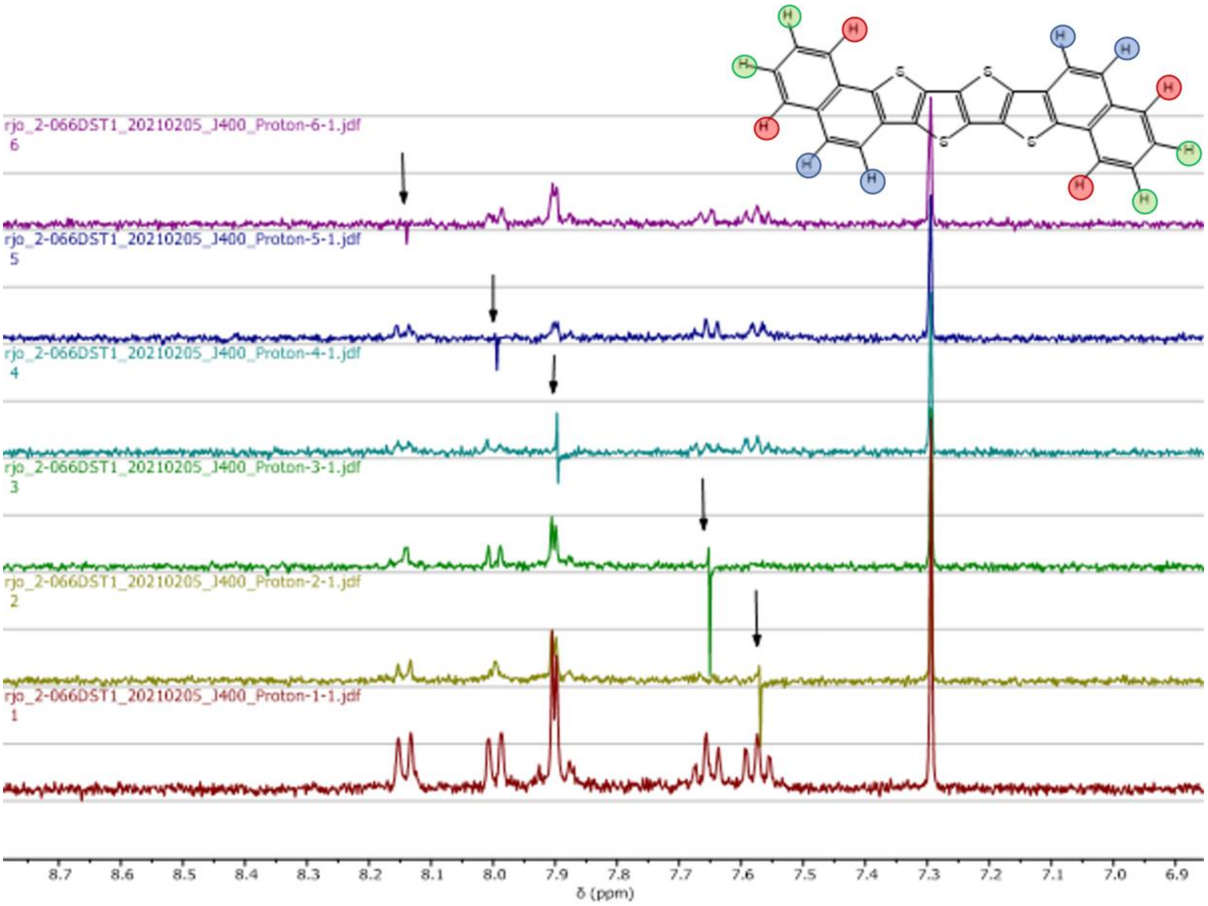

Figure S1. Assignation of ^{1}H NMR signals of isoDN4T by comparison of its ^{1}H NMR spectrum (red line, bottom) with a series of ^{1}H homodecoupled NMR experiments (yellow to purple lines). Arrows show the saturation frequencies of each NMR experiment, that cancel all the couplings with the corresponding saturated signal on the spectrum.

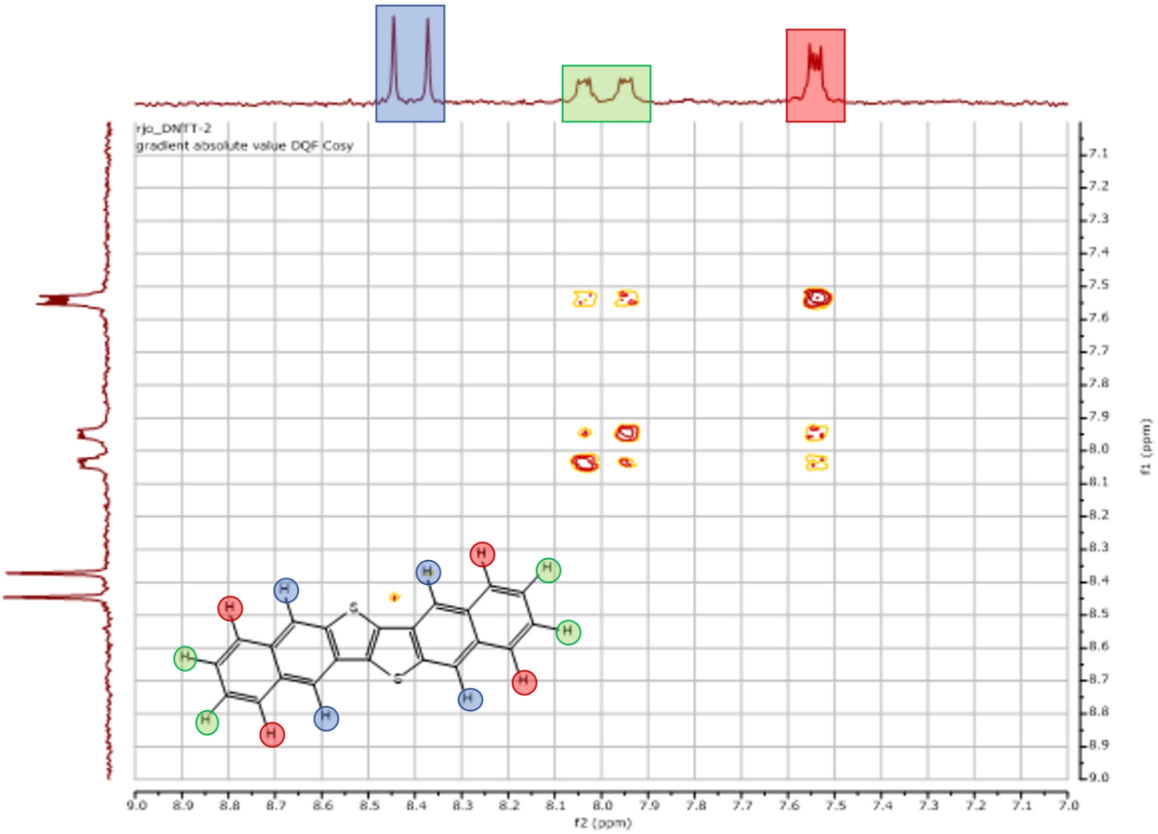

Figure S2. Assignation of ^{1}H NMR signals of **DNTT** using a COSY ^{1}H experiment.

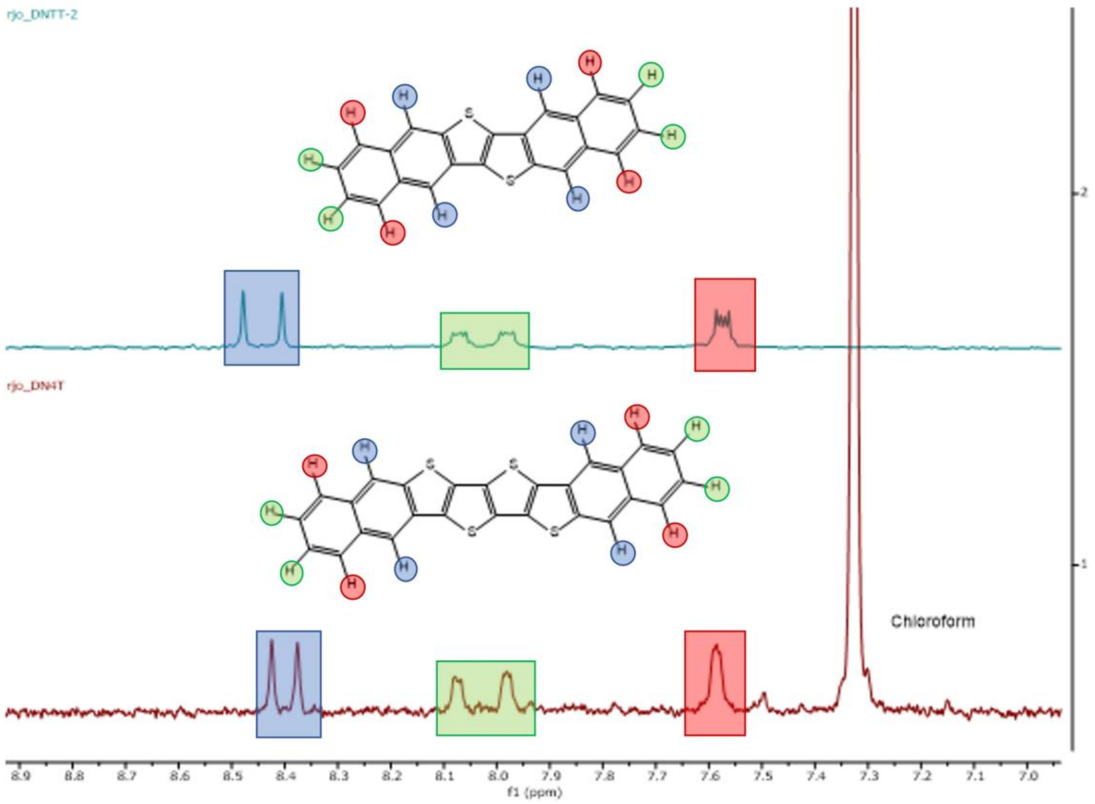

Figure S3. Comparison of the ^{1}H NMR spectra of **DNTT** (top) and DN4T (bottom) in deuterated 1,1,2,2-tetrachloroethane at 120°C. The strong similarity between both spectra enabled us to assign the signals of DN4T from the assigned signals of **DNTT** using the COSY NMR of **Figure S2**.

2. Experimental UV-vis spectra

The optical absorption spectra, shown in **Figure S4**, were recorded with a Perkin Elmer's Lambda 950UV/Vis/NIR spectrophotometer. A background correction was performed, prior to measurement, for 100% transmittance or 0 absorbance over the wavelength range. The spectra of DN4T and isoDN4T were both taken both in solution (1,2,4 trichlorobenzene) and in thin-film (quartz glass substrate). Thin films of 10 nm nominal thickness were grown by thermal evaporation in ultra-high vacuum (UHV 10^{-8} mbar). Maximum absorption wavelengths of solutions of DN4T and isoDN4T in TCB are 421 and 394 nm respectively, and 448 and 348 nm for thin films, respectively. In the case of isoDN4T in solution, strong absorption from aggregates is observed at a concentration of ~1 mg/mL concentration, as can be seen from the lower energy shoulder of the peak at 3.15 eV in **Figure S4** (c). Upon dilution we observe that the aggregate absorption is reducing until its essentially gone around ~0.02 mg/mL.

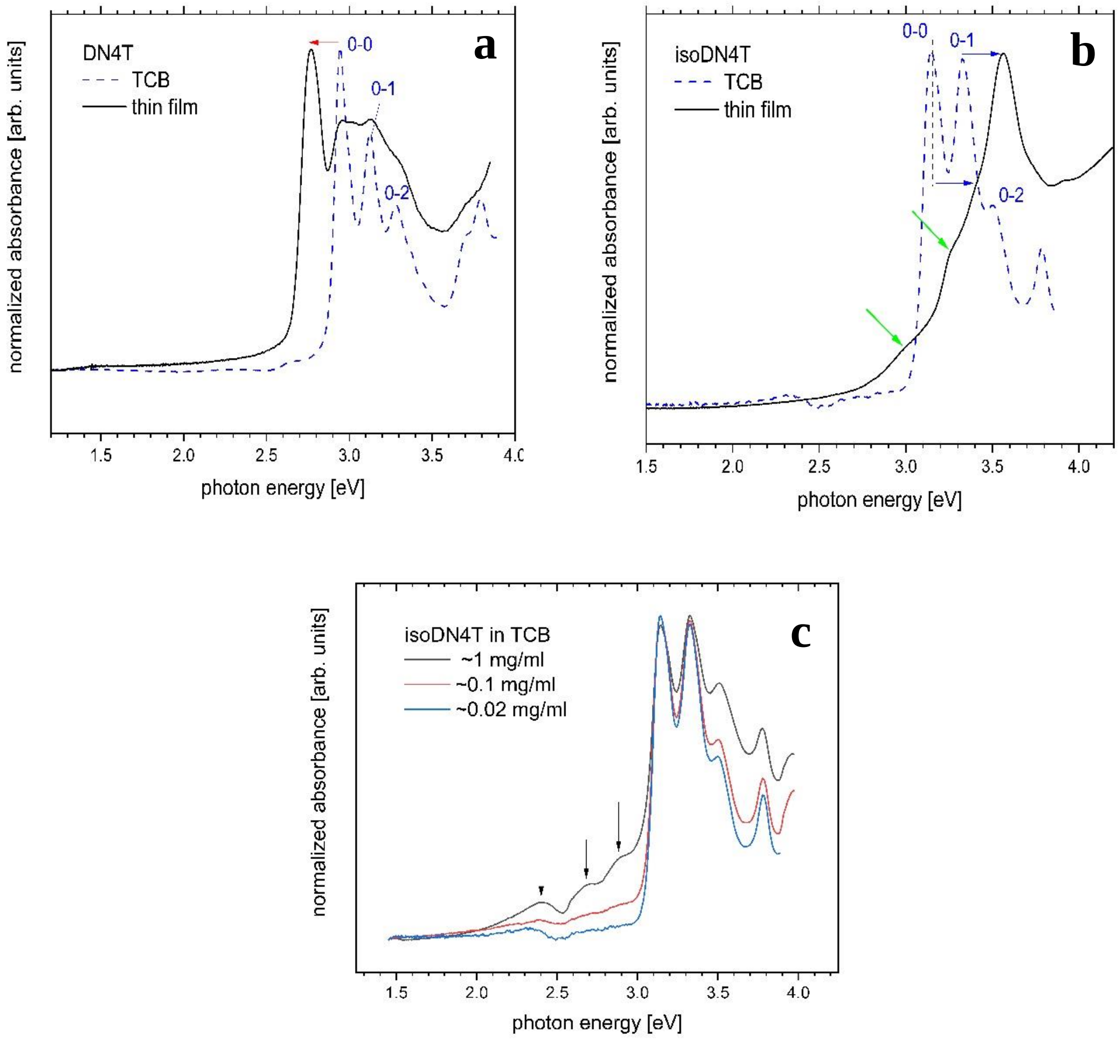

Figure S4. UV-vis absorption spectra from thin-films and solutions of: a) DN4T and b) isoDN4T. The red and blue arrows points at the direction of the shift from solution to thin-film, while the green arrows in the case of isoDN4T show partly-allowed transitions due to structural disorder. c) Dilution series of iso-DN4T in TCB.

3. Crystal structures and Hirshfeld Surface Analysis

3.1. Single-crystal X-ray diffraction (SCXRD)

The diffraction data of DN4T and isoDN4T were collected on a Rigaku Oxford Diffraction XtaLAB Synergy diffractometer equipped with a HyPix-6000HE area detector using Cu K_α (λ = 1.54184 Å) from PhotonJet micro-focus X-ray Source from PhotonJet. The diffraction images were processed and scaled using the *CrysAlis^{Pro}* software suite (Rigaku Oxford Diffraction. *CrysAlis^{Pro}* Software system, version 1.171.40.25a, Rigaku Corporation: Oxford, UK, (2018)).The structure was solved using the charge-flipping algorithm, as implemented in the program *SUPERFLIP*[2] and refined by full-matrix least-squares techniques against F^2 using the SHELXL program[3] All non-H atoms in the structure were refined with anisotropic thermal parameters, and hydrogen atoms were placed in calculated positions. The structures were examined using the *Addsym* subroutine of PLATON[4] to ensure that no additional symmetry could be applied to the models. Crystallographic data collection and refinement parameters are collated in **Table S1** and full crystallographic details in CIF format have been deposited in the CCDC, reference numbers 2103738 and 2103739 for DN4T and isoDN4T, respectively. Measurements on L-DBTTA were made with a Rigaku Synergy-i instrument using *CrysAlis^{Pro}* software for data collection and reduction. The solution was found by direct methods using SHELXT and the structure was refined to convergence against F^2 using all unique reflections and the program SHELXL-2018 as implemented within WINGX.[3,5] All H atom positions were observed by difference synthesis but the H atoms were placed in idealized positions and refined in riding modes. C-H bond length was 0.95 Å and U_{iso}(H) = 1.2U_{eq}(C) of parent atom. Selected crystallographic details and refinement parameters are given in **Table S1** and full crystallographic details in CIF format have been deposited with the CCDC, reference number 2092479.

Despite the good reliability of the crystal structure of DN4T solved from SCXRD results, the possibility for a centrosymmetric molecule to pack in a polar space group is actually still a debate,[6] and as a matter of fact one suggestion resulting from the PLATON analysis of this crystal structure was to add an inversion center to it, yielding in the space group $P\ 2_1/m$ instead of the current $P\ 2_1$. However, the DN4T molecule is not planar according to the XRD results (notably showing two bend angles of 5.6° and 8.4° between the tetrathienyl core and the two naphthalene rings), and in addition the molecule is not perpendicular to the b axis of the crystal lattice. Moreover, switching from $P\ 2_1$ to $P\ 2_1/m$ led to only 80% overlap of atoms with respect to the raw data according to PLATON, and finally the initial crystal structure was already ordered and occupied reasonable displacement parameters. We thus kept the $P\ 2_1$ the space group in DN4T's crystal structure for being the one that effectively fits the best the collected data from SCRXD on DN4T crystals.

Nevertheless, the crystal structure of DN4T also initially included a commonly used DFIX restraint on the length of the C-C bonds of the naphthyl rings, that could be inappropriate for this particular crystal structure. We thus tried to remove the DFIX restraint on the C-C bonds, which slightly improved the R-factor of DN4T's crystal structure (see **Table S1**, second column). However, as all the calculations coming from DN4T's crystal structure in this work (i.e., the determination of the transfer integrals, the molecular dynamics, and the kinetic Monte Carlo simulations) have been based on the initial crystal structure (see section 5 of this document), we performed another transfer integrals calculation from the DFIX restraint-free one as a comparison. As a matter of fact, the values calculated using the DFIX restaint *vs* DFIX restraint-free crystal structures are 93 meV *vs* 99 meV, 36 meV *vs* 33 meV and 59 meV *vs* 61 meV, giving evidence of negligible differences in the electronic coupling values between the two crystal structures. We thus considered all the results coming from the initial crystal structure of DN4T presented in this work to be satisfyingly accurate, and most importantly not affecting the comparison of its properties with the other aromatic cores also discussed in this article. Note that consequently, for the sake of consistency, the corresponding CIF file related

to DN4T available with this article is the one from which all the calculations have been done, i.e., still including the DFIX restraint on the length of the C-C bonds of the naphthyl rings.

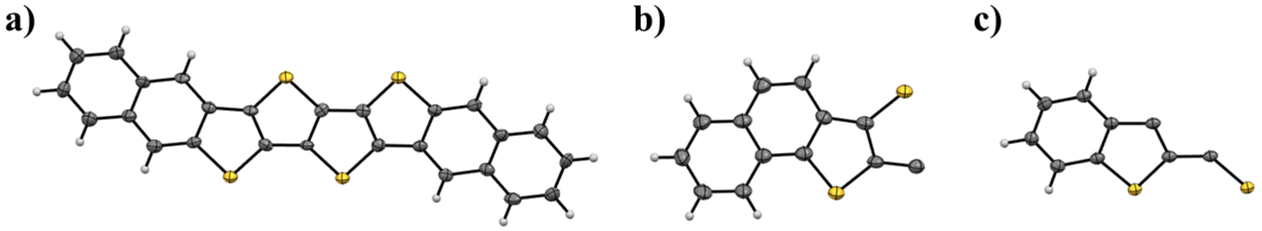

Figure S5. Asymmetry units for molecules (a) DN4T, (b) isoDN4T and (c) L-DBTTA. Displacement ellipsoids are drawn at the 50% probability level.

Compound	DN4T	DN4T**(*)**	isoDN4T	L-DBTTA
Formula	$C_{26}H_{12}S_4$	$C_{26}H_{12}S_4$	$C_{26}H_{12}S_4$	$C_{18}H_8S_4$
Temperature [K]	172(2)	172(2)	100(2)	100(2)
Crystal system	Monoclinic	Monoclinic	Monoclinic	Monoclinic
Space group	$P\ 2_1$	$P\ 2_1$	$P\ 2_1/c$	$P\ 2_1/c$
a [Å]	6.15993(17)	6.15993(17)	19.1163(18)	15.8012(5)
b [Å]	7.5925(2)	7.5925(2)	7.6335(8)	3.89720(10)
c [Å]	20.0487(6)	20.0487(6)	6.5402(5)	11.4207(4)
β [deg]	91.695(3)	91.695(3)	92.970(8)	103.844(3)
Volume [$Å^3$]	937.25(5)	937.25(5)	953.09(16)	682.86(4)
Z	2	2	2	2
Z'	1	1	0.5	
Density (calculated) [g cm^{-3}]	1.604	1.604	1.577	1.714
Goodness-of-fit on F^2	1.081	1.012	1.119	1.028
Final R indices [I>2sigma(I)]	R_1 = 0.0482, wR_2 = 0.1255	R_1 = 0.0454, wR_2 = 0.1172	R_1 = 0.0907, wR_2 = 0.2489	R_1 = 0.0404, wR_2 = 0.1134

Table S1. Crystal data collection and structure refinement for DN4T, isoDN4T and L-DBTTA. The first column presents the data corresponding to the crystal structure of DN4T solved with a DFIX restraint on the length of the C-C bonds of the naphthyl rings and is the one that has been used in all the calculations regarding this molecule in this work. The second column (*) present the data corresponding to the crystal structure of DN4T solved without this DFIX restraint.

3.2. Hirshfeld Surface Analysis

The Hirshfeld surfaces[7,8] were generated through *CrystalExplorer17* software.[9] In *CrystalExplorer*, X-H bond lengths were normalized to be the same as those obtained from neutron diffraction experiments (e.g. C-H=1.083 Å).[10] The normalized contact distance (d_{norm}) based on d_e (the distance from the point on the surface to the nearest nucleus external to the surface) and d_i (the distance from

the point on the surface to the nearest nucleus internal to the surface) and van der Waals radii of the atom is calculated by :

$$d_{norm} = \frac{(d_i - r_i^{vdw})}{r_i^{vdw}} + \frac{(d_e - r_e^{vdw})}{r_e^{vdw}} \quad (1)$$

where r_i^{vdw} and r_e^{vdw} are the van der Waals radii of the atoms. The parameter d_{norm} is negative or positive, which is illustrated by a surface with a red–white–blue colored graph. On the surface, red spots represent the lengths shorter than the van der Waals distance, whilst white and blue regions show lengths around and longer than the van der Waals distance, respectively.

2D fingerprint plot derived from a Hirshfeld surface[7,11] reveals visually the frequency of each combination of d_e and d_i over the molecular surface. The color on the plot with a range from blue (relatively few points) through green (moderate fraction) to red (highest fraction) reflects the contribution from different interatomic contacts.

Curvedness surface,[8] a measure of "how much" shape is useful to measure curvature, offers further chemical insight into molecular packing. Low values of curvedness designate essentially a flat region of the surface and may be a sign of π-π stacking in the crystal. High curvedness is highlighted as dark-blue edges and tends to divide the surface into patches. Curvedness, C, is given by :

$$C = \frac{2}{\pi} ln\sqrt{k_1{}^2 + k_2{}^2/2} \quad (2)$$

Where, k_1 and k_2 are principal curvatures.

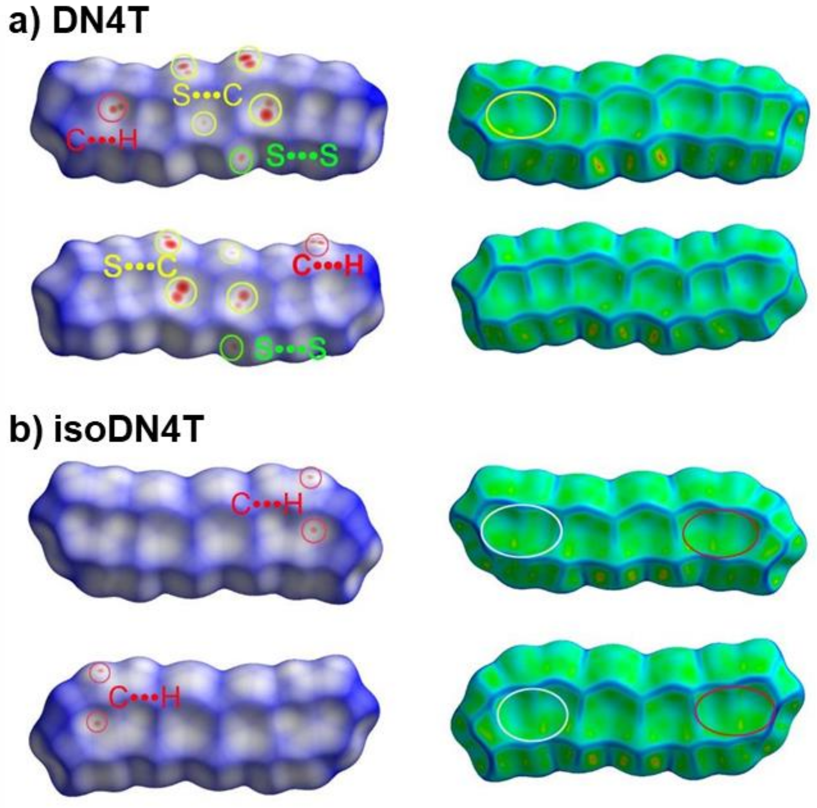

Figure S6. Front and back views of Hirshfeld surfaces (mapped over a d_{norm} range of -0.1 to 1.15 Å, left) and curvedness surfaces (right) of DN4T (a) and isoDN4T (b). Curvedness surfaces clearly show that there are no large flat surface patches on both sides of DN4T and isoDN4T, illustrating no planar stacking between molecules. The front and back planes of the two naphthyl groups display identical local flat regions in isoDN4T (highlighted with white and red ellipses), rather than only one side of

one naphthyl plane in DN4T (yellow ellipse), exhibiting slightly bent-shape geometry with an angle of 5.7° in isoDN4T and 5.6°/8.4° in DN4T mean plane of naphthyl and central fused thiophene units.

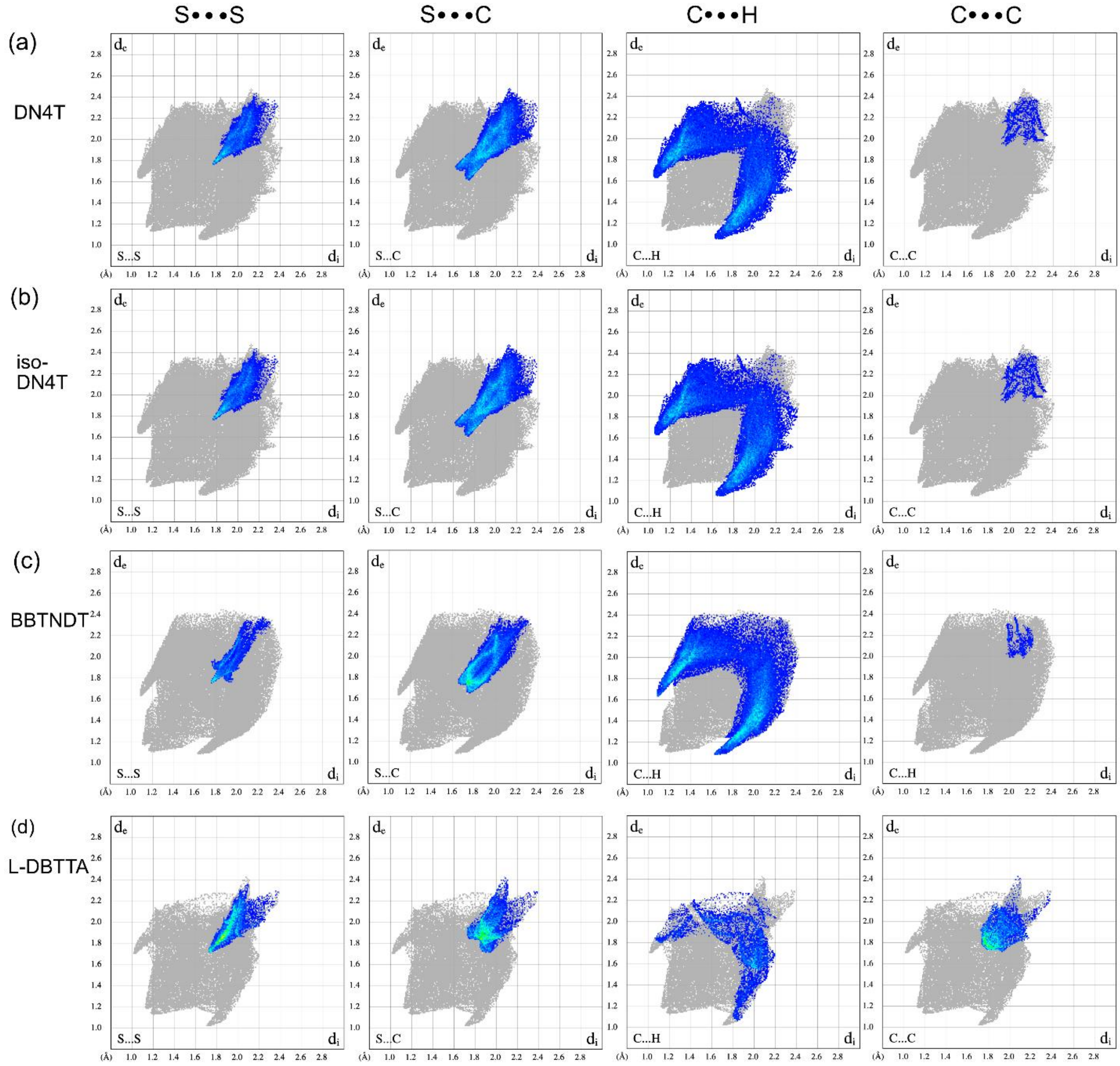

Figure S7. 2D fingerprint plots of DN4T (a), isoDN4T (b), **BBTNDT** (c) and L-DBTTA (d) limited to C/S···C/S and C···H interactions.

3.3. Variable Temperature Powder X-Ray Diffraction

Powder X-ray diffraction patterns in different temperature points were obtained using a Panalytical Empyrean X-ray diffractometer [Cu Kα (λ = 1.54184 Å) radiation] with a TTK 600 variable temperature stage attached. For both DN4T and isoDN4T, the samples were ground using a mortar and pestle and mounted on the sample holder over a zero-background film. The program on the diffractometer was set to obtain the patterns in vacuum using a gonio scan with 2θ range from 5 ° to 40 °, scan speed of 0.11 °/s and steps of 0.13 °, in different temperatures, varying from 93 K to 303 K in steps of 30 K.

The unit cell parameters of DN4T were obtained using XCell program within Reflex Powder Indexing tool, which is part of Materials Studio software[12] and space group $P\ 2_1$. Pawley refinement[13,14] with ultra-fine quality with Pseudo-Voigt function was used for the refinement of the FWHM, profile and line-shift parameters, as well as unit cell parameters. For isoDN4T, patterns at variable temperature were indexed using EXPO2014 software.[15] Unit cell parameters and crystal structure obtained from single-crystal x-ray diffraction were used as input. The calculated powder patterns were refined against the experimental pattern using Rietveld refinement,[16,17] with 2θ range between 8° to 30°, automatic refinement of the profile, non-structural parameters with the Le Bail fitting[18] and line shifts.

Calculated diffractograms were obtained from the crystal structures presented in this work using the Powder Pattern tool on Mercury software.[19,20] X-Ray diffraction patterns are illustrated in **Figure S8**, and details of the unit cell parameters and fitting of the experimental powder patterns can be observed in **Table S2**. As it can be observed in **Figure S9**, there are no great variations in the unit cell parameters from low temperature (93 K) to room temperature (303 K) with data for each axis or angle varying less than 1.5 % in comparison with the value obtained by single-crystal XRD.

			DN4T				
T [K]	a (σ_a) [Å]	b (σ_b) [Å]	c (σ_c) [Å]	β (σ_β) [deg]	V (σ_V) [Å^3]	R_{wp} [%]	R_p [%]
SC-XRD (173K)	6.1599 (17)	7.5925 (2)	20.0487 (6)	91.695 (3)	937.25 (5)		
303.0	19.99 (4)	7.643 (7)	6.155 (5)	91.867 (4)	940.0 (5)	13.57	9.86
273.0	19.96 (4)	7.620 (16)	6.143 (13)	91.864 (7)	933.8 (7)	11.88	9.09
243.0	19.99 (4)	7.630 (16)	6.154 (13)	91.867 (6)	938.4 (7)	11.48	8.60
213.0	20.01 (4)	7.632 (16)	6.159 (12)	91.852 (6)	940.2 (7)	11.99	9.15
183.0	19.98 (4)	7.614 (15)	6.150 (12)	91.837 (6)	935.2 (7)	12.20	9.31
153.0	20.02 (4)	7.622 (16)	6.164 (13)	91.780 (7)	940.2 (7)	12.61	9.49
123.0	20.05 (4)	7.628 (16)	6.173 (12)	91.767 (6)	943.8 (7)	11.71	8.65
93.0	19.91 (4)	7.567 (14)	6.127 (12)	91.762 (5)	922.7 (6)	11.36	8.51
			isoDN4T				
T [K]	a (σ_a) [Å]	b (σ_b) [Å]	c (σ_c) [Å]	β (σ_β) [deg]	V (σ_V) [Å^3]	R_{wp} [%]	R_p [%]
SC-XRD (100K)	19.1163 (18)	7.6335 (8)	6.5402 (5)	92.969 (8)	953.09 (15)		
303.0	19.145 (4)	7.702 (2)	6.557 (1)	92.731 (18)	965.7 (4)	13.68	9.46
273.0	19.165 (4)	7.709 (3)	6.558 (2)	92.784 (17)	967.7 (5)	14.55	10.38
243.0	19.300 (6)	7.747 (3)	6.599 (2)	92.923 (18)	985.3 (6)	16.18	12.45
213.0	19.290 (6)	7.742 (3)	6.596 (2)	92.950 (20)	983.7 (6)	16.24	12.36
183.0	19.147 (5)	7.694 (3)	6.552 (2)	92.820 (20)	964.1 (6)	17.29	13.09
153.0	19.167 (5)	7.689 (3)	6.556 (2)	92.848 (18)	965.1 (5)	15.57	11.56
123.0	19.177 (5)	7.692 (2)	6.553 (2)	93.000 (20)	965.2 (5)	15.92	11.96
93.0	19.152 (4)	7.673 (3)	6.544 (2)	92.955 (20)	960.5 (5)	15.56	11.83

Table S2. Lattice parameters and weighted and unweighted profile R-factors (R_{wp} and R_p, respectively) for DN4T and IsoDN4T for the indexed variable temperature powder diffractograms.

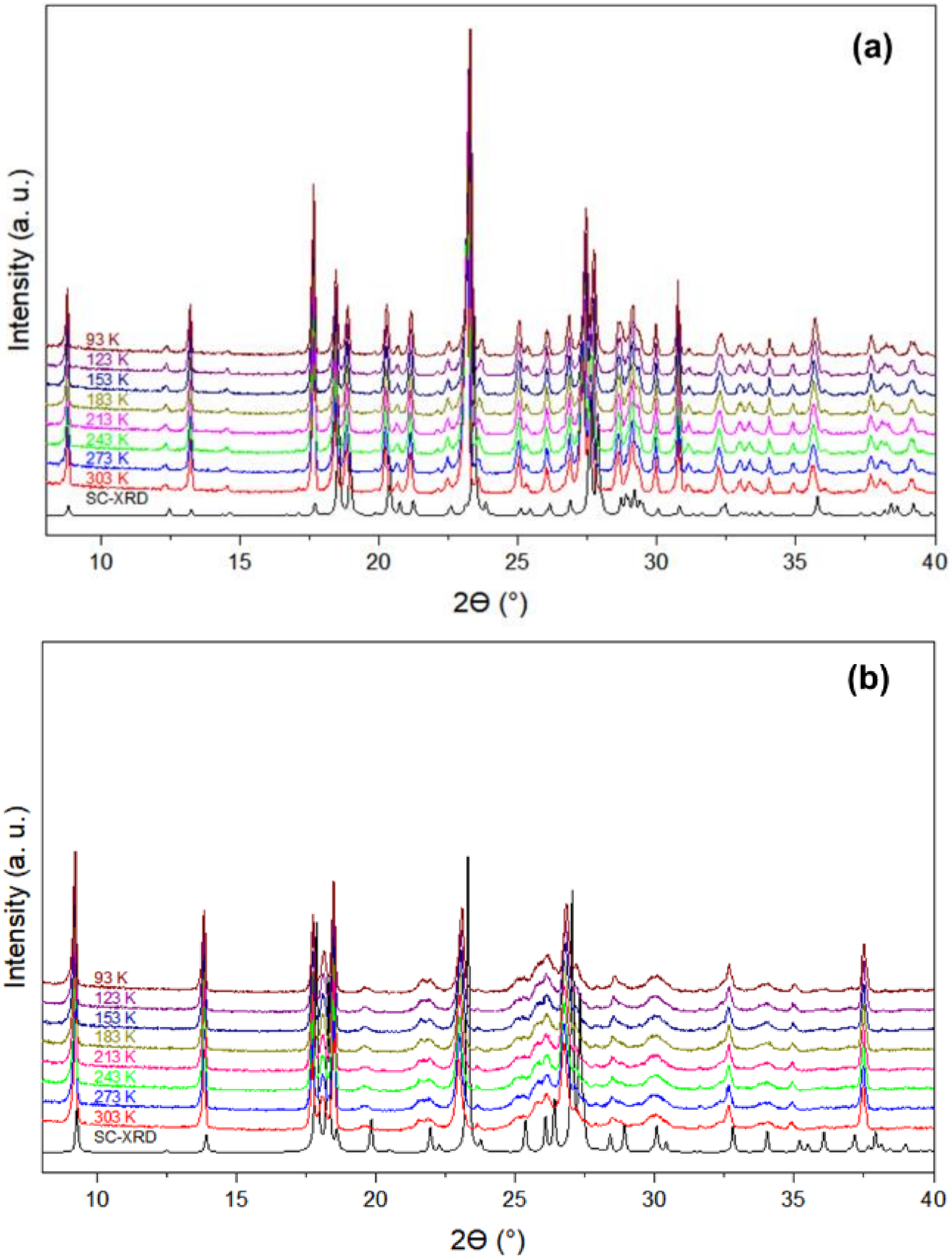

Figure S8. X-Ray diffraction patterns increasing in temperature from the top (brown curve, 93 K) to the bottom (red curve, 303 K) and calculated pattern from crystal structures (black curve) for (a) DN4T and (b) IsoDN4T.

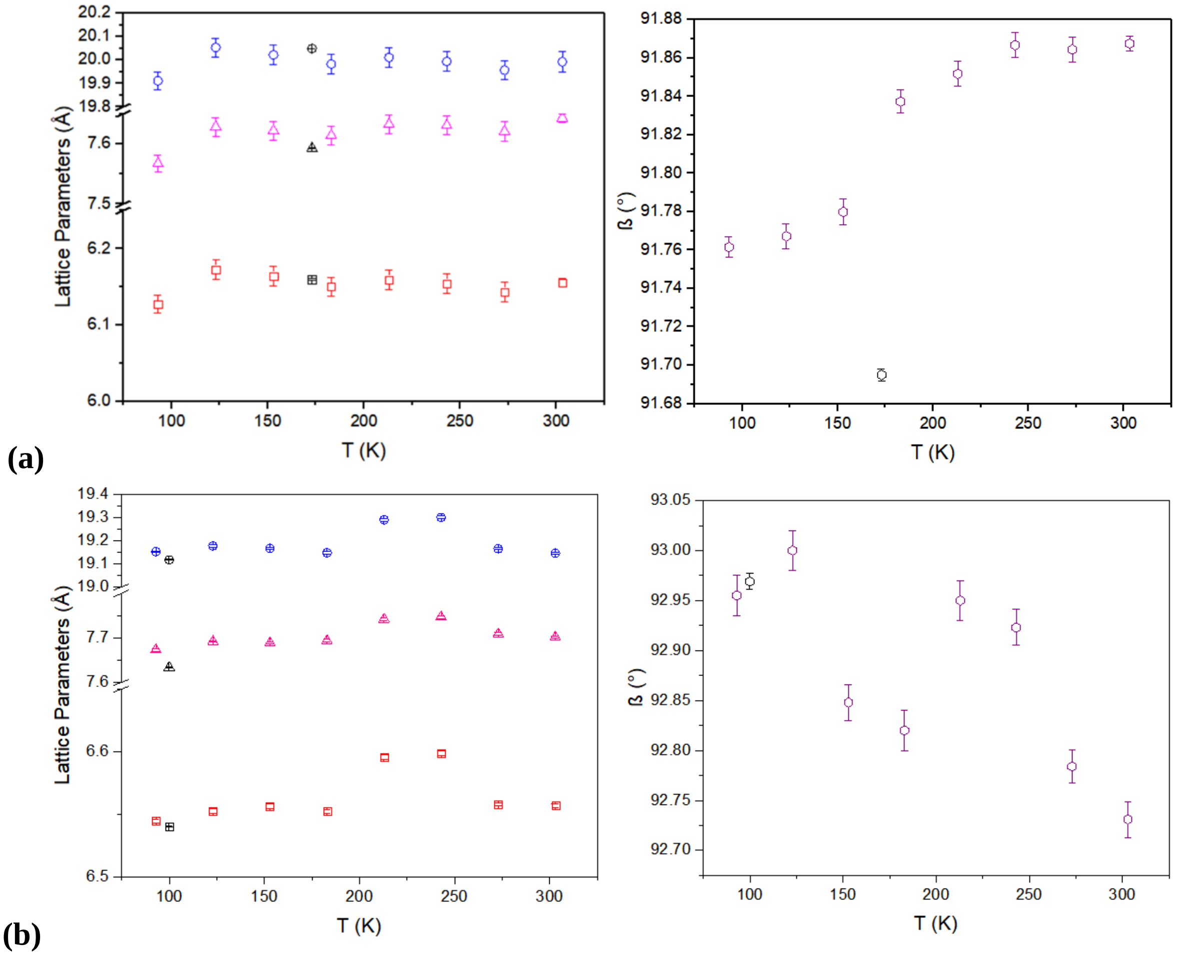

Figure S9. On the left, variation of the unit cell dimensions according to the temperature. Red squares represent *a* axis; pink triangles represents *b* axis and blue circles represents *c* axis; and on the left, angle variation according to the temperature (purple hexagon) for (a) DN4T and (b) isoDN4T. For comparison, the lattice parameters obtained from single-crystal x-ray diffraction are observed in black.

4. Physicochemical properties

4.1. Thermogravimetric analysis (TGA)

Thermogravimetric analysis (TGA) measurements were conducted on a Pyris 6 TGA instrument with Pyris software. About 7 mg of DN4T and isoDN4T were placed in an open ceramic crucible and were scanned at a rate of 5 °C/min under a nitrogen flow at 20 mL/min. TGA curves reveal that sublimation of both compounds starts around 400°C. First derivatives clearly show that no thermal event takes place before 400 °C for both compounds, suggesting that DN4T and isoDN4T possess high thermal stability.

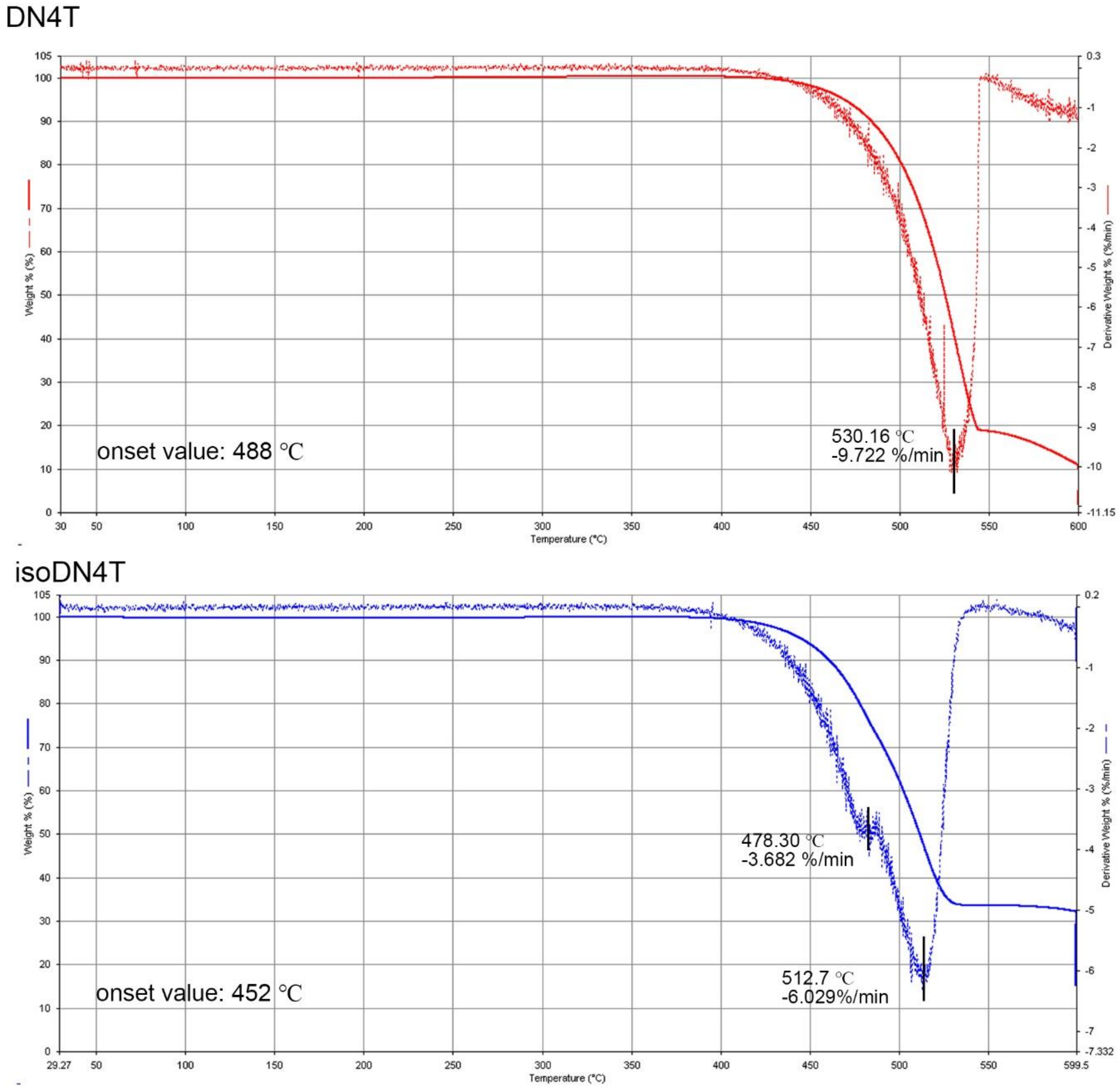

Figure S10. TGA curves of DN4T (red solid line) and isoDN4T (blue solid line); first derivatives of DN4T (red dotted line) and isoDN4T (blue dotted line). The data were recorded with a heating rate of 5°C/min.

4.2. Differential Scanning Calorimetry (DSC)

DSC analysis was performed on a Netzsch Polyma 214 instrument with Proteus software. Approximately 5 mg of DN4T and isoDN4T were placed in a covered aluminum crucible with pierced lids. The heating and cooling rates of 10°C/min were adopted. The dry nitrogen acted as both purge and protect gas at 20 mL/min. No phase transitions are observed up to 300°C.

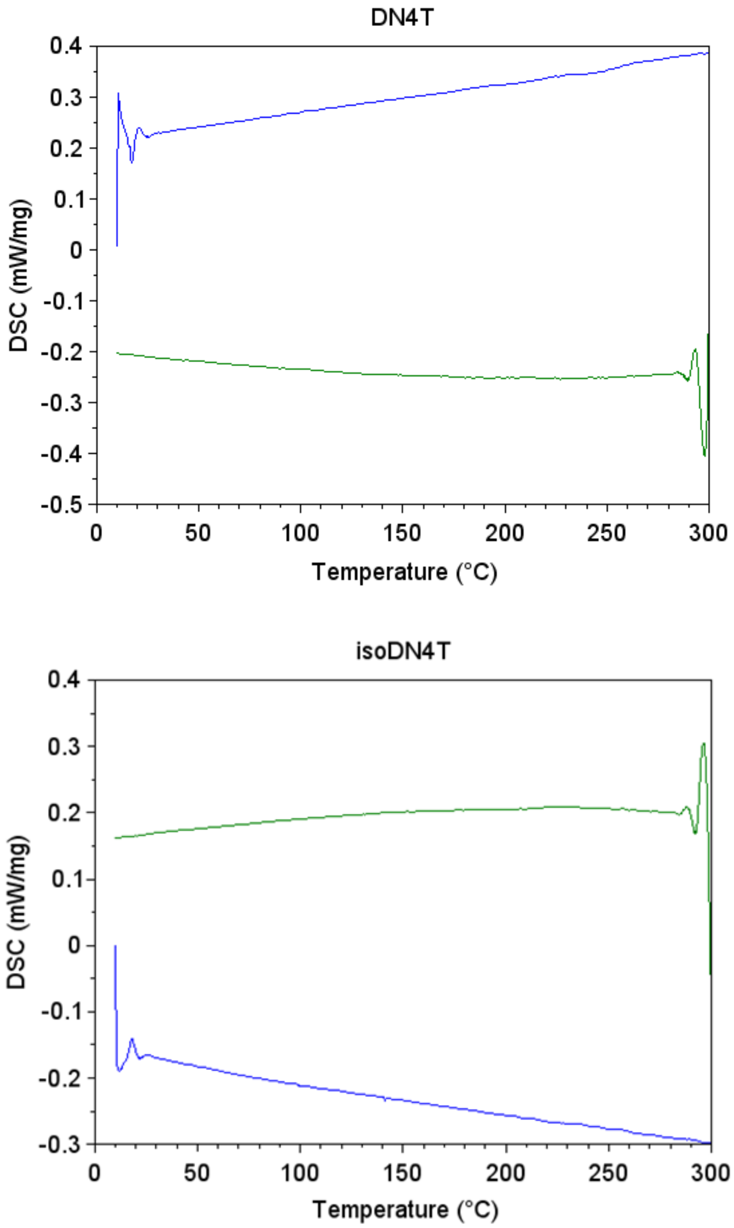

Figure S11. DSC curves of DN4T (top) and isoDN4T (bottom) recorded at 10°C/min. No phase transition could be observed between 20 and 300°C.

5. Quantum calculations

5.1. Methods

The Gaussian 16-A03 package was used to optimize the geometry, to compute the electronic structure of the neutral isomers and to estimate their reorganization energies at the Density Functional Theory (DFT) level using the B3LYP functional and a 6-31G(d,p) basis set.[21] The reported experimental crystal structures at 100K/173K for DN4T/Iso-DN4T were used as input to calculate transfer integrals between close neighbors in the frozen crystal structure. The ADF package was used to estimate these parameters at the DFT B3LYP/DZ level of theory within a fragment orbital approach.[22,23] In practice, we created a 3×3×3 supercell for each molecule and computed electronic couplings among all pairs of close neighbors involving molecules in the unit cell at the center of the system. A close neighbor to a given molecule A is defined as any molecule B for which at least one atom is within a 5 Å range of any atom of molecule A.

The reported experimental X-ray structures were also used as inputs for the Molecular Dynamics simulations on the crystals, aimed at analyzing the fluctuations in the transfer integrals induced by thermal disorder. To do so, we exploited the oplsaa force field, as assigned by the LigParGen server[24] and implemented in the LAMMPS package;[25] this is motivated by the fact that this force field was recently applied with success to organic semiconductors made of fused aromatic rings to depict lattice vibrations and their role in heat transport.[26] The force field was modified by quadrupling the coefficients of the improper terms to avoid excessive bending of the molecules. 4×4×4 supercells were generated, optimized at 0K and heated up first at 100K, then at 298K in the NVT ensemble for a 1ns recording run. 500 snapshots of the dimers of interest were selected in the final 3ns of the NVT run at room temperature.

Mobility anisotropy plots were computed in the scope of a hopping regime, with a Kinetic Monte Carlo algorithm based on the first reaction method.[27] In this method, a single charge is set at a localized site (i.e., molecular unit) at t=0 and hopping rates are computed as a function of the transfer integrals and reorganization energies previous calculated at the quantum-chemical level. Waiting times are generated randomly for each hopping process, and the one with the shortest waiting time is selected. The simulation clock is then advanced by the chosen waiting time, the distance d travelled by the charge is incremented by the distance between the two centers of mass of the molecules involved in the charge transfer. This cycle repeats until simulation clock reaches a time limit set to 10^{-9} s. The mobility is computed according to the equation:

$$\mu = \frac{d}{t.E} \quad (3)$$

Where E is the amplitude of the electric field (set to 1000 V/cm). Mobilities were computed for different directions of the electric with regard to the unit cell vector a, from θ=0° to θ=350°.

5.2. Calculation of the reorganization energy

The contribution of each intramolecular vibrational mode to the nuclear relaxation energy occurring when going from the neutral to charged geometry has been estimated within a displaced harmonic oscillator model, while neglecting Duchinsky rotation effects[28] :

$$E_{rel} = \sum_i \frac{g^2(i)}{\hbar\omega_i} = \sum_i S_i \hbar\omega_i \quad (4)$$

with the index i running over all intramolecular vibrational modes of energy $\hbar\omega_i$. g(i) is the local electron-phonon coupling constant associated to the normal coordinate Q_i that has been computed numerically and S_i the corresponding Huang-Rhys factor.

$$g^2(i) = \frac{V^2(i)}{2M_i\hbar\omega^3} \; and \; V^2(i) = (\frac{\partial^2 E}{\partial Q_i^2})_{Q=0} \quad (5)$$

with M_i the effective mass of mode i. **Figure S12** depicts the corresponding distribution for DN4T and isoDN4T.

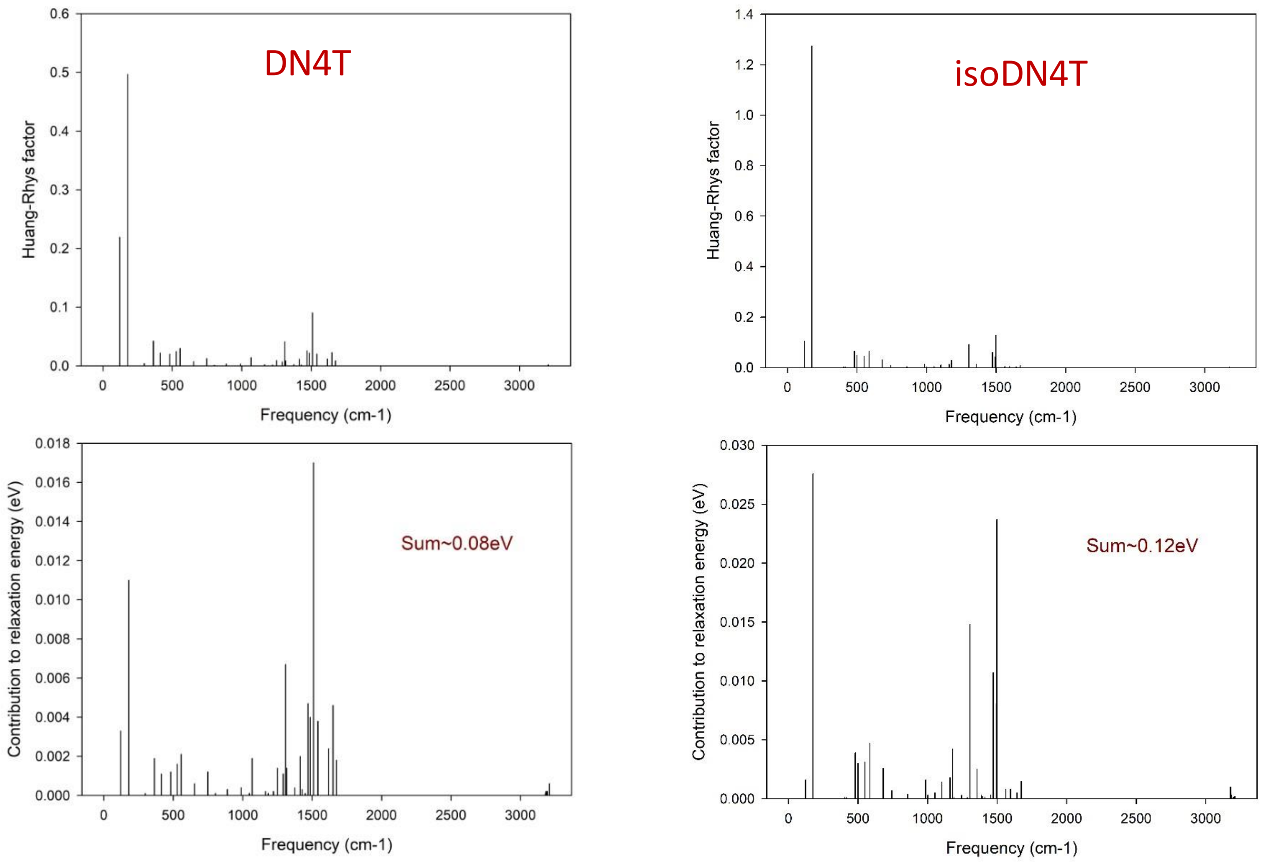

Figure S12. Huang-Rhys factor associated with each vibrational mode and corresponding energetic contribution to the total relaxation energy in DN4T and isoDN4T.

In both cases, the nuclear relaxation is dominated by a low-energy, acoustic-like, mode around 200 cm^{-1} and by a high-energy, optical, mode at 1500 cm^{-1}. These vibrations involve in-phase breathing of all the atoms in the molecule (at 200 cm^{-1}) and out-of-phase local displacements of the atoms (the ubiquitous carbon-carbon aromatic vibration at 1500 cm^{-1}). The deformation amplitude along both modes is strongly damped in the more aromatic DN4T isomer, explaining the corresponding lower relaxation energy.

5.3. Electronic couplings between dimers

To further emphasize the importance of electronic density over S atoms, we have evaluated the spatial overlap between the HOMOs in the dimers of DN4T and isoDN4T coupled in red in **Figure 4** of the main text. The overlap maps displayed in **Figure S13** and **S14** refer to the default 95% probability density convention to represent molecular orbitals, and can be compared based on extent of bonding interactions in the overlap region.[29] From the overlap pictures, it is clear that the marked decrease in the magnitude of the transfer integral going from DN4T **to** isoDN4T can be explained by the reduced wavefunction overlap over the cores in the latter case, associated with the lack of electron density on the S atoms in the HOMO of isoDN4T (**Figure 4** of the main text).

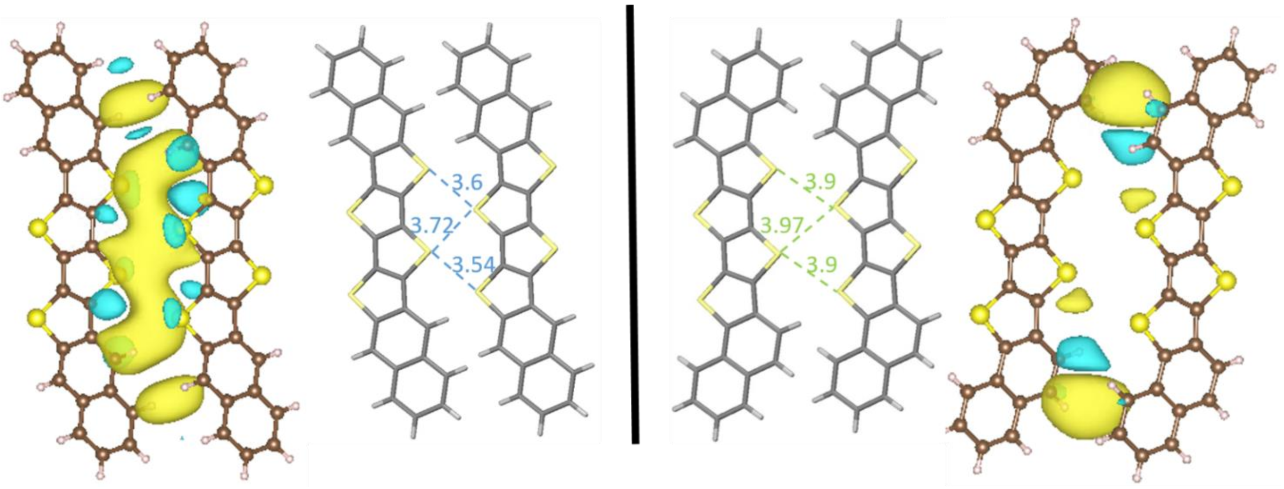

Figure S13. Overlap maps for DN4T (left, transfer integral of 61 meV) and isoDN4T (right, transfer integral of 17 meV) dimers. The two colors refer to the different signs of the LCAO coefficients in the bonding regions; blank areas correspond to regions with an antibonding pattern.

Another interesting feature of DN4T is the asymmetry in the herringbone transfer integrals (**Figure 4** of the main text), which is consistent with the absence of an inversion center in the system. While the overlap maps of two different dimers along a diagonal direction are relatively similar (**Figure S14**), there is an overall greater degree of overlap in the 99 meV coupled dimer, particularly in the internal thiophene moieties, close to the sulfur atoms.

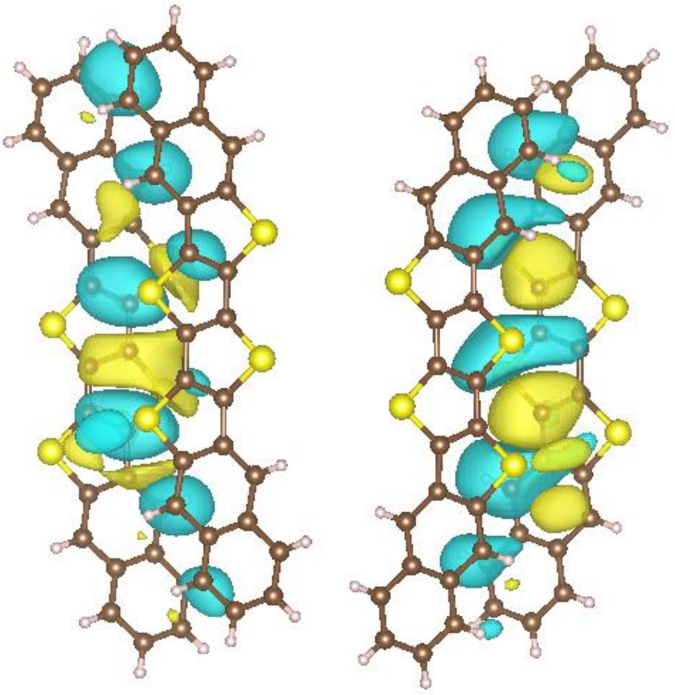

Figure S14. Overlap maps for the DN4T dimer yielding an electronic coupling of 99 meV (right) and for the DN4T dimer with a coupling of 33 meV (left).

5.4. Molecular dynamics simulations

Since previous works on rod-like semiconductors demonstrated that the relative displacements of molecules over their long axis (mostly aligned along the Z axis) is detrimental for charge transport due to a strong modulation of the transfer integral values, we have further computed the evolution of the coupling for the two dimers of DN4T displayed in **Figure S14** as a function of their relative

translation (ΔZ).[30,31] **Figure S15**, left panel shows that the transfer integrals initially at 99 meV (blue) and 33 meV (green) follow opposite variations as a function of the shift along the Z axis. This can be explained on the basis of short-distance sulfur-sulfur contacts (below 4 Å). At ΔZ=0Å (i.e., the crystal geometry) we count 4 short contacts in the 99 meV pair but only 2 in the 33 meV pair. At ΔZ = -2Å (**Figure S15, 2**), the relative number of short S-S contacts between the two pairs has swapped, and so does the relative magnitude of the transfer integrals. However, Molecular Dynamics simulations revealed that the displacement of the molecules along the Z axis does not exceed 0.5 Å. **Figure S15**, right panel shows the ΔZ distribution of the geometrical centers of mass of the dimers located in the herring bone layer along an NVT run at 300K, respecting the color code set in **Figure 4** of the main text. It appears that the maximum Z shift DN4T molecules reach is on the order of 0.22 Å for the green and blue dimer. From **Figure S15** left panel, this means the system never reaches a state where the electronic couplings are inverted (above a 1.5 Å shift in absolute value).

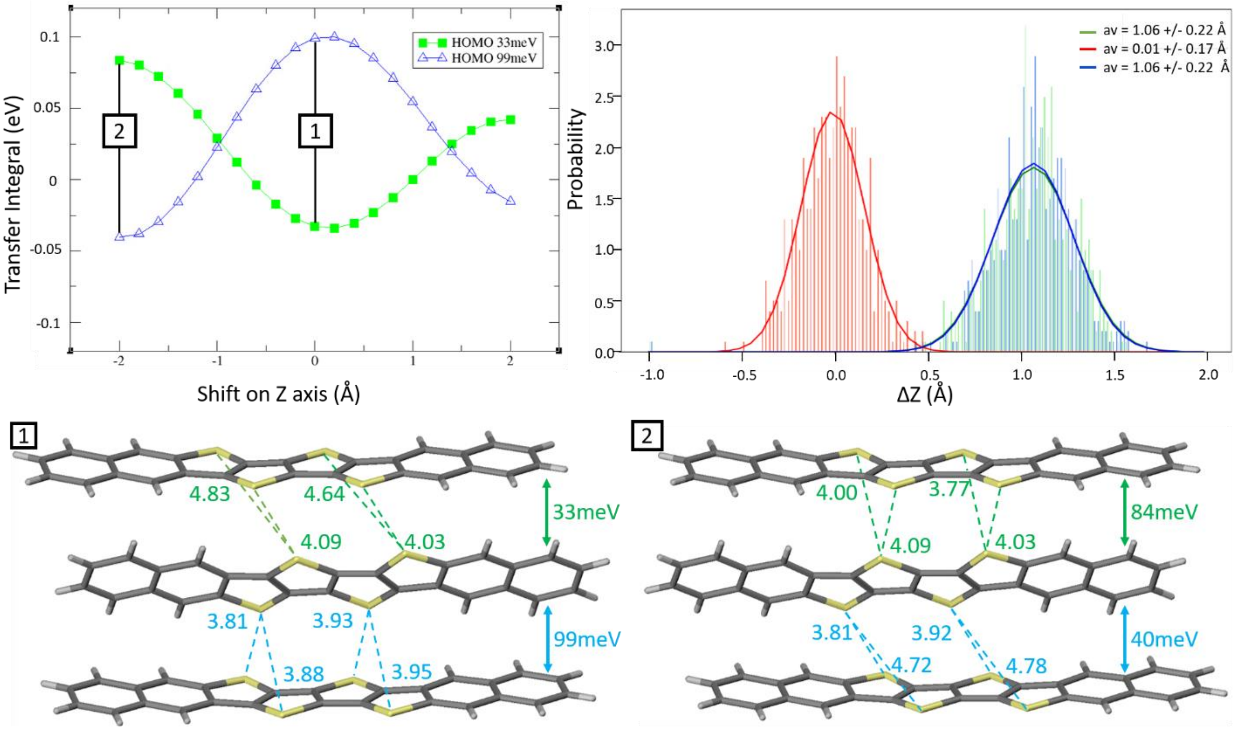

Figure S15. Left panel: values of the transfer integrals in the inequivalent dimers along the diagonal in DN4T (99 meV dimer in blue, 33 meV dimer in green). Markers 1 and 2 refer to the bottom panel where we display the molecular relative positions for the crystal structure (1, Z = 0 Å) and the system after it has been through a longitudinal shift (2, Z = 2 Å). Right panel: distributions of the Z shifts between centers of mass of important dimers along a Molecular Dynamics run (NVT – 300K).

5.5. Kinetic Monte Carlo simulation for hole mobility assessment on a pure hopping regime

In a first attempt to assess how the different connectivity in DN4T vs isoDN4T affects charge transport, we have performed kinetic Monte Carlo (kMC) simulations of hole transport assuming a purely hopping regime in the dynamic limit. We consider the two following approximations: (i) dynamic and static energetic disorder prompts the formation of charge carriers that are localized on single molecules, (ii) charge migration is slow enough so that each molecule experiences effective electronic interactions with its neighbors that equate to their time averaged values. On the basis of a qualitative analysis of the comparative amplitudes of the electronic transfer integrals versus reorganization energy, the first approximation very likely holds for isoDN4T. DN4T on the other hand might be at the limit of the validity of this hypothesis since the electronic couplings are much

closer to the reorganization energies. Additional calculations are definitively required to address this question. The second approximation results in an upper limit of the mobility in the hopping regime, such results are said to correspond to the dynamic limit. The transfer rates injected in the KMC simulations are obtained in the framework of the Marcus-Levich-Jortner formalism[32] as:

$$k_{ET} = \frac{\pi|t_{ab}|^2}{\hbar\sqrt{\pi\lambda_S k_B T}} \sum_{\nu=0}^{\infty} e^{-S} \frac{S}{\nu!} e^{\left(-\frac{(\Delta G^\circ + \lambda_S + \nu\hbar\omega_{eff})}{4\lambda_S k_B T}\right)} \quad (6)$$

A single high-frequency, effective, vibrational mode with an energy $\hbar\omega_{eff}$ estimated to 0.2 eV (related to the ~1500 cm^{-1} from **Figure S12**), accounts for quantum-mechanical nuclear tunneling (using a Huang-Rhys factor of 0.76 for DN4T and 1.15 for isoDN4T) while the low energy modes are treated classically through the external reorganization energy λ_s set equal to 0.1 eV. Part of it arises from the 200 cm^{-1} mode which results in a contribution of 0.05 eV while the contribution from the environment is roughly estimated to be also 0.05 eV.[21] Total simulation time was set to 10 ns.
The mobility of the equilibrium structure was obtained by injecting the mean electronic couplings of the corresponding dimers in the transfer rate equation. These mean values were obtained by retrieving the geometry of each dimer amongst 500 snapshots during an NVT run at 300K and computing the transfer integrals. Each snapshot is separated by 2 ps. The maximum mobility the charge can reach in this framework is defined by the dynamic limit: when the hopping rate is much lower than the frequency of the intermolecular modes, the crystal has the time explore many configurational states and the charge will most likely hop when a high transfer integral dimer configuration will appear in the thermal noise. This equates to injecting $\langle t^2 \rangle$ in the master equation. Results are displayed in **Figure 5** of the main text.

6. UPS and XPS measurements

6.1. Determination of the ionization energy by UPS

Ultraviolet photoelectron spectroscopy measurements were carried out with a UHV system (10^{-10} mbar) using the monochromatized He I_a line (21.22 eV) that was produced with a Helium discharge lamp. All spectra were recorded at room temperature using a hemispherical SPECS Phoibos 100 analyzer. Prior to the measurement, a polycrystalline gold foil was used to calibrate the detector and also to determine the energy resolution. For the valence region spectra, the pass energy was set at 5 eV that gives an energy resolution of 120 meV. The work function (Φ) values were determined from the secondary electron cutoff (SECO) spectra, measured with −10 V sample bias and a pass energy 2 eV that allows an energy resolution of 78 meV. Before the UPS measurements, the molecules were grown on Au (111) and PEDOT:PSS by thermal evaporation under ultra-high vacuum (UHV 10^{-8} mbar).
The Au (111) substrates were cleaned prior to deposition to remove any carbon contamination on the surface. For this purpose, the substrates were introduced to the UHV preparation chamber (10^{-8} -10^{-9} mbar) and two cycles of 30 min sputtering with Argon ions and annealing at 450 °C were carried out. Films of ~1-2 Å nominal thickness were then grown for both molecules which corresponds approximately to the thickness of a flat-lying DN4T (isoDN4T) monolayer. The nominal thickness was monitored *via* a quartz crystal microbalance. The UPS spectra were recorded at 45° angle between the sample and the detector as the intensity of the HOMO was maximized, due to the higher photoionization cross section for molecules lying flat on the surface, which is also the case of many other planar molecules.[33–35]
The PEDOT:PSS samples were prepared on solvent cleaned ITO (indium tin oxide) substrates. ITO substrates were exposed to UV/ozone for 10 mins and afterwards PEDOT: PSS (Ossila Al 4083) was spin-coated at 2000 rpm from an aqueous solution. The samples were then annealed in ambient conditions for 5 mins at 180°C and then introduced in UHV where a film of ~100 Å was grown. In this case the UPS spectra were taken with normal emission angle at which the intensity of the HOMO was taking the maximum value.
The ionization energy (IE) can be extracted from the UPS spectra using equation (7) :

$$IE= \Phi+HIB \qquad (7)$$

Where Φ is the work function obtained from the SECO onset as mentioned above. HIB stands for the hole injection barrier which is commonly determined from the onset of the HOMO peak.
The extracted IP values from the UPS spectra showed in **Figure S16** are reported in **Table S3** :

Sample	Φ [eV]	HIB [eV]	IE [eV]
DN4T/Au	4.61	0.94	5.55
DN4T/PEDOT:PSS	4.57	0.43	5.00
isoDN4T/Au	4.34	0.96	5.30
isoDN4T/PEDOT:PSS	4.43	0.57	5.00

Table S3. Extracted ionization energies from UPS spectra.

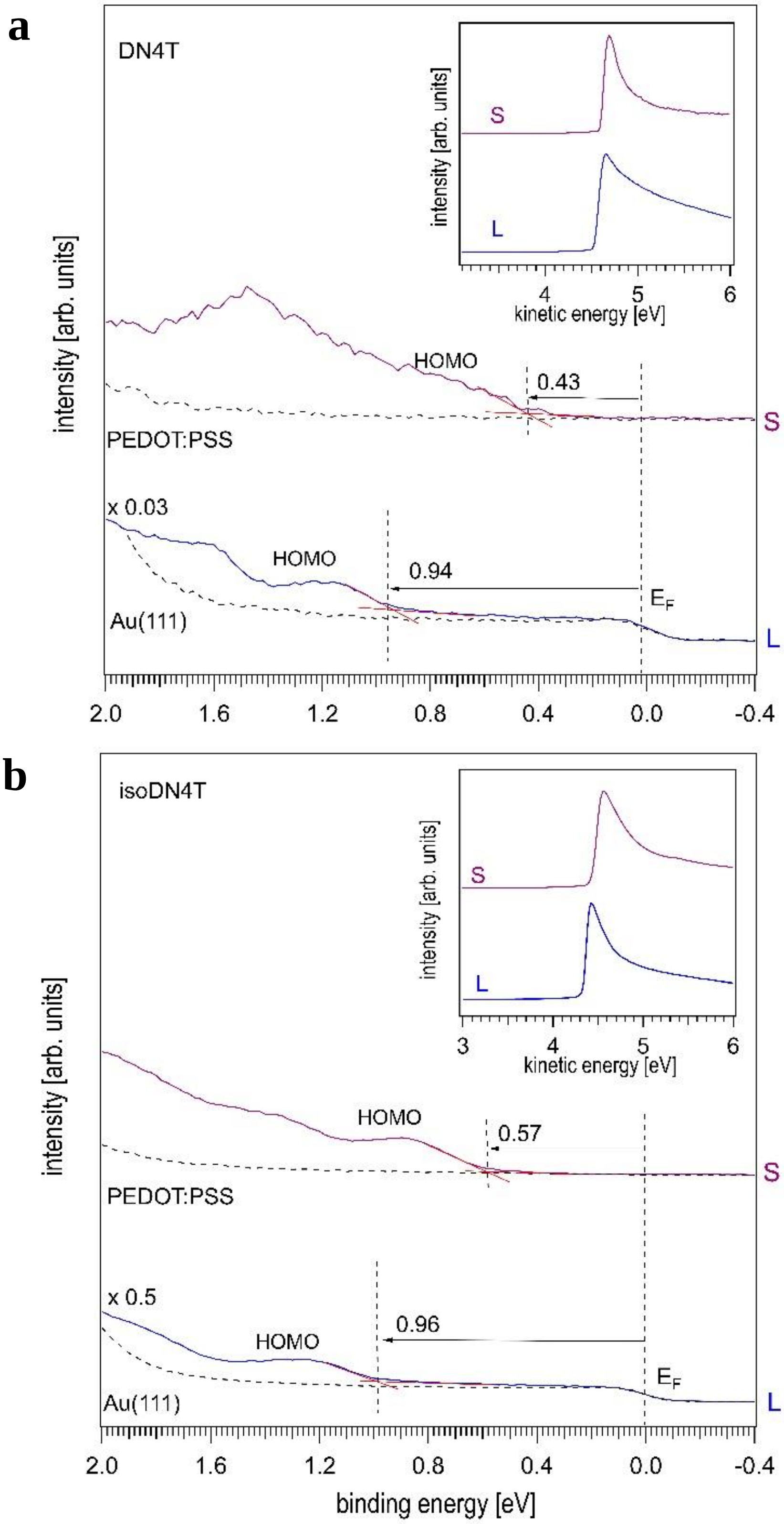

Figure S16. UPS spectra showing the valence region of (a) 1-2 Å of DN4T on Au (111) (solid blue line) and 100 Å of DN4T on PEDOT:PSS (solid red line), (b) 1-2 Å of isoDN4T on Au (111) (solid blue line) and 100 Å of isoDN4T on PEDOT:PSS (solid red line), corresponding to lying (L) and standing (S) orientations of the molecules. The dotted lines indicate the background intensity of the substrates. The inset shows the secondary-electron cutoffs used to determine the work function of the respective sample.

6.2. Evaluation of the purity of DN4T and isoDN4T by XPS

Figure S17 (a) and (b) show the x-ray photoelectron spectroscopy (XPS) survey spectra obtained using a standard hemispherical analyzer (SPECS Phoibos 100 analyzer) and a Mg X-ray source (Mg-Ka: 1254 eV). Thin films of DN4T and isoDN4T were deposited on clean Au (111) substrate by thermal evaporation in an ultra-high vacuum chamber (UHV 10^{-8} mbar). The instrumental resolution was determined by measuring the crystalline Au (111) and was 0.4 eV. The survey spectra showed contributions from Au, C and S without any presence from extrinsic contamination ultimately. A more precise analysis on the higher resolution spectra of C and S helped us to determine the stoichiometry by fitting the peaks, as shown in **Figure S18Error! Reference source not found.** (a)-(d). For the fitting of the photoelectron peaks we used Voigt functions, which are convolution of Gaussian and Lorentzian peaks to account for both the instrumental broadening but also the natural linewidth of the energy levels. A Shirley background has been subtracted to consider the background associated with the inelastically scattered electrons.[36] The peak fitting results are listed in **Table S3** and **Table S4** for the case of DN4T and isoDN4T, respectively. The stoichiometry of the DN4T and isoDN4T compounds ($C_{26}H_{12}S_4$) is given by the ratio of carbon to sulfur atoms and is therefore:

$$\frac{C}{S} = \frac{26}{4} = 6.5 \tag{8}$$

The stoichiometry determined by the XPS measurements relates to the intensity of the peaks corresponding to the carbon and sulfur orbitals that have interacted. The XPS intensities were corrected using the sensitivity factors S_C, S_S calculated from the method reported by Seah. *et.al.*[37] Therefore, the resulting stoichiometry is given by:

$$\frac{C}{S} = \frac{I_C/S_C}{I_S/S_S} \tag{9}$$

Using the results from **Table S3** and **Table S4** we determined that the stoichiometry of the two compounds is:

$$\text{DN4T: } \frac{C1s}{S2p_{3/2}} = \frac{I_C/S_C}{I_S/S_S} = 6.4 \pm 0.8 \tag{10}$$

$$\text{isoDN4T: } \frac{C1s}{S2p_{3/2}} = \frac{I_C/S_C}{I_S/S_S} = 6.9 \pm 1.0 \tag{11}$$

Seemingly, the real stoichiometric value of 6.5 lies within the interval of confidence of the experimentally determined values. However, the small discrepancy of the values can be caused due to the weak photoelectron signals associated with the S 2p energy levels, which make harder the accurate determination of the intensity of the peaks and therefore overestimate the stoichiometry as is the case of isoDN4T.

a

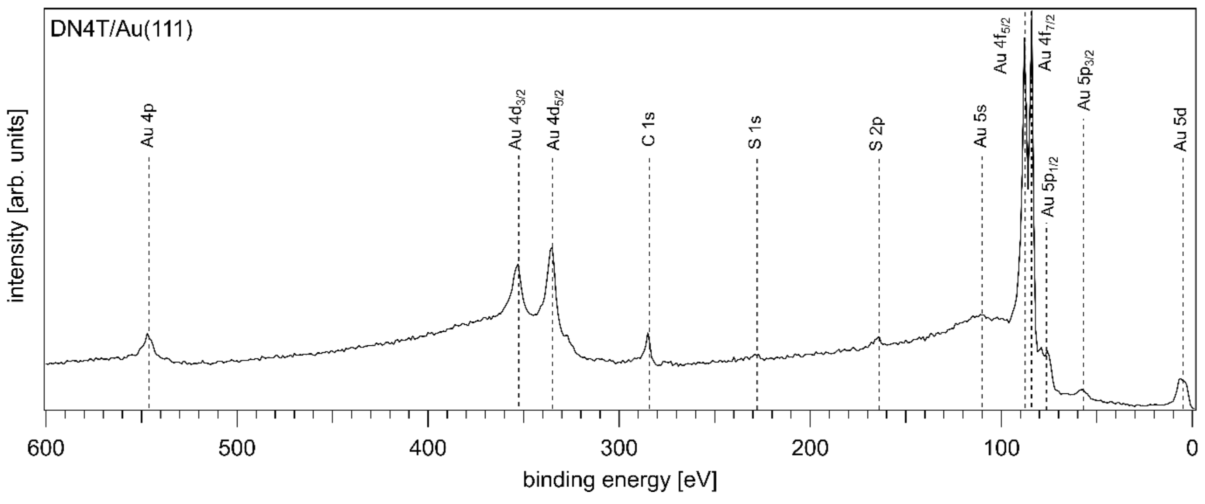

b

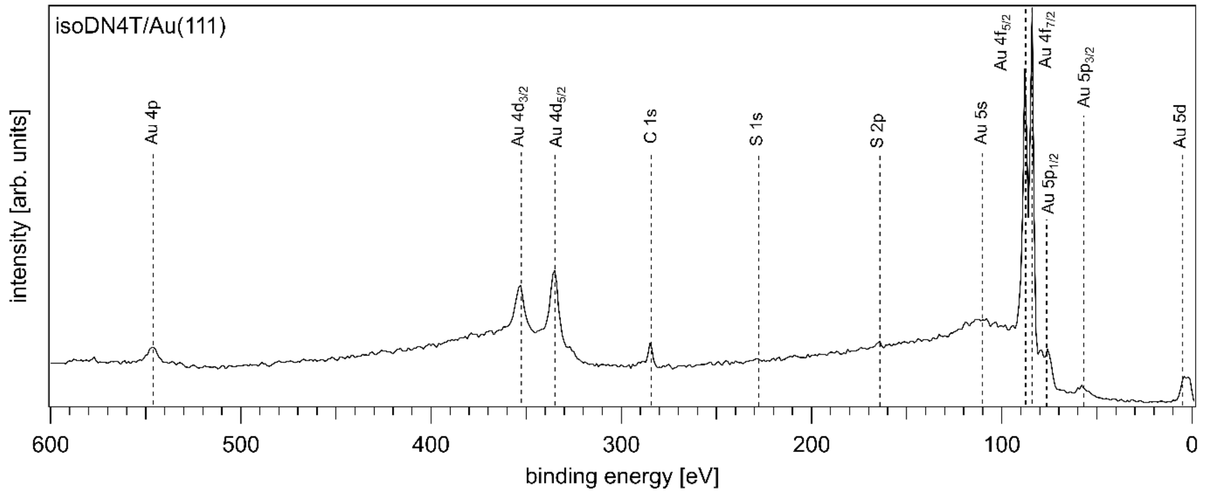

Figure S17. XPS survey spectra from DN4T (a) and isoDN4T (b) films of 10 nm nominal thickness deposited on Au (111) obtained from a Mg Ka (1254 eV). The spectra show the elemental composition of the films as the peaks correspond to the deeper energy levels of the elements which constitute the DN4T and isoDN4T compounds. The vertical dashed lines indicate all the peaks present in the survey spectrum assigned to the characteristic energy levels of the constituting elements.

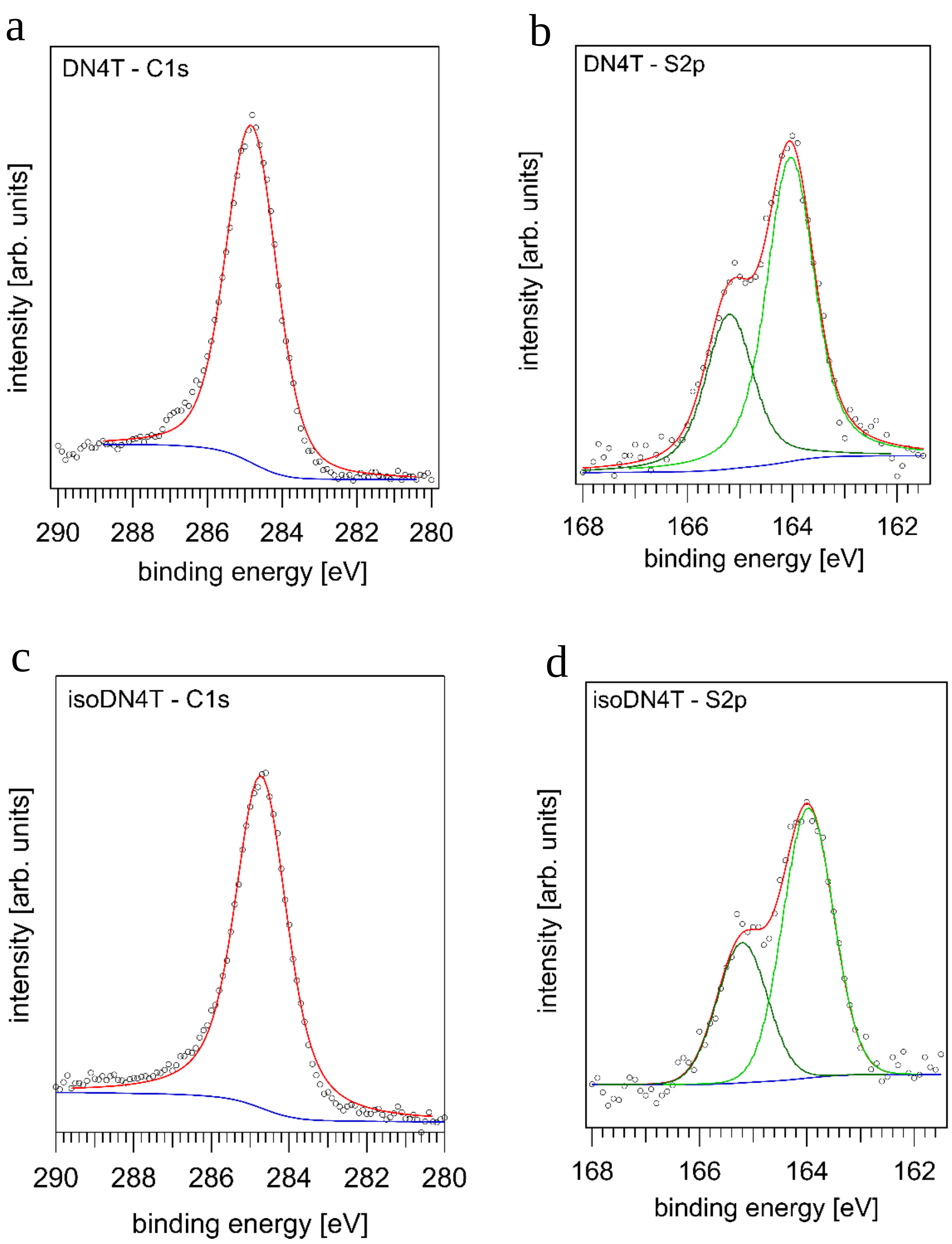

Figure S18. Higher resolution XPS spectra of DN4T C 1s (a), S 2p (b) and of isoDN4T C 1s (c), S 2p (d). The empty black circles indicate the raw data points while the red line the fitted curve. The blue line shows the Shirley background which was subtracted to evaluate the relative intensities of the peaks. Lighter and darker green lines correspond to the $2p_{3/2}$ and $2p_{1/2}$ energy levels of S due to the spin-orbit splitting.

DN4T	Location [eV]	Intensity	FWHM [eV]	Sensitivity factors
C 1s	284.8 $\pm$ 0.4	3884.3 $\pm$ 370	1.6 $\pm$ 0.4	0.000181
S $2p_{1/2}$	165.2 $\pm$ 0.4	372.1 $\pm$ 55	1.1 $\pm$ 0.4	0.000111
S $2p_{3/2}$	164.0 $\pm$ 0.4	731.8 $\pm$ 55	1.1 $\pm$ 0.4	0.000217

Table S4. Fitting parameters of C and S photoelectron peaks of DN4T.

isoDN4T	Location [eV]	Intensity	FWHM [eV]	Sensitivity factors
C 1s	284.7 $\pm$ 0.4	2746.3 $\pm$ 200	1.6 $\pm$ 0.4	0.000181
S $2p_{1/2}$	165.2 $\pm$ 0.4	246.3 $\pm$ 55	1.1 $\pm$ 0.4	0.000111
S $2p_{3/2}$	163.9 $\pm$ 0.4	474.2 $\pm$ 55	1.1 $\pm$ 0.4	0.000217

Table S5. Fitting parameters of C and S photoelectron peaks of isoDN4T.

7. IP measurements by PYS

The ionization potential (IP) of DN4T, isoDN4T and L-DBTTA powders has been measured by Photoelectron Yield Spectroscopy (PYS) at ambient conditions. Since the photoemission yield curves were recorded from powder samples, the measured IP value averages over all the possible crystallographic orientations. Photoemission yield spectroscopy was performed with a Riken Keiki spectrophotometer (Japan) model AC-2 with an energy step of 0.05 eV and a UV spot intensity of 10 nW. Average IP values over 3 trials gave 5.27 ± 0.02 eV for DN4T powder, 5.23 ± 0.01 eV for isoDN4T powder and 5.16 ± 0.01 eV.

8. Field effect transistors

8.1. Device fabrication and performances

All the thin film transistors (TFT) were fabricated on highly doped silicon wafers with 30 nm of Al_2O_3 (Christian-Albrecht University of Kiel, Institute for Electrical Engineering and Information Technology). The Al_2O_3 substrates were exposed to oxygen plasma (Diener Electronic; oxygen flow rate 20 sccm, pressure 0.50 mbar, plasma power 100 W, duration 2 min) and then immersed overnight in 1.5 mM solution of n-tetradecylphosphonic acid (TDPA, Sigma-Aldrich) in 2-propanol (Acros Organics) to obtain a 1.5-nm-thick self-assembled monolayer (SAM). Subsequently, the substrates were rinsed first in 2-propanol then in deionized water and finally in 2-propanol again and dried on a hot plate at 100 °C for 10 min. Thus, the Al_2O_3/SAM dielectric has a capacitance of 185.52 nF cm^{-2}. In the case of BC devices, gold bottom contacts were deposited by vacuum sublimation (UNIVEX 300, Leybold GmbH; pressure of ~10^{-5} mbar, deposition rate of 0.7 Å s^{-1} and nominal thickness of ca. 50 nm monitored by a quartz crystal microbalance) through a shadow mask onto the gate-dielectric substrates at room temperature. Afterwards, the substrates were immersed in a 10 mM solution of pentafluorobenzenethiol (PFBT, Alfa Aesar) in 2-propanol for 30 min, obtaining a SAM on the gold bottom contacts, then rinsed with 2-propanol and dried on a hot plate at 100 °C for 10 min. DN4T and isoDN4T were evaporated in vacuum (pressure of ~10^{-6} mbar, deposition rate of 0.5 Å s^{-1} and nominal thickness of ca. 25 nm) through a shadow mask onto the substrates which were held at different temperatures, obtaining the final devices with a channel width of 480 μm and channel length of 65, 115, 165 and 215 μm. Before the electrical measurements, the samples were left in air and in the dark overnight. In case of TC devices, DN4T and isoDN4T were deposited by vacuum sublimation (pressure of ~10^{-6} mbar, deposition rate of 0.5 Å s^{-1} and nominal thickness of ca. 25 nm) on the Al_2O_3/SAM dielectric substrates, heated at different temperatures, before the gold contacts deposition through a shadow mask (pressure of ~10^{-5} mbar, deposition rate of 7 Å s^{-1}, nominal thickness of ca. 50 nm, room temperature), obtaining the final devices with a channel width of 480 μm and channel length of 65, 115, 165 and 215 μm. In TC devices no treatment with PFBT was employed and the samples were left in air and in dark overnight, prior to the electrical measurements. In **Table S6** the devices performances obtained are reported for different substrate temperatures (from 40 to 140 °C with 20 °C steps) for DN4T and isoDN4T TFTs, in the linear (V_d=-0.5 V) and saturation (V_d=-4 V) regimes. The electrical measurements (Agilent 4155C Semiconductor Parameter Analyzer) were carried out in ambient air and at room temperature.

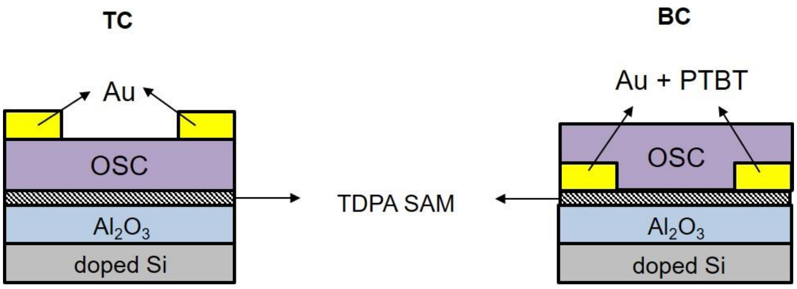

Figure S19. Schematic diagrams for TC (left) and BC (right) transistor configurations.

Transfer and output curves of best performing DN4T and isoDN4T TFTs with BC and TC configurations are reported in **Figure S20**, and charge carrier mobilities (μ) and threshold voltages (V_{th}) as a function of the substrate temperature during DN4T and isoDN4T evaporation are reported in **Table S6**. The values of μ were calculated with the conventional gradual channel model approximation using the equations (12) and (13) for the linear and saturation regimes respectively.

$$\mu_{lin} = \frac{\partial I_d}{\partial V_g} \frac{L}{C_i \, W \, V_d} \tag{12}$$

$$\mu_{sat} = \left(\frac{\partial I_d}{\partial V_g}\right)^2 \frac{2L}{C_i \, W} \tag{13}$$

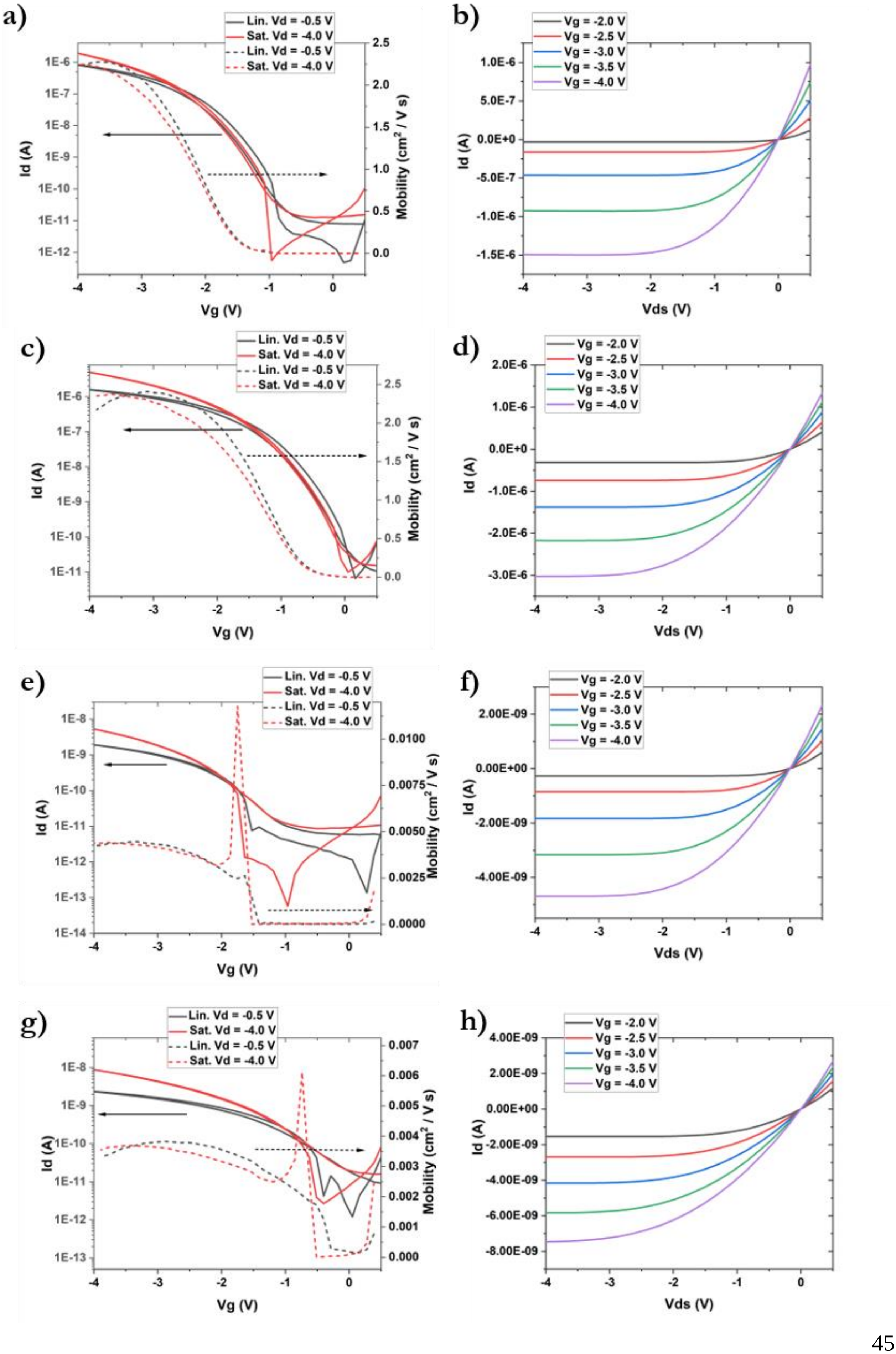

a)
Lin. Vd = -0.5 V
Sat. Vd = -4.0 V
Lin. Vd = -0.5 V
Sat. Vd = -4.0 V
Id (A)
Mobility (cm^2 / V s)
Vg (V)
b)
Vg = -2.0 V
Vg = -2.5 V
Vg = -3.0 V
Vg = -3.5 V
Vg = -4.0 V
Id (A)
Vds (V)
c)
Lin. Vd = -0.5 V
Sat. Vd = -4.0 V
Lin. Vd = -0.5 V
Sat. Vd = -4.0 V
Id (A)
Mobility (cm^2 / V s)
Vg (V)
d)
Vg = -2.0 V
Vg = -2.5 V
Vg = -3.0 V
Vg = -3.5 V
Vg = -4.0 V
Id (A)
Vds (V)
e)
Lin. Vd = -0.5 V
Sat. Vd = -4.0 V
Lin. Vd = -0.5 V
Sat. Vd = -4.0 V
Id (A)
Mobility (cm^2 / V s)
Vg (V)
f)
Vg = -2.0 V
Vg = -2.5 V
Vg = -3.0 V
Vg = -3.5 V
Vg = -4.0 V
Id (A)
Vds (V)
g)
Lin. Vd = -0.5 V
Sat. Vd = -4.0 V
Lin. Vd = -0.5 V
Sat. Vd = -4.0 V
Id (A)
Mobility (cm^2 / V s)
Vg (V)
h)
Vg = -2.0 V
Vg = -2.5 V
Vg = -3.0 V
Vg = -3.5 V
Vg = -4.0 V
Id (A)
Vds (V)

Figure S20. (a) Transfer and (b) output characteristics of DN4T with TC structure, (c) transfer and (d) output characteristics of DN4T with BC structure, (e) transfer and (f) output characteristics of isoDN4T with TC structure, (g) transfer and (h) output characteristics of isoDN4T with BC structure. In the transfer curves the solid lines represent the drain current and the dashed lines the mobility. The TFTs have W/L = 480/215 µm.

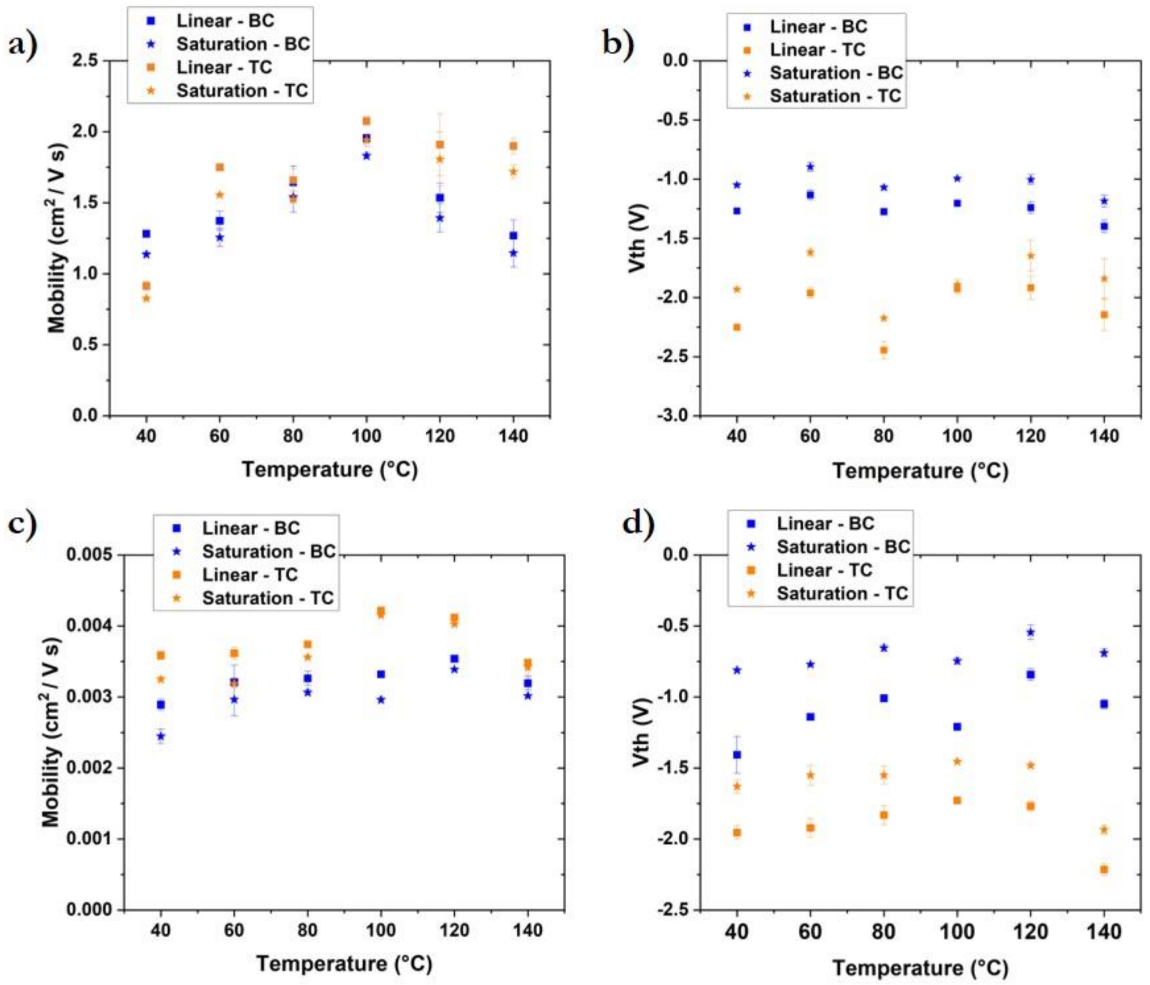

Figure S21. (a) Mobility and (b) threshold voltage as a function of the substrate temperature for DN4T TFTs, (c) Mobility and (d) threshold voltage as a function of the substrate temperature for isoDN4T TFTs.

Table S6. Performance of DN4T and isoDN4T TFTs. In the linear regime $V_d = -0.5V$ and in saturation regime $V_d = -4.0$ V.

8.2. Contact Resistance

Evaluation of the channel-width-normalized contact resistance (R_cW), using the transmission line method (TLM)[38,39] at a substrate temperature of 100 °C for DN4T and of 120 °C for isoDN4T TFTs was carried out following equation (14) :

$$R_{tot}W = R_cW + \frac{1}{\mu_0}\frac{L}{C_i\,(V_g - V_{th})} \tag{14}$$

where R_{tot} is the total resistance of the transistor and R_c is the contact resistance. The model assumes that the channel resistance, but not the contact resistance, depends on the channel length. Hence, plotting the total resistance of the transistor using different channel length, it is

Compound	Device	Substrate T [C]	μ [cm^2 V^{-1} s^{-1}]		V_{th} [V]		SS [V dec^{-1}]		ON/OFF	
	Structure		Linear	Saturation	Linear	Saturation	Linear	Saturation	Linear	Saturation
DN4T	TC	40	0.9 ± 0.03	0.8 ± 0.01	-2.3 ± 0.01	-1.9 ± 0.01	0.19	0.08	~ 5 E5	~ 7 E5
		60	1.7 ± 0.03	1.6 ± 0.01	-2.0 ± 0.05	-1.6 ± 0.03	0.11	0.10	~ 2 E6	~ 6 E5
		80	1.7 ± 0.08	1.5 ± 0.01	-2.4 ± 0.07	-2.2 ± 0.01	0.15	0.10	~ 7 E5	~ 4 E6
		100	2.1 ± 0.03	1.9 ± 0.05	-1.9 ± 0.04	-1.9 ± 0.05	0.14	0.07	~ 9 E5	~ 2 E6
		120	1.9 ± 0.22	1.8 ± 0.19	-1.9 ± 0.10	-1.6 ± 0.13	0.13	0.08	~ 1 E6	~ 3 E6
		140	1.9 ± 0.05	1.7 ± 0.05	-2.1 ± 0.13	-1.8 ± 0.17	0.17	0.09	~ 9 E5	~ 2 E6
	BC	40	1.3 ± 0.02	1.1 ± 0.01	-1.3 ± 0.02	-1.1 ± 0.01	0.12	0.16	~ 1 E5	~ 2 E5
		60	1.4 ± 0.07	1.3 ± 0.06	-1.1 ± 0.04	-0.9 ± 0.04	0.14	0.13	~ 1 E5	~ 3 E5
		80	1.6 ± 0.11	1.5 ± 0.11	-1.3 ± 0.02	-1.1 ± 0.02	0.13	0.13	~ 6 E5	~ 5 E5
		100	2.0 ± 0.02	1.8 ± 0.02	-1.2 ± 0.01	-1.0 ± 0.01	0.13	0.14	~ 3 E5	~ 5 E5
		120	1.5 ± 0.10	1.4 ± 0.10	-1.2 ± 0.05	-1.0 ± 0.04	0.09	0.12	~ 4 E5	~ 4 E5
		140	1.3 ± 0.11	1.1 ± 0.10	-1.4 ± 0.05	-1.2 ± 0.05	0.11	0.11	~ 1 E6	~ 5 E5
isoDN4T	TC	40	(3.6 ± 0.06) E-3	(3.3 ± 0.04) E-3	-2.0 ± 0.05	-1.6 ± 0.05	0.19	0.08	~ 6 E3	~ 3 E4
		60	(3.6 ± 0.08) E-3	(3.2 ± 0.06) E-3	-1.9 ± 0.07	-1.6 ± 0.07	0.18	0.09	~ 7 E3	~ 2 E4
		80	(3.7 ± 0.01) E-3	(3.6 ± 0.01) E-3	-1.8 ± 0.07	-1.6 ± 0.06	0.22	0.13	~ 4 E3	~ 2 E4
		100	(4.2 ± 0.01) E-3	(4.2 ± 0.01) E-3	-1.7 ± 0.01	-1.5 ± 0.01	0.26	0.11	~ 4 E3	~ 3 E4
		120	(4.1 ± 0.05) E-3	(4.0 ± 0.01) E-3	-1.8 ± 0.03	-1.5 ± 0.02	0.23	0.11	~ 4 E3	~ 2 E4
		140	(3.5 ± 0.04) E-3	(3.4 ± 0.11) E-3	-2.2 ± 0.04	-1.9 ± 0.03	0.31	0.12	~ 3 E3	~ 2 E4
	BC	40	(2.9 ± 0.08) E-3	(2.5 ± 0.10) E-3	-1.4 ± 0.13	-0.8 ± 0.01	0.15	0.25	~ 4 E2	~ 6 E2
		60	(3.2 ± 0.24) E-3	(3.0 ± 0.22) E-3	-1.1 ± 0.01	-0.8 ± 0.01	0.24	0.14	~ 7 E2	~ 3 E3
		80	(3.3 ± 0.05) E-3	(3.0 ± 0.04) E-3	-1.2 ± 0.01	-0.7 ± 0.02	0.20	0.17	~ 9 E2	~ 2 E3
		100	(3.3 ± 0.10) E-3	(3.1 ± 0.04) E-3	-1.0 ± 0.01	-0.7 ± 0.02	0.20	0.19	~ 6 E2	~ 3 E3
		120	(3.5 ± 0.04) E-3	(3.4 ± 0.02) E-3	-0.8 ± 0.04	-0.5 ± 0.05	0.17	0.16	~ 1 E3	~ 3 E3
		140	(3.2 ± 0.09) E-3	(3.0 ± 0.02) E-3	-1.1 ± 0.03	-0.7 ± 0.03	0.28	0.13	~ 2 E3	~ 4 E3

possible to calculate the RcW and μ_0, through a linear fitting. Both for the BC and TC devices of DN4T and isoDN4T, TLM was applied (**Figure S22**) and RcW and μ_0 were extracted (**Table S7**) for the highest measured source-gate voltage (-4 V) and in linear regime (V_d=-0.5 V). In all the linear fittings the adjusted R2 values ≥ 0.98.

Differences in terms of threshold voltages observed between BC and TC configurations follow the differences observed in the corresponding contact resistance results. The strong difference in contact resistance for isomers which have very similar injection barriers can be mainly

explained by their dependance on the charge carrier mobility.[40–42] Considering that the injection barrier is almost similar for the two isomers due to comparable HOMO and LUMO energies, μ plays a crucial role in determining the contact resistance. Therefore, a higher μ would turn out in a lower R_cW, in both BC and TC devices.

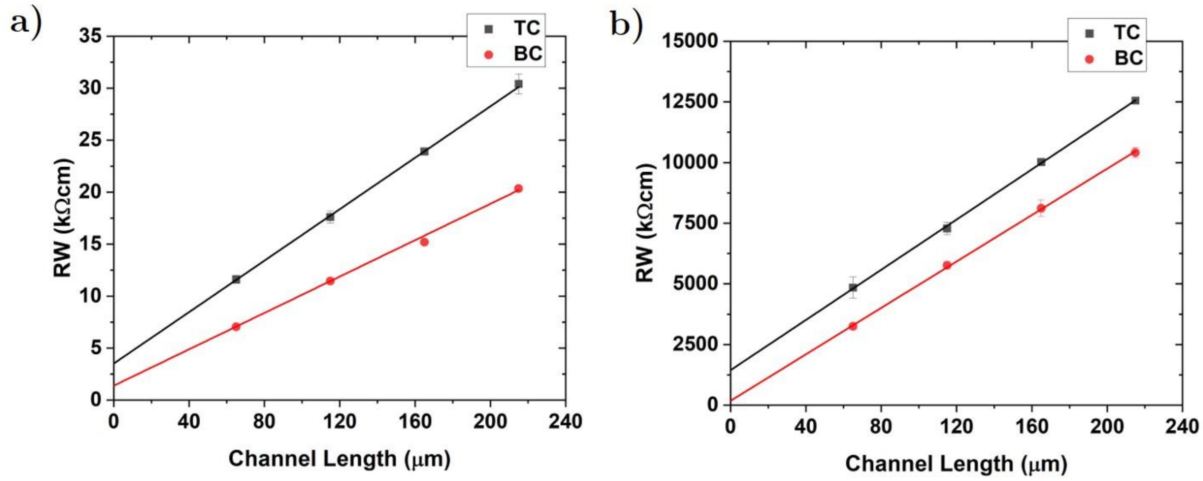

Figure S22: TLM for (a) bottom and top contact structure of DN4T TFTs at a substrate temperature of 100 °C and (b) for bottom and top contact structure of isoDN4T TFTs at a substrate temperature of 120 °C. The exact channel lengths are 65, 115, 165 and 215 μm.

Compound	Device structure	Substrate T [°C]	μ_0 [cm^2 V^{-1} s^{-1}]	RcW [kΩ cm]
DN4T	TC	100	2.4 ± 0.06	3.5 ± 0.18
	BC	100	2.2 ± 0.11	1.4 ± 0.24
isoDN4T	TC	120	(4.8 ± 0.23) 10^{-3}	1440 ± 140
	BC	120	(3.5 ± 0.42) 10^{-3}	212 ± 96

Table S7. Intrinsic mobility and channel-width-normalized contact resistance for bottom and top contact structures of DN4T and isoDN4T TFTs.

8.3. Thin film morphologies

The thin films morphologies were evaluated through atomic force microscopy (AFM, Brucker Dimension Icon) in tapping mode with Olympus OMCL-AC240TS-R3. DN4T and isoDN4T thin films of ca. 25 nm were evaporated on Al_2O_3 substrates treated with TDPA, using the previously mentioned procedure. The substrates were held at 40, 100 and 140 °C. **Figure S23** shows the effect of temperature on DN4T and isoDN4T thin-films morphology. A terrace pattern covered by needle-shaped crystals arises from the images at all substrate temperatures. Particularly for DN4T, the quantity of these three-dimensional needle-shaped crystals is higher compared to isoDN4T samples, covering the terrace structures and making difficult to perform the image analysis. In **Table S8** are reported the estimated height of the terrace steps and the grain diameters are reported. Remarkably, the longest grain diameter is achieved at a substrate temperature of 100 °C for both DN4T and isoDN4T samples, which correspond to the highest mobility values for both molecules (**Figure S24**), highlighting that the field-effect mobility is enhanced with increasing the grain size. Moreover, the height of the terrace steps is close to the molecular length of DN4T and isoDN4T, suggesting that in the crystalline films the molecules are oriented approximately upright with respect to the substrate. This observation was furtherly confirmed by XRD patterns recorded on DN4T and isoDN4T thin films (Rigaku SmartLab, Cu Kα radiation). The same samples used for AFM measurements were employed for XRD

measurements, particularly the ones deposited at substrate temperature of 100 °C. From the XRD patterns (**Figure S25**), the Bragg peaks along the out-of-plane direction were indexed and assigned to {001} and the average d-spacing was calculated. The averaged d-spacing is (20.2 ± 0.5) Å and (19.2 ± 0.1) Å for DN4T and isoDN4T respectively, which corresponds to the molecular long axis obtained from single crystal XRD and corroborating that the thin films adopt a layer-by-layer structures with the molecules standing approximately upright on their long axis respect to the substrate surface.

Compound	Substrate T [°C]	Terrace height [Å]	Grain diameter [nm]
DN4T	40	20 ± 2	145 ± 30
	100	20 ± 1	250 ± 60
	140	18 ± 2	200 ± 15
isoDN4T	40	18 ± 1	190 ± 30
	100	18 ± 2	460 ± 75
	140	20 ± 2	320 ± 55

Table S8. Terrace height and grain diameter extracted from AFM images for DN4T and isoDN4T thin films, at different substrate temperature.

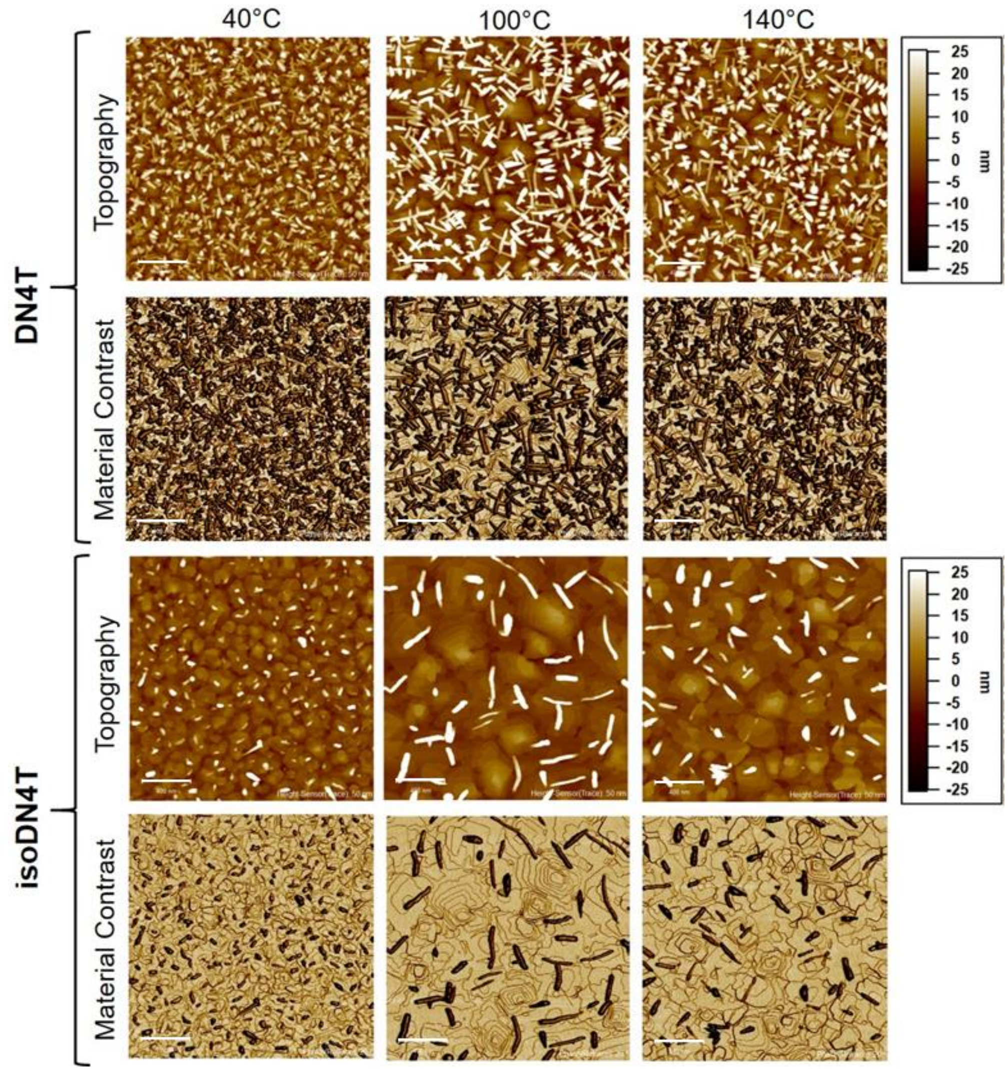

Figure S23. AFM topography and material phase contrast images of DN4T and isoDN4T thin-films (thickness ca. 25 nm) deposited Al_2O_3 treated with TDPA, at a different substrate temperature. All images represent 2 μm × 2 μm and bottom left scale bars represent 400 nm.

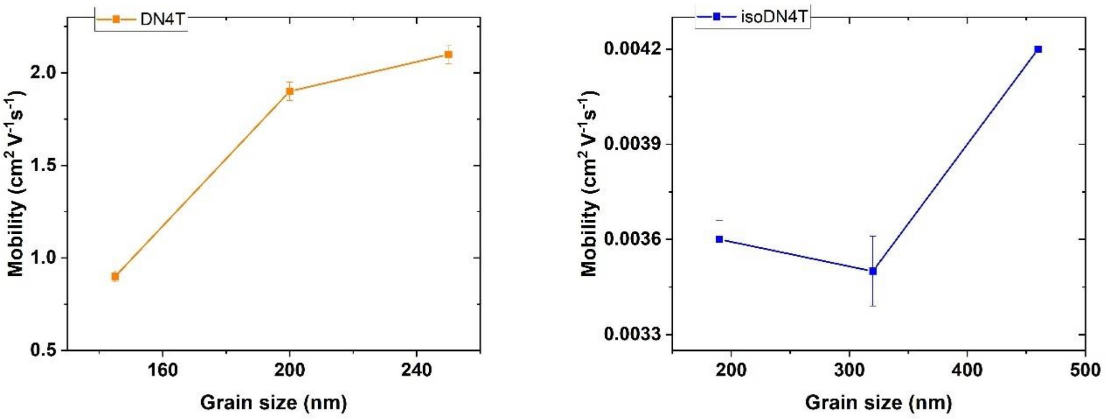

Figure S24. Mobility of TC devices as a function of the grain size for DN4T (left) and isoDN4T (right).

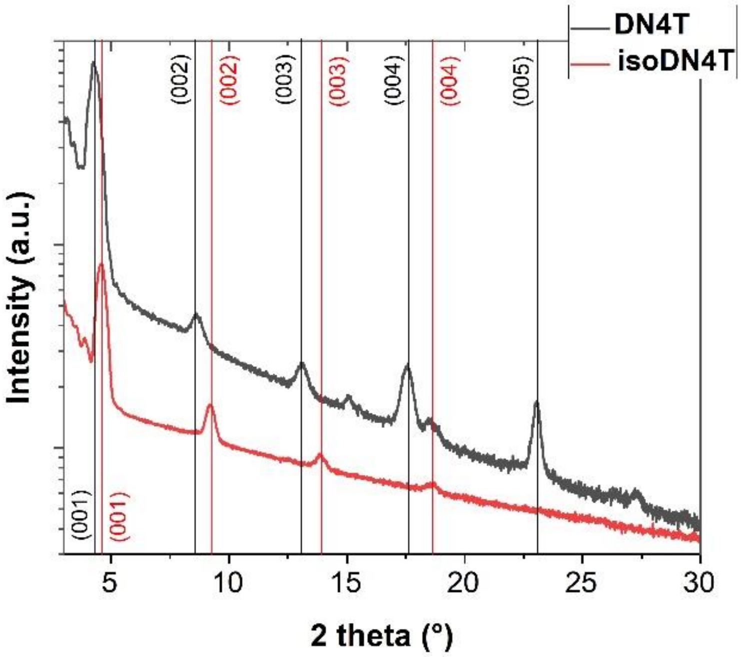

Figure S25. XRD patterns of DN4T and isoDN4T films deposited at substrate temperature of 100 °C with a thickness of 25 nm.

References

[1] F. Xiao, S. Chen, J. Tian, H. Huang, Y. Liu, G.-J. Deng, *Green Chem.* **2016**, *18*, 1538.
[2] L. Palatinus, G. Chapuis, *J Appl Cryst* **2007**, *40*, 786.
[3] G. M. Sheldrick, *Acta Cryst C* **2015**, *71*, 3.
[4] A. L. Spek, *Acta Cryst D* **2009**, *65*, 148.
[5] L. J. Farrugia, *J Appl Cryst* **2012**, *45*, 849.
[6] R. E. Marsh, *Acta Cryst B* **1986**, *42*, 193.
[7] M. A. Spackman, J. J. McKinnon, *CrystEngComm* **2002**, *4*, 378.
[8] M. A. Spackman, D. Jayatilaka, *CrystEngComm* **2009**, *11*, 19.
[9] M. J. Turner, J. J. McKinnon, S. K. Wolff, D. J. Grimwood, P. R. Spackman, D. Jayatilaka, M. A. Spackman, *CrystalExplorer17, University of Western Australia, 2017*, **n.d.**
[10] E. Prince, Internationale Union für Kristallographie, Eds. , *Mathematical, Physical and Chemical Tables*, Kluwer Acad. Publ, Dordrecht, **2011**.
[11] J. J. McKinnon, M. A. Spackman, A. S. Mitchell, *Acta Cryst B* **2004**, *60*, 627.
[12] M. A. Neumann, *J Appl Cryst* **2003**, *36*, 356.
[13] G. E. Engel, S. Wilke, O. König, K. D. M. Harris, F. J. J. Leusen, *J Appl Cryst* **1999**, *32*, 1169.
[14] G. S. Pawley, *J Appl Cryst* **1981**, *14*, 357.
[15] A. Altomare, C. Cuocci, C. Giacovazzo, A. Moliterni, R. Rizzi, N. Corriero, A. Falcicchio, *J Appl Cryst* **2013**, *46*, 1231.
[16] H. M. Rietveld, *J Appl Cryst* **1969**, *2*, 65.
[17] R. A. Young, International Workshop on the Rietveld Method, Eds. , *The Rietveld Method: Product of the International Workshop on the Rietveld Method in Petten, The Netherlands, 13 - 15 June 1989*, Oxford University Press, Oxford, **1993**.
[18] A. Le Bail, H. Duroy, J. L. Fourquet, *Materials Research Bulletin* **1988**, *23*, 447.
[19] C. F. Macrae, I. J. Bruno, J. A. Chisholm, P. R. Edgington, P. McCabe, E. Pidcock, L. Rodriguez-Monge, R. Taylor, J. van de Streek, P. A. Wood, *J Appl Cryst* **2008**, *41*, 466.
[20] C. F. Macrae, P. R. Edgington, P. McCabe, E. Pidcock, G. P. Shields, R. Taylor, M. Towler, J. van de Streek, *J Appl Cryst* **2006**, *39*, 453.
[21] N. G. Martinelli, J. Idé, R. S. Sánchez-Carrera, V. Coropceanu, J.-L. Brédas, L. Ducasse, F. Castet, J. Cornil, D. Beljonne, *J. Phys. Chem. C* **2010**, *114*, 20678.
[22] G. te Velde, F. M. Bickelhaupt, E. J. Baerends, C. Fonseca Guerra, S. J. A. van Gisbergen, J. G. Snijders, T. Ziegler, *J. Comput. Chem.* **2001**, *22*, 931.
[23] D. B. Boyd, K. B. Lipkowitz, *Reviews in Computational Chemistry. Vol. 15 Vol. 15*, Wiley-VCH, New York, New York, **2000**.
[24] L. S. Dodda, I. Cabeza de Vaca, J. Tirado-Rives, W. L. Jorgensen, *Nucleic Acids Research* **2017**, *45*, W331.
[25] S. Plimpton, *Journal of Computational Physics* **1995**, *117*, 1.
[26] M. N. Gueye, A. Vercouter, R. Jouclas, D. Guérin, V. Lemaur, G. Schweicher, S. Lenfant, A. Antidormi, Y. Geerts, C. Melis, J. Cornil, D. Vuillaume, *Nanoscale* **2021**, *13*, 3800.
[27] A. Troisi, G. Orlandi, *J. Phys. Chem. A* **2006**, *110*, 4065.
[28] V. Coropceanu, J. Cornil, D. A. da Silva Filho, Y. Olivier, R. Silbey, J.-L. Brédas, *Chem. Rev.* **2007**, *107*, 926.
[29] J. L. Brédas, J. P. Calbert, D. A. da S. Filho, J. Cornil, *PNAS* **2002**, *99*, 5804.
[30] G. Schweicher, G. D’Avino, M. T. Ruggiero, D. J. Harkin, K. Broch, D. Venkateshvaran, G. Liu, A. Richard, C. Ruzié, J. Armstrong, A. R. Kennedy, K. Shankland, K. Takimiya, Y. H. Geerts, J. A. Zeitler, S. Fratini, H. Sirringhaus, *Advanced Materials* **2019**, *31*, 1902407.

[31] N. G. Martinelli, Y. Olivier, S. Athanasopoulos, M.-C. R. Delgado, K. R. Pigg, D. A. da S. Filho, R. S. Sánchez- Carrera, E. Venuti, R. G. D. Valle, J.-L. Brédas, D. Beljonne, J. Cornil, *ChemPhysChem* **2009**, *10*, 2265.
[32] S. Chaudhuri, S. Hedström, D. D. Méndez-Hernández, H. P. Hendrickson, K. A. Jung, J. Ho, V. S. Batista, *J. Chem. Theory Comput.* **2017**, *13*, 6000.
[33] T. Ules, D. Lüftner, E. M. Reinisch, G. Koller, P. Puschnig, M. G. Ramsey, *Physical Review B* **2014**, *90*, DOI 10.1103/PhysRevB.90.155430.
[34] N. Ueno, A. Kitamura, K. K. Okudaira, T. Miyamae, Y. Harada, S. Hasegawa, H. Ishii, H. Inokuchi, T. Fujikawa, T. Miyazaki, K. Seki, *The Journal of Chemical Physics* **1997**, *107*, 2079.
[35] N. Ueno, K. Suzuki, S. Hasegawa, K. Kamiya, K. Seki, H. Inokuchi, *The Journal of Chemical Physics* **1993**, *99*, 7169.
[36] J. Végh, *Journal of Electron Spectroscopy and Related Phenomena* **2006**, *151*, 159.
[37] M. P. Seah, I. S. Gilmore, S. J. Spencer, *Journal of Electron Spectroscopy and Related Phenomena* **2001**, *120*, 93.
[38] P. V. Necliudov, M. S. Shur, D. J. Gundlach, T. N. Jackson, *Solid-State Electronics* **2003**, *47*, 259.
[39] C. Rolin, E. Kang, J.-H. Lee, G. Borghs, P. Heremans, J. Genoe, *Nat Commun* **2017**, *8*, 14975.
[40] Y. Hu, D. X. Cao, A. T. Lill, L. Jiang, C.-A. Di, X. Gao, H. Sirringhaus, T.-Q. Nguyen, *Adv. Electron. Mater.* **2018**, *4*, 1800175.
[41] B. H. Hamadani, D. Natelson, *Appl. Phys. Lett.* **2004**, *84*, 443.
[42] D. Natali, M. Caironi, *Adv. Mater.* **2012**, *24*, 1357.
